# DNA Binding, DNA Photocleavage, Molecular Docking Studies and Photo-Induced Effect on Melanoma Cells of 2-Methyl-3-OR Quinazolinone Derivatives

**DOI:** 10.3390/biom16040551

**Published:** 2026-04-08

**Authors:** Chrysoula Mikra, Stella Malichetoudi, Dimitrios Arampatzis, Ioanna Laskari, Maria Koffa, Ewelina Wieczorek-Szweda, Katerina R. Katsani, George Psomas, Konstantina C. Fylaktakidou

**Affiliations:** 1 Laboratory of Organic Chemistry, Faculty of Chemistry, Aristotle University of Thessaloniki, GR-54124 Thessaloniki, Greece; chrmikgeo@chem.auth.gr; 2 Laboratory of Cellular Biology, Molecular Biology and Genetics Department, Democritus University of Thrace, University Campus, GR-68100 Alexandroupolis, Greece; stelmali1@mbg.duth.gr (S.M.); dimitrios.b.arabatzis@gmail.com (D.A.); ioanna.elkat@gmail.com (I.L.); mkoffa@mbg.duth.gr (M.K.); 3 Department of Functional Nanostructures Synthesis, Faculty of Chemistry, Adam Mickiewicz University, PL-61614 Poznań, Poland; ewelina.wieczorek-szweda@amu.edu.pl; 4 Laboratory of Biochemistry and Molecular Virology, Molecular Biology and Genetics Department, Democritus University of Thrace, Dragana, GR-68100 Alexandroupolis, Greece; kkatsani@mbg.duth.gr; 5 Laboratory of Inorganic Chemistry, Department of Chemistry, Aristotle University of Thessaloniki, GR-54124 Thessaloniki, Greece; gepsomas@chem.auth.gr

**Keywords:** quinazolinone, hydroxamic acid, DNA binding, DNA photocleavage, singlet oxygen release, molecular docking, melanoma cells, HaCat cells

## Abstract

Thirty 2-methyl-quinazolinone fussed hydroxamic acids (3-OH) and their 3-OEt and 3-OBn derivatives were evaluated for their affinity towards calf-thymus (CT) DNA using UV-vis absorption, viscosity and fluorescence spectroscopy. DNA photocleavage activity was assessed by incubating the compounds with plasmid DNA followed by UV-A and visible light irradiation, which enabled identification of the most potent derivatives active at concentrations of 100 nΜ and 10 μΜ, respectively. Mechanistic studies on the most active compounds indicated the formation of oxygen radical species and a decrease in efficiency under argon. Measurements of singlet oxygen release verified these findings. Molecular docking studies provided further insight into the interactions between the compounds and DNA. UV-A irradiation of the most potent DNA photocleavers in three cell lines, two malignant melanoma lines (A375 and COLO-800) and the immortalized keratinocyte line HaCaT, identified three derivatives that, at a concentration up to 10 μΜ, reduced cell viability by approximately 50%. Taken together, these results indicate that these 2-methylquinazolinone-based hydroxamic acid derivatives are promising candidates for the development of photodynamic therapy agents.

## 1. Introduction

Hydroxamic acids (HMAs) are compounds with the general formula R-CO-NH-OH or R-CO-NR’-OH. Due to their ability to chelate metals, they are commonly found in natural products, mainly produced by bacteria as part of their strategy to acquire essential metals required for survival [1,2]. As the metal is mainly iron, such compounds are referred to as “siderophores” [3,4]. Interestingly, several microorganisms synthesize toxic HMAs, known as sideromycins or microcins, which exhibit antimicrobial activity, enabling them to compete with other microbial species in their ecological niche [4,5]. Inverting this concept, antibacterial drugs containing an HMA residue loaded with Fe(III) (Ferrisiderophores) act as “Trojan Horses”, exploiting bacterial uptake systems for siderophores while delivering a lethal bactericidal payload [1,6].

Three histone deacetylase (HDAC) inhibitors, Belinostat, Panobinostat and Vorinostat, have been approved by the FDA for the treatment of T-cell lymphoma or multiple myeloma, establishing the HMA fragment as a validated pharmacophore [7,8,9]. Beyond their antimicrobial activity, HMA-containing compounds display anticancer, antimalarial and antitubercular properties, and several members of this class have found (bio)technological applications in drug delivery—vaccine development upon conjugation to immunogenic carrier proteins and biosensing [10,11,12,13,14,15].

Despite their potential as drugs, HMAs have shown mutagenic properties in vitro, as they can interact with DNA and may be transformed into toxic isocyanates via the Lossen rearrangement [9,16,17]. In this case, the presence of the H atom on the nitrogen facilitates this transformation, as does the OH group in “free” HMAs. By contrast, several benzyl and allyl hydroxamates (non-free HMAs) have been synthesized and found to exhibit antitubercular [18], anticancer [19] and amyloid beta (Aβ42) aggregation inhibition [20].

**Figure 1:**
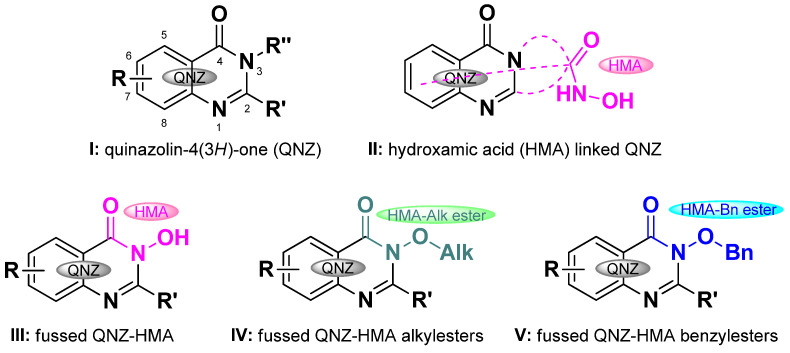
Structure of QNZ (**I**), HMA-linked QNZ (**II**), fussed free QNZ-HMA (**III**), and alkyl and benzyl substituted QNZ-HMA esters (**IV** and **V**, respectively).

Quinazolinones (QNZs) are versatile heterocyclic pharmacophores that are often described as “privileged structures” [21,22,23,24,25]. They are found in nature [24,26,27,28,29], and their core scaffold can also be readily synthesized in the laboratory [22], allowing derivatization at least at six positions; five C-linked (2-,5-,6-,7-,8-) and one N-linked (3-) positions (**I**, Figure 1). QNZ-HMAs (**II**, Figure 1) bearing the HMA residue as a pendant *N*-linked [30,31,32,33], 6-C- [34] and 7-C-linked [32] have been reported to exhibit HDAC inhibitory activity, whereas several 2-C-linked derivatives inhibit matrix metalloproteinase-13 [35]. Finally, several 5-C linked QNZ-HMAs have been identified as dual PI3K/HDAC inhibitors [36].

3-OH-QNZ-HMA (**III**, Figure 1) represents the fussed assembly of two pharmacophores; QNZ and HMA. We have recently reported a high-yielding, green, microwave- assisted synthesis for the preparation of **III** and its alkyl and benzyl derivatives (**IV** and **V**, Figure 1) [37]. Free QNZ-HMA derivatives (**III**) have been shown to exhibit anticancer [38,39,40], antimicrobial and anti-inflammatory properties [41], environmental insecticidal activity [42] as well as hepatitis C virus NS5B polymerase inhibition [43]. In addition, evidence for their metal-chelating capacity has been provided for metal ions such as Fe(III), Co(II), Ni(II), Cu(II) and Zn(II) [44]. Interestingly, both the synthesis and biological evaluation of N-O-esters remain relatively limited. Nevertheless, several N-O-CH_2_-CO-O substituted QNZ-HMA esters have been shown to induce ferroptosis in colorectal cancer cells [45], and N-O-CH_2_-CO-NH substituted QNZ-HMA esters have exhibited anticancer activities against breast, cervical, liver and colon cancer cell lines [46].

Melanoma is a highly aggressive type of skin cancer that arises from the malignant transformation of melanocytes [47,48]. Although melanoma accounts for 2% of all skin cancer cases, it is responsible for approximately 80% of skin cancer-related deaths, and both its incidence and mortality have been increased worldwide [49,50]. Advanced-stage melanoma remains a major therapeutic challenge because it frequently exhibits resistance to conventional therapies [51]. Consequently, there is an urgent need to develop novel therapeutic approaches that can overcome or bypass resistance mechanisms.

Photodynamic therapy (PDT) is a promising, minimally invasive, and more precise treatment approach that minimizes damage to surrounding healthy tissue. PDT relies on the interaction of light of a specific wavelength with a photosensitizer and molecular oxygen, leading to the on-site activation of stimuli-sensitive chemical compounds [49,50]. Advances in the field are expected to contribute significantly to melanoma management while improving the patient’s quality of life.

Recently, quinazoline-based molecules have emerged as promising photosensitizers for PDT. Upon light irradiation, quinazoline derivatives can efficiently transfer energy to molecular oxygen, generating singlet oxygen [52]. The ability to combine intrinsic anticancer properties with light-triggered cytotoxicity positions quinazoline derivatives as potential dual-function agents, offering both targeted molecular inhibition and localized photodynamic action in cancer therapy.

Our previous work on the synthesis and photobiology of quinazolinones [37,53,54] and their metal complexes [55] as well as on the identification of novel DNA photocleavers and their applications [56,57,58,59,60,61] prompted the present investigation of the photobiological properties of compounds **III**, **IV** and **V** (Figure 1). It was initially anticipated that the free OH group in **III** would favor DNA binding through hydrogen-bonding interactions and, consequently, enhance DNA photocleavage. However, the results were rather surprising, as certain *O*-alkyl and *O*-benzyl derivatives (**IV** and **V)** exhibited significantly greater photocleavage of both DNA and cells than their corresponding free HMAs, highlighting hydroxamates as promising candidates for biological studies alongside their parent HMAs. In this work, the highly efficient singlet oxygen generation of these quinazolinone derivatives is also demonstrated, with yields that are significantly higher than those reported previously for related compounds.

## 2. Materials and Methods

### 2.1. Materials and Instrumentation

All commercially available reagent-grade chemicals and solvents were used without further purification. Trisodium citrate, NaCl, were purchased from Merck (Rahway, NJ, USA), EB from Sigma-Aldrich Co (Burlington, MA, USA), calf-thymus (CT) DNA from Sigma-Aldrich Co (St. Louis, MO, USA) and all solvents from Chemlab (Zedelgem, Belgium). DMSO for dissolving samples for the agarose gels electrophoresis was purchased from SERVA Electrophoresis GmbH (Heidelberg, Germany). DNA stock solution was prepared by dilution of CT DNA to buffer (containing 150 mM NaCl and 15 mM trisodium citrate at pH 7.0) followed by exhaustive stirring at 4 °C for 3 days and kept at 4 °C for no longer than a week. The stock solution of CT DNA gave a ratio of UV absorbance at 260 and 280 nm (A_260_/A_280_) of ~1.90, indicating that the DNA was sufficiently free of protein contamination [62]. The DNA concentration per nucleotide was determined by the UV absorbance at 260 nm after 1:20 dilution using ε = 6600 M^−1^ cm^−1^ [63]. Supercoiled plasmid pBluescript SK II has been synthesized and was tested not to contain nicked and/or linear strands. All samples containing pBluescript SK II were irradiated at pH 6.8 with Philips 2 × 9 W/10/2P UV-A lamps at 365 nm or white light OSRAM DULUX S BLUE. UV–vis spectra were recorded on a Hitachi U–2001 dual-beam spectrophotometer (Hitachi, Tokyo, Japan). Fluorescence spectra were recorded in solution on a Hitachi F-7000 fluorescence spectrophotometer (Hitachi, Tokyo, Japan). Viscosity experiments were carried out using an ALPHA L Fungilab rotational viscometer (Fungilab S.A., Barcelona, Spain) equipped with an 18 mL LCP spindle at 100 rpm. QNZ-HMAs, QNZ-HMA-OEt and QNZ-HMA-OBn **1**–**30** have been prepared under microwave irradiation-assisted syntheses by following a procedure described earlier [37].

### 2.2. Interaction with CT DNA

The interaction of the compounds with CT DNA was evaluated in vitro using their solutions in DMSO (1 mM) due to their low solubility in water. These studies were performed in the presence of aqueous buffer solutions, where mixing of each solution never exceeded 10% DMSO (*v*/*v*) in the final solution. Control experiments were undertaken to assess any effect of DMSO on the data and no changes were observed in the UV spectra of CT DNA solution. The interaction of the compounds with CT DNA was investigated with UV–vis spectroscopy, viscosity measurements, and via the evaluation of their EB–displacing ability studied with fluorescence emission spectroscopy. Detailed procedures and equations regarding the in vitro study of the interaction of the compounds with CT DNA are given in the Supporting Information File (Parts S1–S3).

### 2.3. DNA Cleavage and Photocleavage Experiments

Compounds **1**–**30** were individually incubated with supercoiled plasmid DNA pBluescript SK II at the desired concentration, in Eppendorf vials and/or were irradiated with UV-A or visible light (365 nm–18 W, or white light 400–700 nm–18 W) and in 10 cm distance under aerobic conditions at room temperature for 2 h. Conditions of the photobiological reaction and gel electrophoresis, quantification of DNA-cleaving activity and calculation of single-strand (ss)% and double-strand (ds)% damage protocols have been described previously [61]. All experiments were performed at least three times or otherwise noticed.

### 2.4. Singlet Oxygen Generation Measurement

The quantum yields of singlet oxygen generation were determined in DMF solution (3.0 mL, no oxygen bubbled) using the relative method with methylene blue (Sigma-Aldrich) and 1,3-diphenylisobenzofuran (DPBF) as a reference chemical quencher for singlet oxygen according to the previously described procedure [64]. Solutions of **8**, **18**, **28** or methylene blue in DMF and in the presence of DPBF were irradiated in a 1 cm path-length quartz cell (3 mL) with monochromatic light by a 150 W high-pressure Xe lamp (Optel, Opole, Poland) through a monochromator M250/1200/U (Optel, Opole, Poland). Light of wavelength adjusted to the maxima of band was used (absorbance of the sensitizers ~1). The concentration of DPBF was set at ~3 × 10^−5^ M to avoid chain reactions induced by DPBF in the presence of singlet oxygen [65]. The light intensity was set to 0.5 mW/cm^2^ (Radiometer RD 0.2/2 with TD probe (Optel, Opole, Poland)). The UV-vis spectra were recorded at the specific time points of irradiation using a Jasco-V770 spectrophotometer (Jasco Inc., Tokyo, Japan) to determine the rate of DPBF oxidation upon interaction with singlet oxygen generated from tested compounds and methylene blue. The Φ_Δ_ values were calculated following previously elaborated procedures [66] and presented as mean values from three measurements ± SD.

### 2.5. Molecular Docking Studies

Organic compounds were fully optimized at the B3LYP/6-31g* level of theory with the LanL2DZ basis set for iodine in case of compounds **6**, **16** and **26** as implemented in the Gaussian 09 [67] suite of programs (Revision B.01). The crystal data of the B-DNA dodecamer d(CGCGAATTCGCG)2 (PDB 1D:1BNA) were downloaded from the Protein Data Bank [68]. The docking analysis was performed using the AutoDock Vina program [69]. The DNA was adapted for docking by removing water molecules and polar hydrogens, and Gasteiger charges were added by Autodock 4.2 Tools (ADT) before performing docking calculations. Grid box with a size of 60 × 80 × 114 with 0.375 Å spacing was used to encompass the whole DNA. The rigid docking protocol and 100 runs of the Lamarckian genetic algorithm for searching ligand conformations were performed. PyMOL [70] was used for the representation of the docking results and interactions between DNA and compounds.

### 2.6. Cell Culture Experiments

A375 (CRL-1619, ATCC) melanoma cells and HaCaT (300493, Cytion) immortalized keratinocytes were cultured under aseptic conditions in high-glucose DMEM (LM-D1110, Biosera, Cholet, France) supplemented with 10% fetal bovine serum (FBS; P40-37500, PANBiotech, Aidenbach, Germany), 100 units/mL penicillin and 100 μg/mL streptomycin (DE17-602E, LONZA, Allendale, NJ, USA). COLO-800 (ACC 193, DSMZ) melanoma cells were cultured under aseptic condition in RPMI-1640 (LM-R1639, Biosera, France) supplemented with 10% FBS, 100 units/mL penicillin and 100 μg/mL streptomycin [54].

Cells were maintained at standard conditions (37 °C, 5% CO_2_) in a humidified incubator and were passaged when they reached 70–90% confluence. For cytotoxicity assays, 5000 cells per well were seeded in 96-well plates.

A UV-A lamp (365 nm) was placed 10 cm above the 96-well plates. Cells were first incubated with the tested compounds for 1 h. This incubation was followed by 1 h irradiation with UV-A. After irradiation, the compounds were removed, the culture medium was replaced with fresh medium, and a cytotoxicity assay was performed 24 h later. Untreated cells (UNT) and cells treated with 0.1% DMSO were included as controls to assess the phototoxic effect of UV-A irradiation and to account for any solvent-related cytotoxicity. A non-irradiated 96-well plate (DARK) was used as a control under the same conditions.

Cell viability was assessed using the Resazurin Cell Viability Assay (30025-2, Biotium, Fremont, CA, USA) according to the manufacturer’s instructions. Cells were incubated with 10% resazurin solution for 3 h, and fluorescence was then measured (λ_ex_ = 530/560 nm, λ_em_ = 590 nm) using an EnSpire Multimode Plate Reader (PerkinElmer, Shelton, CT, USA).

Statistical analysis was performed using GraphPad Prism software (version 8.4.2) (GraphPad Inc.). Data are presented as mean ± standard deviation (SD) from independent biological replicates. A two-way analysis of variance (ANOVA) was performed to evaluate the effects of treatment (photosensitizers and control). Multiple comparisons were carried out using Šidák correction to compare UV-A and DARK conditions within each treatment group. Adjusted *p*-values were reported and a value of *p* < 0.05 was considered statistically significant.

## 3. Results

### 3.1. Structures of HMAs and Derivatives

**Figure 2:**
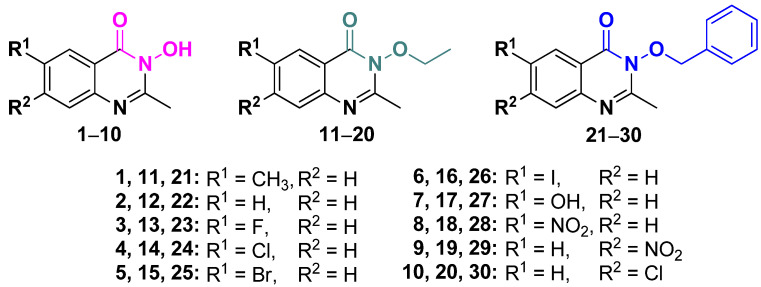
The three groups of the studied HMA derivatives; free HMAs (**1**–**10**), OEt hydroxamates (**11**–**20**) and OBn hydroxamates (**21**–**30**).

QNZ-HMAs, QNZ-HMA-OEt and QNZ-HMA-OBn **1**–**30** (Figure 2) were prepared under microwave-assisted conditions following a previously described procedure [37].

The three series comprise free HMAs (**1**–**10**), OEt hydroxamates (**11**–**20**) and OBn hydroxamates (**21**–**30**). The structure–activity relationships (SAR) within each series are discussed in terms of the electronic nature (electron-donating or electron-withdrawing) and positional effects of the aryl substituents on the 2-methylpyrimidinone ring, together with the presence and size of halogen atoms and the potential for hydrogen-bond formation. Finally, the activities of the three series are compared as a function of the N-substituent (OH, OEt or OBn) (Figure 2).

### 3.2. CT DNA Binding Studies of HMAs and Derivatives

The interaction of compounds **1**–**10**, **11**–**20** and **21**–**30** with CT DNA was evaluated in vitro by UV-vis spectroscopy, viscosity measurements and competitive ethidium bromide (EB) displacement experiments performed by fluorescence emission spectroscopy.

**Figure 3:**
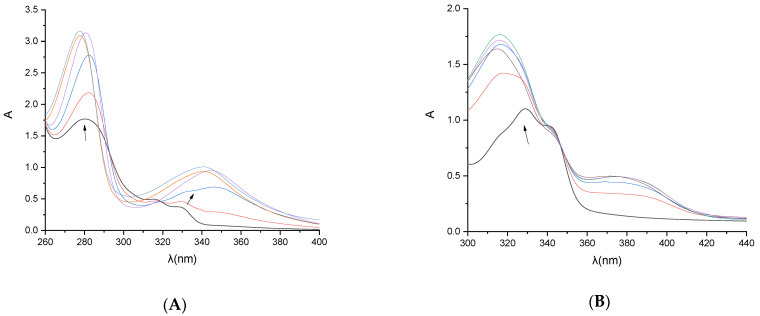
(**A**) UV-vis spectra of compound **5** (10^−4^ M) in DMSO in the presence of increasing amounts of CT DNA. The different colors show the spectra recorded for different [DNA]/[compound] ratios. The arrows show the changes upon increasing amounts of CT DNA. (**B**) UV-vis spectra of compound **17** (10^−4^ M) in DMSO in the presence of increasing amounts of CT DNA. The arrows show the changes upon increasing amounts of CT DNA. All experiments are shown in Supporting Information, Figures S2.1 and S2.2.

The structural changes induced by the interaction of CT DNA with the examined compounds have been investigated by means of UV–vis spectroscopy, which was exploited to measure DNA-binding constants (K_b_). In most cases, the bands in the UV–vis spectra of the compounds, exemplified by compounds **5** and **17** (Figure 3A and Figure 3B, respectively) showed hyperchromism or hypochromism accompanied by small red- or blue-shifts in the presence of increasing amounts of CT DNA (Table 1).

The K_b_ values of the compounds (Table 1) were obtained using the Wolfe–Shimer equation (Supporting Information, Part S1.1, Equation (S1)) [71] and plots of [DNA]/(ε_A_–ε_f_) versus [DNA] (Supplementary Information Part S2). Among the QNZ-HMAs, the Br-substituted compound **5** exhibited the highest DNA-binding constant (K_b_ = 6.27 (±0.49) × 10^4^ M^−1^). Within the QNZ-HMA-OEt series, compound **17** showed the strongest binding, with K_b_ = 7.89 (±0.39) × 10^4^ M^−1^. In the QNZ-HMA-OBn series, compound **23** reached K_b_ = (1.90 (±0.09) × 10^5^ M^−1^, the highest DNA-binding constant among all derivatives studied. It is notable that all compounds besides derivative **24** show relatively good binding constancies.

The above findings are consistent with DNA binding but insufficient on their own to unambiguously define the binding mode, necessitating the performance of other experiments such as DNA-viscosity measurements.

The method consists of the measuring of the DNA length before and after the incorporation of the compound under study. Any alterations in DNA structure following the addition of a compound under study were assessed through viscosity measurements (Supporting Information, Part S1.2), which are sensitive to changes in the relative DNA length (L/L_o_) and therefore provide insight into the binding mode. Generally, when a compound intercalates into DNA, the spacing between base pairs increases at the intercalation site to accommodate the inserted molecule. As a result, the overall DNA length increases, leading to an increase in viscosity which is often proportional to the strength of the interaction [72]. In contrast, non-classical binding modes (such as electrostatic interactions or groove binding) typically cause little to no elongation of the DNA helix and may even induce a slight decrease in viscosity [72].

**Figure 4:**
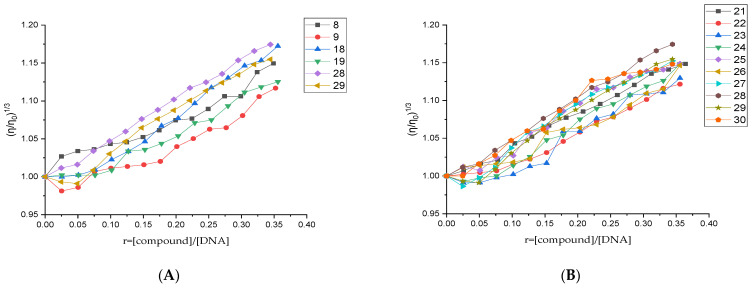
Relative viscosity (η/η_0_)^1/3^ of CT DNA (0.1 mM) in buffer solution (150 mM NaCl and 15 mM trisodium citrate at pH 7.0) in the presence of increasing amounts of (**A**) all nitro derivatives and (**B**) compounds **21**–**30**.

In this study, the viscosity of a CT DNA solution (0.1 mM) was measured upon addition of increasing amounts of the compounds up to *r* = 0.36 (Figure 4). At low compound-to-DNA ratios (*r* ≤ 0.1), the viscosity remained largely unchanged, indicating an external interaction, most likely groove binding. At higher ratios (*r* > 0.1), a gradual increase in DNA viscosity was observed, indicating the onset of intercalative binding. Among the nitro derivatives, compounds **18** and **28** exhibited the strongest effects, whereas among the three compounds’ groups, the third group of QNZ-HMA-OBn derivatives displayed the highest values, as shown in Figure 4, already indicating the compounds that are likely to exhibit cleavage and/or photocleavage activity.

Ethidium bromide (EB) displacement experiments were performed to further support the intercalative binding mode. EB is a fluorescent intercalator, forming an adduct characterized by a strong emission band at 592–593 nm when excited at 540 nm [64]. When a compound capable of intercalating into DNA as effectively as or more strongly than EB is added to an EB–DNA solution, alterations in the EB–DNA emission band can occur. These changes are commonly monitored to evaluate the compounds’ ability to compete with EB for the DNA intercalation site [73].

**Figure 5:**
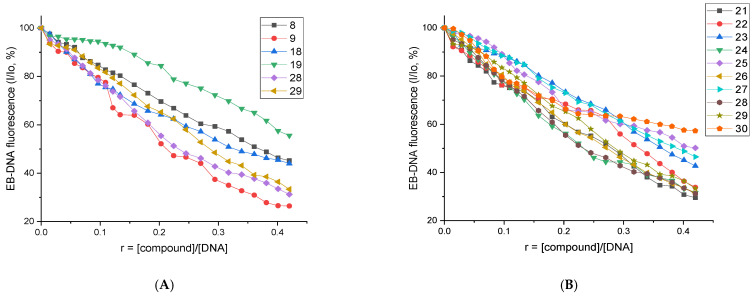
Plot of EB–DNA relative fluorescence emission intensity at λ_emission_ = 592 nm (%) versus *r* (*r* = [compound]/[DNA]) in the presence of (**A**) all nitro-derivatives and (**B**) compounds **21**–**30**.

In this study, the fluorescence emission spectra of EB–DNA pretreated for 1 h ([EB] = 20 μM, [DNA] = 26 μM) were recorded in the presence of increasing concentrations of the compounds. A marked reduction in the EB–DNA emission band at 592 nm, up to 73.6% for compound **9** (Table 2, Supporting Information Figure S3.1) was recorded, indicating efficient displacement of EB from its binding site. This behavior is consistent with an intercalative interaction of the compounds with CT DNA. Among all the compounds, derivatives **6**, **9**, **21**, **22**, **24**, **26**, **28**, and **29** were the ones that exhibited a quenching (∆I/Io) percentage higher than 65%. The majority of them belong to the QNZ-HMA-OBn group. The plots of EB-DNA I/I_o_ (%) versus *r* (*r* = [compound]/[DNA]) in the presence of (**A**) all nitro-derivatives and (**B**) compounds **21**–**30** are depicted in Figure 5. Among all NO_2_-QNZ-HMA, derivatives **9**, **28** and **29** are the most intercalative compounds, whereas among QNZ-HMA-OBn, derivatives **21**, **22**, **24**, **26**, **28**, and **29** are the most potent. This observation suggests that the OBn group plays a significant role in the interaction of the corresponding derivatives with DNA, a hypothesis that will be further examined through additional experiments presented below.

The Stern–Volmer constants (K_SV_) for the compounds (Table 2) were obtained from Stern–Volmer plots (Supporting Information, Part S1.3, Equation (S2) and Part S3). The K_SV_ values are relatively high, with compounds **9**, **24** and **28** showing the highest constants (8.90 × 10^4^, 7.10 × 10^4^ and 7.19 × 10^4^ M^−1^, respectively), indicating strong binding to DNA. Additionally, the EB–DNA quenching rate constants (K_q_) (Table 2) were calculated using Equation (S3) (Supporting Information, Part S1.3), assuming a fluorescence lifetime (τ_0_) of 23 ns [74]. The K_q_ values exceeded 10^10^ M^−1^s^−1^ supporting a static quenching mechanism [73], in which the compounds interact directly with the fluorophore within the EB–DNA adduct. Again, although all derivatives exhibit appreciable displacement of EB from DNA, the OBn–HMA derivatives show the highest activity (compounds **24** and **28**), including compound **28**, which features a NO_2_-substituted quinazolinone scaffold.

### 3.3. DNA Interactions of HMAs and Derivatives with Plasmid DNA

#### 3.3.1. DNA Cleavage Experiments

The UV-vis spectra of compounds **1**–**30** in DMSO solutions are provided in the Supporting Information (Part S4). All compounds were initially dissolved in DMSO, and the resulting solutions were found to be stable when stored at 4 °C. For DNA cleavage studies, each compound was incubated individually with supercoiled plasmid DNA pBluescript SK II (500 ng) in buffer, maintaining the final DMSO concentration below 10% (*v*/*v*) to minimize solvent effects.

Dark-condition experiments were performed at a concentration of 100 µM for the most photoactive derivatives (vide infra) **8**, **9**, **18**, **19**, **28** and **29**, which were incubated with supercoiled plasmid DNA for 150 min. Under these conditions, the compounds exhibited only low DNA-cleaving activity in the absence of light, corresponding to 5–28% single-strand cleavage (ss, Form II, Supporting Information Figure S5.1). All agarose gel images from cleavage and photocleavage experiments are presented in Supporting Information, Part S5.

#### 3.3.2. DNA Photocleavage Experiments

For photocleavage assays, all compounds (100 μM) were mixed with supercoiled plasmid DNA pBluescript SK II, incubated for 30 min, and then irradiated for 120 min at a distance of 10 cm under aerobic conditions at room-temperature, either at 365 nm (UV-A) or with visible light (400–700 nm). The majority of the compounds showed photosensitization and induced DNA photocleavage, as evidenced on agarose gels by conversion of supercoiled plasmid DNA (Form I) to nicked DNA (ss, Form II), and in some cases, to linear DNA (double-strand (dd) cleavage (Form III).

**Figure 6:**
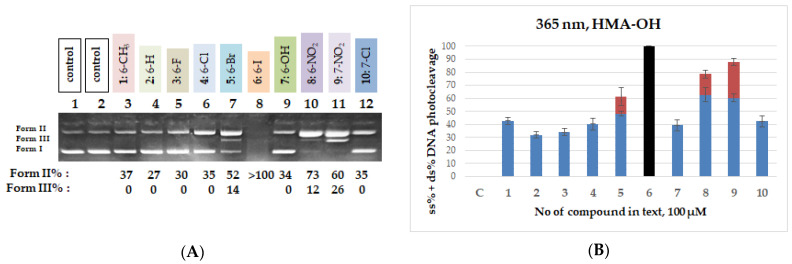
(**A**) DNA agarose gel picture (single experiment) for compounds **1**–**10** under 365 nm irradiation for 120 min, at a concentration of 100 μM. Calculations of Form II and Form III % are shown below the picture. (**B**) Plots of DNA photocleavage of compounds **1**–**10** under 365 nm irradiation for 120 min for the triplicate: black column: >100% cleavage, blue column: ss% cleavage; red column: ds% cleavage (for all gel pictures, see Supporting Information, Figure S5.2).

Upon 365 nm irradiation, all QNZ-HMAs **1**–**10** demonstrated DNA-cleaving activity, yielding ss in proportions ranging from 27% to >100%. Compounds **1**–**4**, **7** and **10** exhibited the lowest cleavage levels (ss% = 27–37%). Among the halogenated derivatives, the chloro-substituted analogs (**3**, **4**, **10**) displayed low-to-moderate activity, whereas the bromo derivative **5** generated both single-strand and double-strand breaks (52% Form II and 14% Form III). The iodo derivative **6** completely degraded the plasmid DNA under these conditions, leaving no detectable band, indicating that the tested concentration was too high for quantitative evaluation. In contrast, the nitro derivatives **8** and **9** yielded high levels of DNA damage, with Form II at 73% and 60% and Form III at 12% and 26%, respectively. A representative gel and quantitative data for the QNZ-HMAs (**1**–**10**) are shown in Figure 6; full datasets are provided in Supporting Information Figure S5.2.

**Figure 7:**
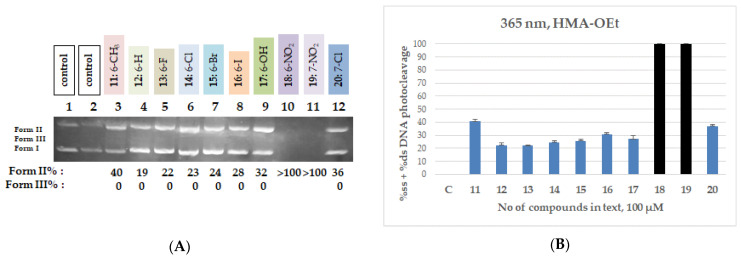
(**A**) DNA agarose gel picture (single experiment) for compounds **11**–**20** under 365 nm irradiation for 120 min, at a concentration of 100 μM. Calculations of Form II and Form III % are shown below the picture. (**B**) Plots of DNA photocleavage of compounds **11**–**20** under 365 nm irradiation for 120 min for the triplicate: black column: >100% cleavage, blue column: ss% cleavage (for all gel pictures, see Supporting Information, Figure S5.3).

Within the QNZ-HMA-OEt series (**11**–**20**), only the two nitro derivatives **18** and **19** exhibited noteworthy DNA photocleavage, each achieving >100% DNA fragmentation under UV-A irradiation, whereas the remaining compounds showed moderate to low activity. The halogenated derivatives produced single-strand cleavage in the range of 22% to 36%. The limited activity of the rest of the compounds, including the iodo derivative **16**, likely reflects weak UV absorption above ~340 nm in comparison to the QNZ-HMA-OH. A representative gel and quantitative data for the QNZ-HMA-OEt (**11**–**20**) are shown in Figure 7; full datasets are provided in Supporting Information Figure S5.3.

**Figure 8:**
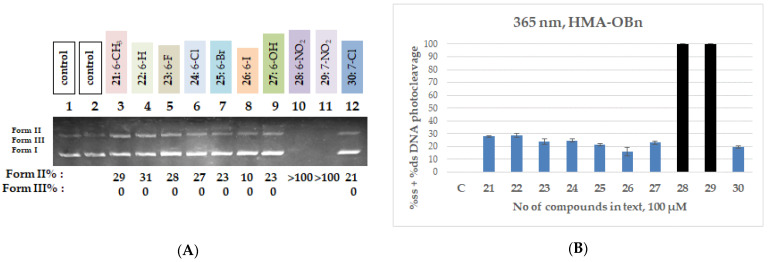
(**A**) DNA agarose gel picture (single experiment) for compounds **21**–**30** under 365 nm irradiation for 120 min, at a concentration of 100 μM. Calculations of Form II and Form III % are shown below the picture. (**B**) Plots of DNA photocleavage of compounds **21**–**30** under 365 nm irradiation for 120 min for the triplicate: black column: >100% cleavage, blue column: ss% cleavage (for all gel pictures, see Supporting Information, Figure S5.4).

A similar pattern is observed in the third group of compounds (**21**–**30**), where again the only compounds which demonstrated significant activity were the two nitro derivatives (**28** and **29**), which, at the tested concentration, also cleaved the entire amount of DNA, in line with the CT DNA binding and EB displacement data. More specifically, the halogenated derivatives yield Form II at levels ranging from 10% to 28% in the case of the QNZ-HMA-OBn compounds, which most probably corresponds to cleavage and not photocleavage. A representative gel and quantitative data for the QNZ-HMA-OEt (**21**–**30**) are shown in Figure 8; full datasets are provided in Figure S5.4.

**Figure 9:**
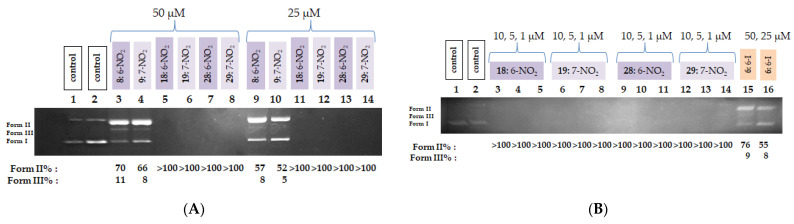
(**A**) DNA agarose gel picture (single experiment) for compounds **8**, **9**, **18**, **19**, **28** and **29** under 365 nm irradiation for 120 min, at concentrations of 50 and 25μM. Calculations of Form II and Form III% are shown below the picture. (**B**) DNA agarose gel picture (single experiment) for compounds **18**, **19**, **28**, **29** and **6** under 365 nm irradiation for 120 min, at concentrations of 10, 5 and 1 μM. Calculations of Form II and Form III % are shown below the picture.

To elucidate the mechanism of DNA photocleavage, selected highly active derivatives (**6**, **8**, **9**, **18**, **19**, **28** and **29**) were subjected to additional assays under modified conditions. The experiments were repeated for all nitro-substituted compounds, as well as for compound **6**, this time at lower concentrations. Reducing the concentration to 50 μM revealed the limits of activity for compounds **6**, **8**, and **9**. Specifically, the two nitro derivatives (**8** and **9**) generated ss at levels of 70% and 66%, respectively, while ds were produced only in minor amounts (11% and 8%, respectively). Compound **6** yielded 76% Form II and 9% Form III under the same conditions, Figure 9A.

Further decreasing the concentration to 25 μM resulted, as expected, in a continued reduction in activity for compounds **8**, **9**, and **6**, with the Form II levels dropping to 57%, 52%, and 55%, respectively. At these same concentrations (50 and 25 μM), the nitro derivatives (compounds **18**, **19**, **28**, and **29**) cleaved the entire amount of DNA, necessitating an additional reduction in concentration for these compounds. Thus, DNA photocleavage experiments were conducted at 10, 5, and 1 μM. Even at these reduced concentrations, the four nitro derivatives exhibited remarkably high activity, producing DNA cleavage percentages exceeding 100%. These results left no alternative but to further lower the concentrations, this time into the nanomolar range, Figure 9B.

**Figure 10:**
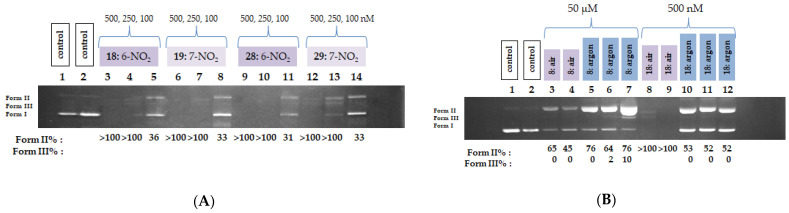
(**A**) DNA agarose gel picture (single experiment) for compounds **18**, **19**, **28** and **29** under 365 nm irradiation for 120 min, at concentrations of 500, 250 and 100 nM. Calculations of Form II and Form III % are shown below the picture. (**B**) DNA agarose gel picture (single experiment) of mechanistic studies for compounds **8** and **18**, at concentrations of 50 μM and 500 nM respectively, under aerobic and anaerobic conditions.

Compounds **18**, **19**, **28**, and **29** were irradiated at 365 nm for 2 h at concentrations of 500, 250, and 100 nM. At 500 and 250 nM, all four compounds cleaved the entire amount of DNA. The limits of their activity became apparent only when the concentration was decreased to 100 nM, at which point they produced Form II at levels slightly above 30%, as illustrated in Figure 10A. The photoactivity of certain QNZ derivatives has previously been demonstrated by our group, primarily involving 6-Br, 6-OH, 6-NO_2_, and 7-NO_2_ substituted QNZs. Among these, 6-nitroquinazolinone exhibited DNA photocleavage activity under UV-A irradiation at concentrations up to 10 μM, while its activity against A375 melanoma cells reached 50 μM [54]. Modification of the substituents at position 3 of the QNZ scaffold, such as replacement of the hydrogen atom with an NH_2_ group (3-amino-2-methyl-6-nitroquinazolinone) or even with an amide group, significantly enhanced activity, reducing the concentration required for 50% plasmid DNA photocleavage to as low as 1–5 μM. Molecular docking studies for these latter compounds indicated a high binding affinity, with calculated energies of approximately −10 kcal/mol for the amide derivatives [53]. Compared to these previously reported QNZ derivatives, the results presented herein demonstrate a substantial improvement, as the concentration required to achieve 50% photocleavage of plasmid DNA has been reduced to the nanomolar range. In this study, the presence of the NO_2_ group once again appears to be crucial. Furthermore, the incorporation of OEt, and particularly the OBn group, significantly enhances activity. These findings are consistent with the results obtained from the binding studies.

To gain insight into the mechanism of action of the compounds, mechanistic studies were conducted in which DNA photocleavage experiments were repeated for compounds **8** and **18** at concentrations of 50 μM and 500 nM, respectively, both in the presence and absence of oxygen. The results of the mechanistic studies indicated that, in the case of compound **18**, a significant decrease in its biological activity was observed in the presence of argon, whereas for compound **8,** no similarly distinct effects were detected, as illustrated in Figure 10B.

**Figure 11:**
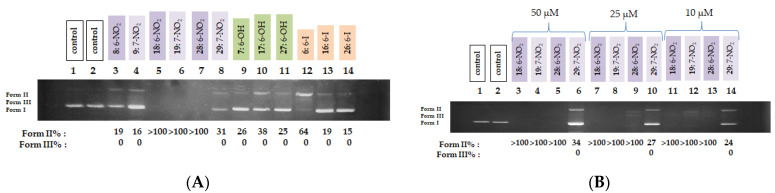
(**A**) DNA agarose gel picture (single experiment) for nitro, hydroxyl and iodo derivatives under visible light irradiation for 120 min, at a concentration of 100 μM. Calculations of Form II and Form III % are shown below the picture. (**B**) DNA agarose gel picture (single experiment) for compounds **18**, **19**, **28** and **29** under visible light irradiation for 120 min, at concentrations of 50, 25 and 10 μM. Calculations of Form II and Form III % are shown below the picture.

The DNA photocleaving activity of nitro derivatives (compounds **8**, **9**, **18**, **19**, **28**, and **29**), along with hydroxy (**7**, **17**, and **27**) and iodo (**6**, **16**, and **26**) derivatives, was evaluated under visible-light irradiation. Hydroxy- and iodo derivatives generally exhibited low DNA-cleaving activity, with Form II percentages ranging from 15 to 26%. The notable exception was compound **6**, which displayed a significantly higher Form II percentage of 64% at 100 μM, as illustrated in Figure 11A. In contrast, nitro derivatives **18**, **19**, **28**, and **29** demonstrated particularly strong DNA-cleaving activity, warranting further investigation at lower concentrations. At 50, 25, and 10 μM, three of these compounds (**18**, **19**, and **28**) induced DNA cleavage ~100%, indicating a robust effect. Compound **29** showed more moderate activity, with Form II percentages of 34, 27, and 24% at the same concentrations, respectively (Figure 11B).

### 3.4. Singlet Oxygen Generation

**Figure 12:**
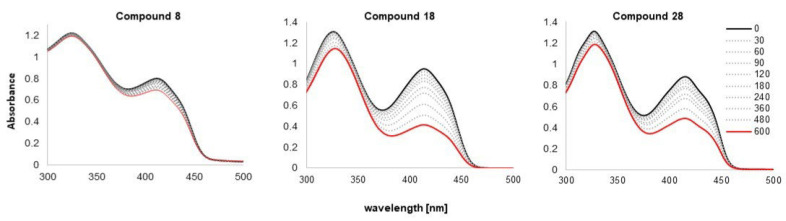
The time-dependent UV-Vis spectra for DPBF upon irradiation with **8**, **18**, **28** in DMF.

The potential photosensitizing activity of the obtained compounds **8**, **18** and **28** was evaluated by measuring their individual ability to generate singlet oxygen resulting from the interaction between the activated photosensitizer and triplet oxygen. 1,3-Diphenylisobenzofuran (DPBF) was used as a chemical quencher, which undergoes a cycloaddition reaction with singlet oxygen to produce endoperoxide [75]. Solutions containing **8**, **18**, **28** or methylene blue in a mixture with DPBF in DMF were irradiated with monochromatic light at the wavelengths corresponding to the Q-band maxima of their monomeric form. The kinetics of DPBF decomposition by photogenerated singlet oxygen was studied by monitoring decrease in the absorbance at 417 nm, and they were used to calculate the singlet oxygen generation yields (Φ_Δ_); results are presented in Table 3. The changes in the UV-vis spectra during measurements are shown in Figure 12, whereas first-order plots of DPBF degradation by singlet oxygen are presented in Figure S6.

The singlet oxygen generation efficiencies of the studied compounds show pronounced differences. Compound **8** exhibited a relatively low singlet oxygen quantum yield (Φ**_Δ_ =** 0.157), indicating limited photosensitizing capability. In contrast, compound **18** demonstrated a markedly higher efficiency with a quantum yield of 0.845, while compound **28** also showed strong singlet oxygen production (Φ_Δ_ = 0.795).

Compared to compound **8**, derivatives **18** and **28** generated approximately 5-fold higher amounts of singlet oxygen, suggesting that structural modifications of the OH group at those nitro derivatives significantly enhanced intersystem crossing (ISC) and triplet-state formation. Nitro groups are strong electron-withdrawing moieties that significantly influence the photophysical behavior of aromatic systems. In the case of our quinazolinones, the existence of the ether HMAs likely promotes efficient ISC by enhancing spin–orbit coupling and facilitating population of the triplet excited state, which is essential for effective singlet oxygen generation [76]. The slightly lower singlet oxygen quantum yield of compound **28** relative to compound **18** may reflect differences in ether substitution (Bn or Et, respectively), electronic conjugation, or steric effects that subtly modulate excited-state dynamics. Nevertheless, both nitro derivatives demonstrated singlet oxygen generation efficiencies comparable to established photosensitizers used in PDT, underscoring the beneficial role of both nitro at QNZ as well as ether substitution at HMA counterparts in enhancing photodynamic performance.

### 3.5. Molecular Docking “In Silico” Calculations of HMAs and Derivatives **1**–**30**

Molecular docking simulations were carried out for derivatives **1**–**30** using the AutoDock Vina software. The aim was to determine their polar interactions and estimate the corresponding DNA binding energies. Table 4 summarizes the calculated binding energies along with the identified DNA base contacts. The molecular docking experiments revealed that all compounds (**1**–**30**) interact with DNA at a defined binding region, with the majority forming polar contacts with a consistent triad of bases: DG16, DG10, and DC11. Specifically, all QNZ-HMA derivatives engage this DG16–DG10–DC11 triad, with the sole exception of compound **8**, which interacts with DC15 in place of DC11. Moreover, compounds **5**, **7**, and **9** display additional interactions beyond this primary triad: compound **5** forms supplementary contacts with DC9, compound **7** with DC15, and compound **9** with DA17 and DG12, thereby influencing their respective binding energies. Across all compounds in this group, the quinazolinone carbonyl and the free hydroxyl group at position 3 constitute key contributors to the stabilization of the binding pose. The highest binding affinity observed within this series (−7.0 kcal/mol) corresponds to the nitro derivatives (compounds **8** and **9**).

In the second series of compounds (QNZ-HMA-OEt, **11**–**20**), the quinazolinone carbonyl group once again participates in polar contact formation across all derivatives. However, the oxygen atom at position 3, now incorporated into an ethoxy substituent rather than present as a free hydroxyl, does not appear to engage in interactions with the DNA molecule. The DG16–DG10–DC11 base triad is again implicated in nearly all interactions, with the exception of compound **11**, which displays no detectable interaction with DC11. Compounds **17**, **18**, and **19** exhibit additional interactions with bases such as DG14 (for compounds **17** and **18**). Notably, compound **19** forms the greatest number of polar contacts, engaging bases DA17 and DG12 in addition to the primary triad. Within this group, the highest binding affinity (−7.4 kcal/mol) is observed for compound **18**.

**Figure 13:**
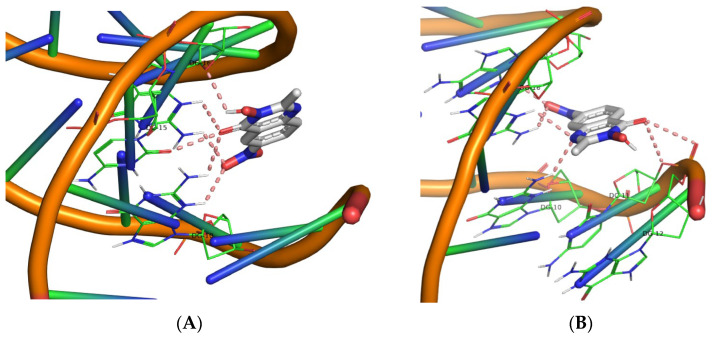
3D structures of the polar contacts of (**A**) compound **8** and (**B**) compound **9**. (For all pictures of compounds **1**–**10**, see Figure S7).

**Figure 14:**
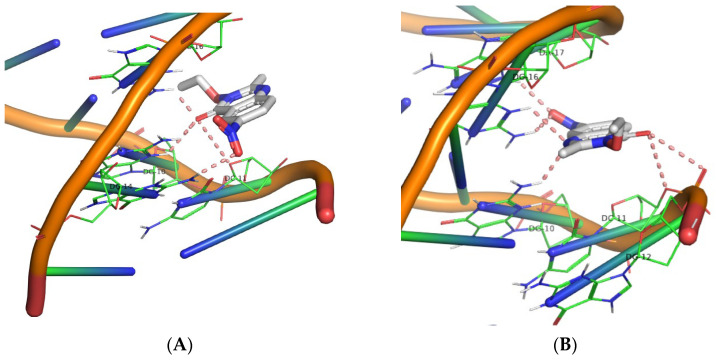
3D structures of the polar contacts of (**A**) compound **18** and (**B**) compound **19**. (For all pictures of compounds **11**–**20,** see **Figure S7**).

**Figure 15:**
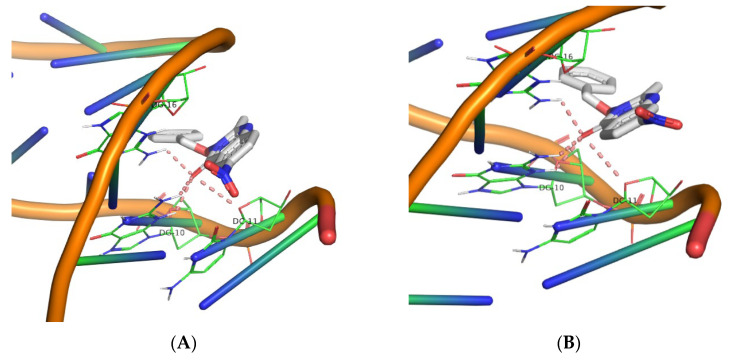
3D structures of the polar contacts of (**A**) compound **28** and (**B**) compound **29**. (For all pictures of compounds **21**–**30**, see **Figure S7**).

In the third compound series (QNZ-HMA-OBn, **21**–**30**), it is noteworthy that the oxygen atom at position 3, now substituted with a benzyloxy group, again fails to participate in interactions with the DNA molecule. All compounds in this series interact with the DG16–DG10–DC11 triad, with the exception of compound **27**, which does not form polar contacts with DG16. The highest binding affinity in this set and the highest among all 30 compounds corresponds to compound **28**, with a binding energy of −8.8 kcal/mol (all 3D images are found in Part S7). It is evident from the calculations that the OBn group enhances E-binding compared to the other two categories. Although the calculated binding energy of the most active compound (**28**, OBn) is lower than that reported for structurally related compounds bearing an NH–CO–Ar substituent (−8.8 kcal/mol vs. ~−10 kcal/mol [53], respectively), compound **28** demonstrated superior DNA photocleavage activity relative to the literature compounds (nanomolar vs. 1–5 μM range). The 3D structures illustrating the polar contacts of the nitro derivatives are presented in Figure 13, Figure 14 and Figure 15, while those of all compounds are included in the Supporting Information (Figure S7).

### 3.6. Cell Culture Experiments of Selective HMAs with Melanoma Cell Lines

Skin cancer cell lines are a commonly used model in PDT studies, especially in cases where photosensitizers are activated by UV irradiation, which has a relatively low tissue penetration depth. The phototoxic effects of selected potent DNA photocleavers (**6**, **8**, **9**, **18**, **19**, **28** and **29**) were evaluated in two human melanoma cell lines (A375 and COLO-800) and in immortalized keratinocytes (HaCaT).

Cells were incubated with 50 μM of **6**, **8**, **9**, **18** and **19**, and 10 μM of compounds **28** and **29**. The lower concentration selected for **28** and **29** is due to the presence of a benzyl group in their structure, which increases their hydrophobicity and decreases their solubility in cell culture medium.

#### 3.6.1. A375 Cell Line

**Figure 16:**
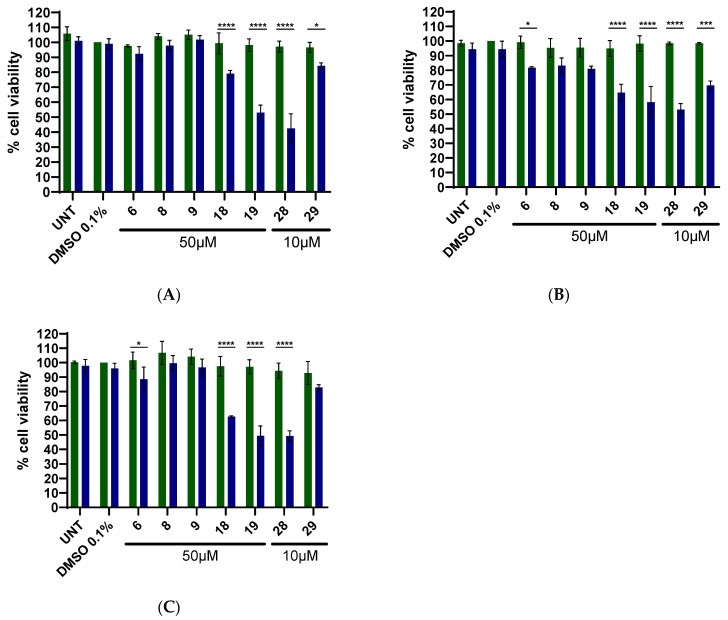
(**A**) Cytotoxic effect of compounds **6**, **8**, **9**, **18**, **19**, **28** and **29** on the A375 cell line for 60 min. DMSO 0.1% was used as a control; * *p* = 0.0114, **** *p* < 0.001; (**B**) Cytotoxic effect of compounds **6**, **8**, **9**, **18**, **19**, **28** and **29** on the COLO-800 cell line for 15 min. DMSO 0.1% was used as a control; * *p* = 0.0182, *** *p* = 0.001, **** *p* < 0.001; (**C**) Cytotoxic effect of compounds **6**, **8**, **9**, **18**, **19**, **28** and **29** on the HaCaT cell line for 60 min. DMSO 0.1% was used as a control; * *p* = 0.0447, **** *p* < 0.001; UNT: Untreated cells. The green bars refer to studies in dark, and the blue bars refer to studies after UVA irradiation.

A375 is a highly aggressive melanoma cell line. Compounds were tested after 1 h incubation followed by 60 min UV-A irradiation (365 nm), and cell viability was measured 24 h later using the resazurin assay. DMSO (0.1%) and untreated cells (UNT) were included as controls, and “DARK” plates were maintained in parallel without irradiation. Compounds **6**, **8** and **9** either irradiated or kept in the dark, showed little to no effect, on A375 viability. In contrast, compounds **18** and **29** reduced cell viability by 20% and 15%, respectively. Derivatives **19** and **28** demonstrated significant light-dependent cytotoxicity. Specifically, treatment with **19** reduced the viable cell population to slightly more than half of the control, while **28** eradicated about 58% of the cancer cells (Figure 16A). Notably, **28** exhibited significant cytotoxic activity at a concentration five-fold lower than that of **19**. Reported results on A375 melanoma cells for 6-NO_2_-quinazolinone (50 μΜ concentration) [54] show that at least derivative **28** exhibits better photo-cytotoxicity.

#### 3.6.2. COLO-800 Cell Line

COLO-800 is melanoma cell line with high metastatic potential and strong tumorigenicity in xenograft models. Control experiments with UV-A irradiation alone showed that COLO-800 cells are more susceptible to UV-A-induced cytotoxicity than A375 and HaCaT cells (Supplementary Figure S8A). To minimize UV-A cytotoxicity in the absence of compounds, a shorter irradiation time (15 min) was therefore used for COLO-800 (Supplementary Figure S8B).

Under these conditions, compounds **6**, **8** and **9** reduced cell viability by approximately 20%, while compounds **18** and **29** reduced viability by about 35% and 30%, respectively. Compounds **19** and **28** eliminated roughly 50% of the cell population upon UV-A activation (Figure 16B). Notably, **28** achieved this effect at a substantially lower concentration than **19**.

#### 3.6.3. HaCaT Cell Line

HaCaT is a spontaneously immortalized keratinocyte cell line that serves as a model for non-malignant skin cells. Under the same irradiation protocol used for A375 (60 min UV-A), compounds **6**, **8** and **9** did not show measurable cytotoxic effects on HaCaT cells, while **29** reduced cell viability by approximately 20%. Compound **18** eliminated about 40% of the cell population after UV-A activation and **19** exhibited even higher cytotoxicity, with only about 50% of cells surviving (Figure 16C). Remarkably, compound **28** at 10 μM exhibited cytotoxicity comparable to that of **19**, which was tested at 50 μM, indicating a favorable phototherapeutic window for **28**.

## 4. Conclusions

A set of three groups of QNZ-HMAs have been studied for their efficiency to photocleave DNA. The study was supported by DNA binding experiments that involved UV-vis absorption of the compounds in conjugation with CT DNA, viscosity measurements and fluorescent spectroscopy with EtBr. All compounds were found to have sufficient binding with DNA most probably via intercalation. Nitro derivatives **18** and **28** exhibited the strongest effects in viscosity experiments, whereas derivatives **6**, **9**, **21**, **22**, **24**, **26**, **28**, and **29** were the ones that exhibited a ∆I/Io percentage greater than 65% at the EB exchange experiments. The majority of the compounds belong to the QNZ-HMA-OBn group. Concerning the DNA photocleavage, agarose gel experiments indicated compounds **6**, **8**, **9**, **18**, **19**, **28** and **29** to be the most potent ones under UVA and visible light. Under UVA irradiation **6**, **8** and **9** showed potency at 25 μΜ, whereas the two nitro-QNZ-HMA-OEt and -OBn (**18**, **19**, **28** and **29**, respectively) were found to be extremely reactive in concentrations ranging from 250 to 100 nM. Based on the mechanistic studies, oxygen was found to be essential for their activity. This finding was verified by singlet oxygen generation measurements. Indeed, high singlet oxygen generation efficiencies were observed for the nitro derivatives **18** and **28,** a fact that places them among the highly efficient photosensitizers, comparable to or exceeding many commonly used PDT reference compounds. Molecular docking studies were also in consistence with DNA binding and singlet oxygen generation experiments. All nitro derivatives **8** and **9**, **18** and **19**, **28** and **29** and OH-QNZs **17** and **27** showed better interaction compared to the rest of the derivatives within their individual group. What is however noticeable is that all OBn derivatives **21**–**30** were calculated as having better interactions compared to the other two groups. Derivative **28** was the best overall binder.

Finally, cell culture experiments in two malignant melanoma cell lines (A375 and COLO-800) and in HaCaT keratinocytes identified three nitro derivatives (**18**, **19** and **28**) that, at concentrations up to 10 μM, reduced cell viability by approximately 50% upon UV-A activation.

Taken together, the DNA binding, photocleavage, singlet oxygen generation and phototoxicity profiles highlight NO_2_-substituted QNZ-HMA-OR derivatives, particularly **18**, **19** and **28**, as promising candidates for further development as photodynamic therapy agents.

## Figures and Tables

**Table 1:** Spectral features of the UV-vis spectra of compounds **1**–**30** upon addition of CT DNA. UV-band (λ_max_, in nm) (percentage of observed hyper-/hypochromism (ΔA/A_0_, in %), blue-/red-shift of the λ_max_ (Δλ, nm)) and the corresponding DNA-binding constants (K_b_, in M^−1^).

No.	Band (nm) (ΔA/A_o_(%) ^a^, Δλ(nm) ^b^)	K_b_ (M^−1^)
**1**	273 (>+50 ^c^, 0); 312 (−5, elim) ^c^; 325 (>+50, +10)	1.80 (±0.33) × 10^3^
**2**	271 (>+50, 0); 305 (−8, elim); 318 (>+50, +12)	4.95 (±0.29) × 10^4^
**3**	273 (>+50, 0); 314 (−15, elim); 326 (>+50, +12)	2.68 (±0.68) × 10^4^
**4**	280 (>+50, 0); 316 (−10, +10); 329 (>+50, +10)	1.45 (±0.14) × 10^4^
**5**	280 (>+50, 0); 316 (>+50, +10); 327 (>+50, +10)	6.27 (±0.49) × 10^4^
**6**	283 (+18, −2); 317 (+20, +3); 330 (+40, 0)	6.21 (±0.08) × 10^3^
**7**	273 (+20, 0); 330 (+3, 0); 346 (+10, +1)	1.07 (±0.29) × 10^4^
**8**	338 (>+50, −26)	2.07 (±0.02) × 10^3^
**9**	280 (sh) ^c^ (−25, 0); 325 (>+50, −5)	4.84 (±0.30) × 10^4^
**10**	281 (>+50, 0); 307 (−30, +10); 320 (>+50, +20)	1.46 (±0.08) × 10^4^
**11**	270 (−2, 0); 311 (+8, 0); 323 (+10, 0)	2.06 (±0.43) × 10^4^
**12**	268 (−3, +2); 305 (−5, 0); 316 (−5, 0)	9.52 (±0.45) × 10^3^
**13**	265 (−5, 0); 275 (−2, 0); 313 (−2, 0); 325 (−2, 0)	2.09 (±0.30) × 10^4^
**14**	274 (−3, 0); 315 (−2, 0); 328 (−2, 0)	9.60 (±0.08) × 10^3^
**15**	276 (−5 0); 315 (−2, 0); 328 (−2, 0)	1.01 (±0.33) × 10^4^
**16**	280 (−15, −2); 317 (−20, +2); 331 (−25, 0)	1.82 (±0.12) × 10^3^
**17**	275 (>+50, 0); 314 (>+50, −5); 329 (>+50, elim); 343 (−8, 0)	7.89 (±0.39) × 10^4^
**18**	332 (−3, 0)	9.46 (±0.55) × 10^3^
**19**	348 (+3, 0)	1.63 (±0.18) × 10^3^
**20**	274 (−5, +2); −306 (−10, 0); 318 (−5, 0)	4.93 (±0.50) × 10^3^
**21**	270 (+3, 0); 311 (−5 0); 324 (−5, 0)	2.63 (±0.45) × 10^4^
**22**	269 (+10, 0); 305 (+15, 0); 316 (+15, 0)	4.06 (±0.75) × 10^3^
**23**	266 (−15, 0); 312 (−20, 0); 324 (−20, 0)	1.90 (±0.09) × 10^5^
**24**	273 (−3, 0); 315 (−10, 0); 328 (−10, 0)	1.46 (±0.06) × 10^2^
**25**	275 (−10, 0); 315 (+10, 0); 329 (+14, 0)	2.56 (±0.78) × 10^4^
**26**	281 (−3, 0); 318 (+15, 0); 331 (+20, 0)	3.38 (±0.43) × 10^3^
**27**	273 (−10, +1); 314 (+35, −2); 329 (+2, elim); 343 (−3, 0)	3.12 (±0.37) × 10^4^
**28**	329 (−10, 0)	2.00 (±0.67) × 10^4^
**29**	350 (+4, 0)	2.92 (±0.48) × 10^3^
**30**	272 (+5, 0); 305 (−5, 0); 317 (−5, 0)	6.87 (±0.55) × 10^3^

^a^ “+” denotes hyperchromism, “−” denotes hypochromism; ^b^ “+” denotes red-shift, “−” denotes blue-shift; ^c^ “>+50” denotes very high hyperchromism, “elim” = eliminated, “sh” = shoulder.

**Table 2:** Data of the EB-DNA competitive studies of compounds **1**–**30**. Percentage of EB-DNA fluorescence quenching (ΔI/I_o_, in %), EB-DNA Stern–Volmer constants (K_SV_, in M^−1^) and EB-DNA quenching constants (K_q_, in M^−1^s^−1^) for compounds **1**–**30**.

No. of Compound	(∆I/I_o_, %)	K_SV_ (M^−1^)	K_q_, M^−1^ s^−1^
**1**	60.1	3.79 (±0.08) × 10^4^	1.65 (±0.04) × 10^12^
**2**	60.6	3.16 (±0.08) × 10^4^	1.37 (±0.04) × 10^12^
**3**	54.2	3.72 (±0.09) × 10^4^	1.62 (±0.04) × 10^12^
**4**	59.6	3.22 (±0.12) × 10^4^	1.40 (±0.05) × 10^12^
**5**	62.9	3.50 (±0.08) × 10^4^	1.52 (±0.03) × 10^12^
**6**	66.4	3.38 (±0.11) × 10^4^	1.47 (±0.05) × 10^12^
**7**	57.4	2.45 (±0.06) × 10^4^	1.07 (±0.03) × 10^12^
**8**	54.7	3.64 (±0.09) × 10^4^	1.58 (±0.04) × 10^12^
**9**	73.6	8.90 (±0.30) × 10^4^	3.87 (±0.13) × 10^12^
**10**	61.2	1.38 (±0.04) × 10^4^	5.99 (±0.16) × 10^11^
**11**	60.3	4.00 (±0.14) × 10^4^	1.74 (±0.05) × 10^12^
**12**	61.4	1.63 (±0.04) × 10^4^	7.07 (±0.18) × 10^11^
**13**	48.2	3.45 (±0.08) × 10^4^	1.50 (±0.03) × 10^12^
**14**	56.1	4.80 (±0.11) × 10^4^	2.09 (±0.05) × 10^12^
**15**	57.7	4.72 (±0.07) × 10^4^	2.05 (±0.03) × 10^12^
**16**	59.6	3.76 (±0.07) × 10^4^	1.63 (±0.03) × 10^12^
**17**	58.8	3.60 (±0.08) × 10^4^	1.57 (±0.04) × 10^12^
**18**	56.0	4.47 (±0.06) × 10^4^	1.94 (±0.02) × 10^12^
**19**	44.4	1.99 (±0.08) × 10^4^	8.64 (±0.37) × 10^11^
**20**	60.7	4.07 (±0.08) × 10^4^	1.77 (±0.04) × 10^12^
**21**	70.5	4.74 (±0.09) × 10^4^	2.06 (±0.04) × 10^12^
**22**	66.2	3.06 (±0.09) × 10^4^	1.33 (±0.04) × 10^12^
**23**	57.3	2.85 (±0.09) × 10^4^	1.24 (±0.04) × 10^12^
**24**	68.4	7.10 (±0.17) × 10^4^	3.09 (±0.08) × 10^12^
**25**	49.8	3.46 (±0.07) × 10^4^	1.50 (±0.03) × 10^12^
**26**	69.4	5.27 (±0.14) × 10^4^	2.29 (±0.06) × 10^12^
**27**	53.5	3.38 (±0.10) × 10^4^	1.47 (±0.04) × 10^12^
**28**	69.7	7.19 (±0.20) × 10^4^	3.13 (±0.09) × 10^12^
**29**	66.6	4.04 (±0.10) × 10^4^	1.76 (±0.05) × 10^12^
**30**	42.7	3.62 (±0.04) × 10^4^	1.57 (±0.02) × 10^12^

**Table 3:** Singlet oxygen generation yields (Φ_Δ_), UV-vis absorption maxima used for irradiation of compounds **8**, **18** and **28** in DMF.

Compound	ΦΔ ± SD	λ_Abs_ [nm]
**8**	0.157 ± 0.008	335
**18**	0.845 ± 0.006	325
**28**	0.795 ± 0.006	330

**Table 4:** Calculated energies and interactions of compounds **1**–**30** with DNA.

No. of Compound	Energy (Kcal/mol)	Interactions (PyMol) Polar Contacts
**1**	−6.9	DG16, DG10, DC11
**2**	−6.6	DG16, DG10, DC11
**3**	−6.8	DG16, DG10, DC11
**4**	−6.9	DG16, DG10, DC11
**5**	−6.9	DG16, DG10, DC11, DC9
**6**	−6.9	DG16, DG10, DC11
**7**	−6.7	DG16, DG10, DC11, DC15
**8**	−7.0	DG16, DG10, DC15
**9**	−7.0	DG16, DG10, DC11, DG12, DA17
**10**	−6.7	DG16, DG10, DC11
**11**	−6.8	DG16, DG10
**12**	−6.4	DG16, DG10, DC11
**13**	−6.9	DG16, DG10, DC11
**14**	−6.8	DG16, DG10, DC11
**15**	−6.9	DG16, DG10, DC11
**16**	−6.9	DG16, DG10, DC11
**17**	−7.0	DG16, DG10, DC11, DG14
**18**	−7.4	DG16, DG10, DC11, DG14
**19**	−6.9	DG16, DG10, DC11, DG12, DA17
**20**	−6.9	DG16, DG10, DC11
**21**	−8.3	DG16, DG10, DC11
**22**	−7.9	DG16, DG10, DC11
**23**	−8.3	DG16, DG10, DC11
**24**	−8.3	DG16, DG10, DC11
**25**	−8.3	DG16, DG10, DC11
**26**	−8.3	DG16, DG10, DC11
**27**	−8.2	DG10, DC11
**28**	−8.8	DG16, DG10, DC11
**29**	−8.1	DG16, DG10, DC11
**30**	−8.1	DG16, DG10, DC11

## Data Availability

All necessary supplementary data are included in the Supplementary Materials.

## Supplementary Materials

The following supporting information can be downloaded at: https://www.mdpi.com/article/10.3390/biom16040551/s1, Figures S2.1–2.2: DNA binding with CT-DNA by UV-vis spectroscopy; Figures S3.1–3.2: EB-displacement studies; Figure S4: Copies of UV-Vis spectra of compounds 1–30; Figures S5.1–5.4: Gel electrophoresis pictures uncropped; Figure S6: First-order plots for oxidation of the DPBF by singlet oxygen, for 8, 18 and 28 in DMF; Figure S7: In silico Molecular Dockings of compounds 1–30 with DNA; Figure S8: Control cell culture experiments with UV irradiation.

## Abbreviations

The following abbreviations are used in this manuscript:

CT	Calf-thymus
DMF	Dimethylformamide
DMSO	Dimethylsulfoxide
DPBF	1,3-Diphenylisobenzofuran
EB	Ethidium bromide
HDAC	Histone Diacetylase
HMA	Hydroxamic Acid
K_b_	DNA-binding constant
K_q_	Quenching constant
K_SV_	Stern–Volmer constant
PDT	PhotoDynamic Therapy
QNZ	Quinazolinone
SAR	Structure–Activity Relationship

## References

[1] Al Shaer D., Al Musaimi O., de la Torre B.G., Albericio F. (2020). Hydroxamate Siderophores: Natural Occurrence, Chemical Synthesis, Iron Binding Affinity and Use as Trojan Horses against Pathogens. Eur. J. Med. Chem..

[2] Chen L., Li Z.Y., Wang G.Y. (2025). Siderophores Produced by Marine Microorganisms. Antonie Van Leeuwenhoek Int. J. Gen. Mol. Microbiol..

[3] Swayambhu G., Bruno M., Gulick A.M., Pfeifer B.A. (2021). Siderophore Natural Products as Pharmaceutical Agents. Curr. Opin. Biotechnol..

[4] Miao Z.Y., Lin J., Chen W.M. (2025). Natural Sideromycins and Siderophore-Conjugated Natural Products as Inspiration for Novel Antimicrobial Agents. Eur. J. Med. Chem..

[5] Schalk I.J. (2018). Siderophore–Antibiotic Conjugates: Exploiting Iron Uptake to Deliver Drugs into Bacteria. Clin. Microbiol. Infect..

[6] Laurent Q., Batchelor L.K., Dyson P.J. (2018). Applying a Trojan Horse Strategy to Ruthenium Complexes in the Pursuit of Novel Antibacterial Agents. Organometallics.

[7] Das T., Bhar S., Ghosh D., Kabi B., Kar K., Chandra A. (2025). A Promising Future for Breast Cancer Therapy with Hydroxamic Acid-Based Histone Deacetylase Inhibitors. Bioorg. Chem..

[8] Cheng Y., Zhuang Y., Zhu N., Yu Y., Hu M. (2025). Hydroxamic Acid Hybrids as Histone Deacetylase Inhibitors for Cancer Therapy: An Update (2022 to Date). Bioorg. Chem..

[9] Citarella A., Moi D., Pinzi L., Bonanni D., Rastelli G. (2021). Hydroxamic Acid Derivatives: From Synthetic Strategies to Medicinal Chemistry Applications. ACS Omega.

[10] Cao X., Gong Y. (2024). Recent Developments of Hydroxamic Acid Hybrids as Potential Anti-Breast Cancer Agents. Future Med. Chem..

[11] Ribeiro M., Simões M. (2019). Advances in the Antimicrobial and Therapeutic Potential of Siderophores. Environ. Chem. Lett..

[12] Khasheii B., Mahmoodi P., Mohammadzadeh A. (2021). Siderophores: Importance in Bacterial Pathogenesis and Applications in Medicine and Industry. Microbiol. Res..

[13] Sugamata K. (2025). Hydroxamate-Based Metal-Organic Frameworks. Chem. Eur. J..

[14] Pawar T.J., Ventura-Hernández K.I., Ramos-Morales F.R., Olivares-Romero J.L. (2023). Chiral Hydroxamic Acid Ligands in the Asymmetric Synthesis of Natural Products. Chemistry.

[15] Soares E.V. (2022). Perspective on the Biotechnological Production of Bacterial Siderophores and Their Use. Appl. Microbiol. Biotechnol..

[16] Thomas M., Alsarraf J., Araji N., Tranoy-Opalinski I., Renoux B., Papot S. (2019). The Lossen Rearrangement from Free Hydroxamic Acids. Org. Biomol. Chem..

[17] Shen S., Kozikowski A.P. (2016). Why Hydroxamates May Not Be the Best Histone Deacetylase Inhibitors—What Some May Have Forgotten or Would Rather Forget?. ChemMedChem.

[18] Majewski M.W., Cho S., Miller P.A., Franzblau S.G., Miller M.J. (2015). Syntheses and Evaluation of Substituted Aromatic Hydroxamates and Hydroxamic Acids That Target Mycobacterium Tuberculosis. Bioorg. Med. Chem. Lett..

[19] Bondar D., Bragina O., Lee J.Y., Semenyuta I., Järving I., Brovarets V., Wipf P., Bahar I., Karpichev Y. (2023). Hydroxamic Acids as PARP-1 Inhibitors: Molecular Design and Anticancer Activity of Novel Phenanthridinones. Helv. Chim. Acta.

[20] Zhao Y., Shakeri A., Hefny A.A., Rao P.P.N. (2024). N-Benzyl, N-Phenethyl and N-Benzyloxybenzamide Derivatives Inhibit Amyloid-Beta (Aβ42) Aggregation and Mitigate Aβ42-Induced Neurotoxicity. Med. Chem. Res..

[21] Gatadi S., Lakshmi T.V., Nanduri S. (2019). 4(3H)-Quinazolinone Derivatives: Promising Antibacterial Drug Leads. Eur. J. Med. Chem..

[22] Khan I., Ibrar A., Ahmed W., Saeed A. (2015). Synthetic Approaches, Functionalization and Therapeutic Potential of Quinazoline and Quinazolinone Skeletons: The Advances Continue. Eur. J. Med. Chem..

[23] Welsch M.E., Snyder S.A., Stockwell B.R. (2010). Privileged Scaffolds for Library Design and Drug Discovery. Curr. Opin. Chem. Biol..

[24] Liu J.F., Wilson C.J., Ye P., Sprague K., Sargent K., Si Y., Beletsky G., Yohannes D., Ng S.C. (2006). Privileged Structure-Based Quinazolinone Natural Product-Templated Libraries: Identification of Novel Tubulin Polymerization Inhibitors. Bioorg. Med. Chem. Lett..

[25] Jafari E., Khajouei M.R., Hassanzadeh F., Hakimelahi G.H., Khodarahmi G.A. (2016). Quinazolinone and Quinazoline Derivatives: Recent Structures with Potent Antimicrobial and Cytotoxic Activities. Res. Pharm. Sci..

[26] Chen K., Wang S., Fu S., Kim J., Park P., Liu R., Lei K. (2025). 4(3H)-Quinazolinone: A Natural Scaffold for Drug and Agrochemical Discovery. Int. J. Mol. Sci..

[27] He D., Wang M., Zhao S., Shu Y., Zeng H., Xiao C., Lu C., Liu Y. (2017). Pharmaceutical Prospects of Naturally Occurring Quinazolinone and Its Derivatives. Fitoterapia.

[28] Wei Y., Zheng F., Guo L., Chen W., Wang H. (2025). Natural pyrrolo [1,2-α] quinazolinone Derivatives: Design, Synthesis, Characterization, and Bio-Evaluation as Novel Antiviral Agents. Eur. J. Med. Chem..

[29] Long S., Resende D.I.S.P., Kijjoa A., Silva A.M.S., Pina A., Fernández-Marcelo T., Helena Vasconcelos M., Sousa E., Pinto M.M.M. (2018). Antitumor Activity of Quinazolinone Alkaloids Inspired by Marine Natural Products. Mar. Drugs.

[30] Hieu D.T., Anh D.T., Hai P.T., Thuan N.T., Huong L.T.T., Park E.J., Young Ji A., Soon Kang J., Phuong Dung P.T., Han S.B. (2019). Quinazolin-4(3H)-One-Based Hydroxamic Acids: Design, Synthesis and Evaluation of Histone Deacetylase Inhibitory Effects and Cytotoxicity. Chem. Biodivers..

[31] Dung N.P., Kim H.K., Huyen V.T.M., Kang D.H., Kim H.Y., Kang J.S., Anh D.T., Tung T.T., Han S.B., Nam N.H. (2026). Design, Synthesis, and Bioevaluation of 1H-1,2,3-Triazole-Linked Quinazolin-4(3H)-One Hydroxamic Acids as Novel Histone Deacetylase Inhibitors. J. Mol. Struct..

[32] Hieu D.T., Anh D.T., Tuan N.M., Hai P.T., Huong L.T.T., Kim J., Kang J.S., Vu T.K., Dung P.T.P., Han S.B. (2018). Design, Synthesis and Evaluation of Novel N-Hydroxybenzamides/N-Hydroxypropenamides Incorporating Quinazolin-4(3H)-Ones as Histone Deacetylase Inhibitors and Antitumor Agents. Bioorg. Chem..

[33] Osipov V.N., Khachatryan D.S., Balaev A.N. (2020). Biologically Active Quinazoline-Based Hydroxamic Acids. Med. Chem. Res..

[34] Vu T.K., Thanh N.T., Minh N.V., Linh N.H., Thao N.T.P., Nguyen T.T.B., Hien D.T., Chinh L.V., Duc T.H., Anh L.D. (2021). Novel Conjugated Quinazolinone-Based Hydroxamic Acids: Design, Synthesis and Biological Evaluation. Med. Chem..

[35] Nara H., Sato K., Kaieda A., Oki H., Kuno H., Santou T., Kanzaki N., Terauchi J., Uchikawa O., Kori M. (2016). Design, Synthesis, and Biological Activity of Novel, Potent, and Highly Selective Fused Pyrimidine-2-Carboxamide-4-One-Based Matrix Metalloproteinase (MMP)-13 Zinc-Binding Inhibitors. Bioorg. Med. Chem..

[36] Thakur A., Tawa G.J., Henderson M.J., Danchik C., Liu S., Shah P., Wang A.Q., Dunn G., Kabir M., Padilha E.C. (2020). Design, Synthesis, and Biological Evaluation of Quinazolin-4-One-Based Hydroxamic Acids as Dual PI3K/HDAC Inhibitors. J. Med. Chem..

[37] Mikra C., Melissari Z., Kokotou M.G., Gritzapis P., Fylaktakidou K.C. (2022). Microwave-Assisted Synthesis of Hydroxamic Acid Incorporated Quinazolin-4[3H]-One Derivatives. Sustain. Chem. Pharm..

[38] El Sayed N.A., Eissa A.A., El Masry G.F., Abdullah M.M., Arafa R.K. (2016). Discovery of Novel Quinazolinones and Their Acyclic Analogues as Multi-Kinase Inhibitors: Design, Synthesis, SAR Analysis and Biological Evaluation. RSC Adv..

[39] El-Hashash M.A.E.A., Salem M.S., Al-Mabrook S.A.M. (2018). Synthesis and Anticancer Activity of Novel Quinazolinone and Benzamide Derivatives. Res. Chem. Intermed..

[40] Ahmed M.F., Hashim A.A. (2016). Design, Synthesis of Novel Quinazolin-4-One Derivatives and Biological Evaluation against Human MCF-7 Breast Cancer Cell Line. Res. Chem. Intermed..

[41] El-Hashash M.A.E.A., Azab M.E.-A., Faty R.A.E.-A., Amr A.E.-G.E. (2016). Synthesis, Antimicrobial and Anti-Inflammatory Activity of Some New Benzoxazinone and Quinazolinone Candidates. Chem. Pharm. Bull..

[42] El-Hashash M.A., Rizk S.A., El-Naggar A.M., El-Bana M.G. (2017). Regiospecific Isomerization of 2-Benzoxazinon-2-Yl Benzoic Acid Toward Some Nitrogen Nucleophiles as Environmental Insecticide. J. Heterocycl. Chem..

[43] Deore R.R., Chen G.S., Chang P.T., Chern T.R., Lai S.Y., Chuang M.H., Lin J.H., Kung F.L., Chen C.S., Chiou C.T. (2012). Discovery of N-Arylalkyl-3-Hydroxy-4-Oxo-3,4-Dihydroquinazolin-2-Carboxamide Derivatives as HCV NS5B Polymerase Inhibitors. ChemMedChem.

[44] Alagha A., Parthasarathi L., Gaynor D., Müller-Bunz H., Starikova Z.A., Farkas E., O’Brien E.C., Gil M.J., Nolan K.B. (2011). Metal Complexes of Cyclic Hydroxamates. Synthesis and Crystal Structures of 3-Hydroxy-2-Methyl-3H-Quinazolin-4-One (ChaH) and of Its Fe(III), Co(II), Ni(II), Cu(II) and Zn(II) Complexes. Inorganica Chim. Acta.

[45] Osipov V.N., Vartanyan A.A., Khochenkov D.A., Gusev D.V., Fateenkova O.V., Khachatryan D.S., Borisova L.M. (2024). 3-Hydroxyquinazoline Derivatives, Analogues of Erastin, Induce Ferroptosis in Colorectal Cancer Cells. Russ. J. Bioorg. Chem..

[46] Pandiri S., Konda S.K., Chennuri B.K., Akarapu P., Kuncham M., Korra R., Bhoomandla S. (2024). Novel Oxo-5-(Trifluoromethyl)Quinazolinyl Amide Derivatives, Their Anticancer Activity and Docking Interactions. Russ. J. Gen. Chem..

[47] Maphutha J., Twilley D., Dawood M., Efferth T., Lall N. (2026). The Potential of Phytochemicals to Overcome Multidrug Resistance in Metastatic Melanoma. Chem. Biodivers..

[48] Hamblin M.R., Huang Y.Y., Vecchio D., Avci P., Yin R., Garcia-Diaz M. (2013). Melanoma Resistance to Photodynamic Therapy: New Insights. Biol. Chem..

[49] Aebisher D., Rogó K., Yakub Z.A., Dynarowicz K., Myśliwiec A., Mytych W., Komosińska-Vassev K., Misiołek M., Kawczyk-krupka A. (2024). Photodynamic Therapy in Glioma Cell Culture. Oncologie.

[50] Li X.Y., Tan L.C., Dong L.W., Zhang W.Q., Shen X.X., Lu X., Zheng H., Lu Y.G. (2020). Susceptibility and Resistance Mechanisms During Photodynamic Therapy of Melanoma. Front. Oncol..

[51] Bosio G.N., Parisi J., García Einschlag F.S., Mártire D.O. (2018). Imidazole and Beta-Carotene Photoprotection against Photodynamic Therapy Evaluated by Synchrotron Infrared Microscopy. Spectrochim. Acta A Mol. Biomol. Spectrosc..

[52] Figueroa-Figueroa D.I., Lechuga-Millán A., Vargas-Castro R., Ramon-Gallegos E., García-Becerra R., Hernández-Luis F. (2026). Novel Quinazoline-Ferrocene Photosensitizer: In Silico Studies, Photochemical Characterization, ROS Generation, and in Vitro Phototoxicity. J. Photochem. Photobiol. A Chem..

[53] Mikra C., Bairaktari M., Petridi M.T., Detsi A., Fylaktakidou K.C. (2022). Green Process for the Synthesis of 3-Amino-2-Methyl-Quinazolin-4(3H)-One Synthones and Amides Thereof:DNA Photo-Disruptive and Molecular Docking Studies. Processes.

[54] Panagopoulos A., Balalas T., Mitrakas A., Vrazas V., Katsani K.R., Koumbis A.E., Koukourakis M.I., Litinas K.E., Fylaktakidou K.C. (2021). 6-Nitro-Quinazolin−4(3H)−one Exhibits Photodynamic Effects and Photodegrades Human Melanoma Cell Lines. A Study on the Photoreactivity of Simple Quinazolin−4(3H)−ones. Photochem. Photobiol..

[55] Lazou M., Tarushi A., Gritzapis P., Psomas G. (2020). Transition Metal Complexes with a Novel Guanine-Based (E)-2-(2-(Pyridin-2-Ylmethylene)Hydrazinyl)Quinazolin-4(3H)-One: Synthesis, Characterization, Interaction with DNA and Albumins and Antioxidant Activity. J. Inorg. Biochem..

[56] Soultas S., Meliopoulos K., Psomas G., Katsani K.R., Fylaktakidou K.C. (2024). Oxime Esters on 4-Nitrobenzaldehyde and 9,10-Anthraquinone-2-Carboxaldehyde Templates: DNA- and Albumin-Binding and Photocleavage Studies. Arkivoc.

[57] Mitrakas A., Stathopoulou M.E.K., Mikra C., Konstantinou C., Rizos S., Malichetoudi S., Koumbis A.E., Koffa M., Fylaktakidou K.C. (2024). Synthesis of 2-Amino-N′-Aroyl(Het)Arylhydrazides, DNA Photocleavage, Molecular Docking and Cytotoxicity Studies against Melanoma CarB Cell Lines. Molecules.

[58] Mikra C., Mitrakas A., Ghizzani V., Katsani K.R., Koffa M., Koukourakis M., Psomas G., Protti S., Fagnoni M., Fylaktakidou K.C. (2023). Effect of Arylazo Sulfones on DNA: Binding, Cleavage, Photocleavage, Molecular Docking Studies and Interaction with A375 Melanoma and Non-Cancer Cells. Int. J. Mol. Sci..

[59] Panagopoulos A., Alipranti K., Mylona K., Paisidis P., Rizos S., Koumbis A.E., Roditakis E., Fylaktakidou K.C. (2023). Exploration of the DNA Photocleavage Activity of O-Halo-Phenyl Carbamoyl Amidoximes: Studies of the UVA-Induced Effects on a Major Crop Pest, the Whitefly Bemisia Tabaci. DNA.

[60] Gritzapis P.S., Varras P.C., Andreou N.P., Katsani K.R., Dafnopoulos K., Psomas G., Peitsinis Z.V., Koumbis A.E., Fylaktakidou K.C. (2020). p-Pyridinyl Oxime Carbamates: Synthesis, DNA Binding, DNA Photocleaving Activity and Theoretical Photodegradation Studies. Beilstein J. Org. Chem..

[61] Pasolli M., Dafnopoulos K., Andreou N.P., Gritzapis P.S., Koffa M., Koumbis A.E., Psomas G., Fylaktakidou K.C. (2016). Pyridine and P-Nitrophenyl Oxime Esters with Possible Photochemotherapeutic Activity: Synthesis, DNA Photocleavage and DNA Binding Studies. Molecules.

[62] Marmur J.A. (1961). Procedure for the Isolation of Deoxyribonucleic Acid from Micro-Organisms. J. Mol. Biol..

[63] Reichmann M.E., Rice S.A., Thomas C.A., Doty P. (1954). A Further Examination of the Molecular Weight and Size of Desoxypentose Nucleic Acid. J. Am. Chem. Soc..

[64] Wieczorek E., Mlynarczyk D.T., Kucinska M., Dlugaszewska J., Piskorz J., Popenda L., Szczolko W., Jurga S., Murias M., Mielcarek J. (2018). Photophysical Properties and Photocytotoxicity of Free and Liposome-Entrapped Diazepinoporphyrazines on LNCaP Cells under Normoxic and Hypoxic Conditions. Eur. J. Med. Chem..

[65] Durmus M., Ahsen V., Nyokong T. (2007). Photophysical and Photochemical Studies of Long Chain-Substituted Zinc Phthalocyanines. J. Photochem. Photobiol. A Chem..

[66] Porolnik W., Ratajczak M., Mackowiak A., Murias M., Kucinska M., Piskorz J. (2024). Liposomal Formulations of Novel BODIPY Dimers as Promising Photosensitizers for Antibacterial and Anticancer Treatment. Molecules.

[67] Frisch M.J., Trucks G.W., Schlegel H.B., Scuseria G.E., Robb M.A., Cheeseman J.R., Scalmani G., Barone B., Mennucci G.A., Petersson H. (2009). Gaussian 09.

[68] Drew H.R., Dickerson R.E. (1981). Structure of a B-DNA Dodecamer. III. Geometry of Hydration. J. Mol. Biol..

[69] Trott O., Olson A.J. (2010). Software News and Update AutoDock Vina: Improving the Speed and Accuracy of Docking with a New Scoring Function, Efficient Optimization, and Multithreading. J. Comput. Chem..

[70] Schrodinger, Inc. (2009). The PyMOL Molecular Graphics System.

[71] Wolfe A., Shimer G.H., Meehan T. (1987). Polycyclic Aromatic Hydrocarbons Physically Intercalate into Duplex Regions of Denatured DNA. Biochemistry.

[72] Pizarro A.M., Sadler P.J. (2009). Unusual DNA Binding Modes for Metal Anticancer Complexes. Biochimie.

[73] Lakowicz J.R. (2006). Principles of Fluorescence Spectroscopy.

[74] Heller D.P., Greenstock C.L. (1994). Fluorescence Lifetime Analysis of DNA Intercalated Ethidium Bromide and Quenching by Free Dye. Biophys. Chem..

[75] Seotsanyana-Mokhosi I., Kuznetsova N., Nyokong T. (2001). Photochemical Studies of Tetra-2,3-Pyridinoporphyrazines. J. Photochem. Photobiol. A Chem..

[76] Mir M., Jansen L.M.G., Wilkinson F., Bourdelande J.L., Marquet J. (1998). Efficiency of Singlet Oxygen Generation from the Triplet States of Nitrophenyl Ethers. J. Photochem. Photobiol. A Chem..

