# Supplementary material for: DNA Binding, DNA Photocleavage, Molecular Docking Studies and Photo-Induced Effect on Melanoma Cells of 2-Methyl-3-OR Quinazolinone Derivatives
Source: Biomolecules. 2026 Apr 8;16(4):551. doi: 10.3390/biom16040551 (PMC13113181; doi:10.3390/biom16040551)

Supporting Information

DNA binding, DNA photocleavage, Molecular Docking Studies and Photo-induced Effect on Melanoma Cells of 2-Methyl-3-OR Quinazolinone Derivatives

Chrysoula Mikra [1], Stella Malichetoudi [2], Dimitrios Arampatzis [2], Ioanna Laskari [2], Maria Koffa [2], Ewelina Wieczorek-Szweda[3], Katerina R. Katsani [4], George Psomas [5] and Konstantina C. Fylaktakidou [1,*]

[1] Laboratory of Organic Chemistry, Faculty of Chemistry, Aristotle University of Thessaloniki, GR-54124 Thessaloniki, Greece; chrmikgeo@chem.auth.gr (C.M.)
[2] Laboratory of Cellular Biology, Molecular Biology and Genetics Department, Democritus University of Thrace, University Campus, GR-68100 Alexandroupolis, Greece; stelmali1@mbg.duth.gr (S.M.); dimitrios.b.arabatzis@gmail.com; ioanna.elkat@gmail.com; mkoffa@mbg.duth.gr (M.K.)
[3] Department of Functional Nanostructures Synthesis, Faculty of Chemistry, Adam Mickiewicz University, PL-61614 Poznań, Poland; ewelina.wieczorek-szweda@amu.edu.pl
[4] Laboratory of Biochemistry and Molecular Virology, Molecular Biology and Genetics Department, Democritus University of Thrace, Dragana, GR-68100 Alexandroupolis, Greece; kkatsani@mbg.duth.gr
[5] Laboratory of Inorganic Chemistry, Department of Chemistry, Aristotle University of Thessaloniki, GR-54124 Thessaloniki, Greece; gepsomas@chem.auth.gr

* Correspondence: kfylakta@chem.auth.gr

Table of contents

		page
S1	***Interaction with CT–DNA***	S3
S1.1	*Binding study with CT–DNA by UV-vis spectroscopy*	
S1.2	*CT–DNA-binding studies by viscosity measurements*	
S1.3	*EB-displacement studies*	
S1.4	*References*	
S2	***Figures: DNA binding with CT-DNA by UV-vis spectroscopy***	S5
S2.1	*UV-vis spectra of compound 1-30 in DMSO in the presence of increasing amounts of CT DNA*	
S2.2	***Plot of*** $\frac{[DNA]}{(\varepsilon_A - \varepsilon_f)}$ ***versus [DNA] of compounds 1-30***	
S3	***EB-displacement studies***	S16
S3.1	*Fluorescence emission spectra (λexc = 540 nm) for EB-DNA conjugate ([EB] = 20 μM, [CT DNA] = 26 μM) in buffer solution (150 mM NaCl and 15 mM trisodium citrate at pH = 7.0) in the presence of increasing amounts of compounds* ***1-30****.*	
S3.2	*Stern–Volmer quenching plot of EB–DNA fluorescence of compounds* ***1-30****.*	
S4	***Copies of UV-Vis spectra of compounds 1-30***	S26
S5	***Gel electrophoresis pictures uncropped***	S31
S5.1	*Agarose gel electrophoresis of nitro compounds* ***8, 9, 18, 19, 28, 29*** *in dark (triplicate, 100 μM)*	
S5.2	*Agarose gel electrophoresis of compounds* ***1-10*** *upon irradiation at 365 nm (triplicate, 100 μM)*	
S5.3	*Agarose gel electrophoresis of compounds* ***11-20*** *upon irradiation at 365 nm (triplicate, 100 μM)*	
S5.4	*Agarose gel electrophoresis of compounds* ***21-30*** *upon irradiation at 365 nm (triplicate, 100 μM)*	
S6	***First-order plots for oxidation of the DPBF by singlet oxygen, for 8, 18 and 28 in DMF***	S37
S7	***In silico Molecular Dockings of compounds 1-30 with DNA***	S38
S8	***Control cell culture experiments with UV irradiation***	S44

S1 Interaction with CT DNA

The interaction of the compounds with CT DNA was investigated with UV-vis spectroscopy, viscosity measurements and fluorescence emission spectroscopy studies.

S1.1 Binding study with CT DNA by UV-vis spectroscopy

UV-vis spectroscopy was used for the evaluation of the interaction of the compounds with CT DNA, and specifically the possible binding modes of the compounds to CT DNA. Control experiments with DMSO were performed and no changes in the spectra of CT DNA were observed.

In order to determine the binding mode, the UV-vis spectra of the compounds were recorded for a constant concentration ($5{\times}10^{-5}$ - 10^{-4} M) at the corresponding λ_{max} with increasing concentrations of CT DNA for diverse *r* (*r* = [compound]/[DNA]) values. Effective use of the changes in the absorbance of the UV-vis spectra was made and the DNA-binding constants of the compounds (K_b, in M^{-1}) were calculated by the Wolfe-Shimer equation (eq. S1) [1] and the plots [DNA]/(ε_A–ε_f) *versus* [DNA]:

$$\frac{[DNA]}{(\varepsilon_A-\varepsilon_f)}=\frac{[DNA]}{(\varepsilon_b-\varepsilon_f)}+\frac{1}{K_b(\varepsilon_b-\varepsilon_f)} \qquad \text{(eq S1)}$$

where [DNA] = the concentration of DNA in base pairs, ε_f = the extinction coefficient for the free compound at the corresponding λ_{max}, ε_A = A_{obsd}/[compound] and ε_b = the extinction coefficient for the compound in the fully bound form. K_b is given by the ratio of slope to the y intercept in plots [DNA]/(ε_A–ε_f) *versus* [DNA].

S1.2 CT DNA-binding studies by viscosity measurements

The viscosity of CT DNA (0.1 mM) in buffer solution was measured in the absence and presence of increasing amounts of the compounds. The experiments were performed at room temperature and the measurements are depicted in a plot $(\eta/\eta_0)^{1/3}$ *versus* r (*r* = [compound]/[DNA]), where η = the viscosity of DNA in the presence of the compound, and η_0 = the viscosity of DNA in buffer solution.

S1.3 EB-displacement studies

In order to determine and confirm the CT DNA-binding mode of the compounds, a competitive study with EB as an intercalating marker is performed with fluorescence emission spectroscopy. Therefore, the EB-displacing ability of the compounds from its EB-DNA conjugate was examined.

The DNA-EB adduct was prepared by addition of 20 μM EB and 26 μM CT DNA in buffer solution (150 mM NaCl and 15 mM trisodium citrate at pH 7.0). The potential intercalation of the compounds between the DNA-bases was studied upon the addition of a certain amount of the compound's solution into the EB-DNA solution. The influence of the compounds on the EB-DNA solution was monitored through the changes of the fluorescence emission spectra at excitation wavelength (λ_{ex}) at 540 nm [2]. The tested compounds do not show any significant fluorescence at room temperature in solution or in the presence of DNA, under the same experimental conditions (λ_{ex} = 540 nm). Bearing

that in mind, the observed quenching of the EB-DNA solution is evidently associated with the displacement of EB from its EB-DNA adduct.

The quenching efficiency (K_{SV}) for each compound was assessed according to the Stern-Volmer equation (eq. S2) [2]:

$$\frac{Io}{I} = 1 + k_q \tau_0 [Q] = 1 + K_{SV}[Q] \qquad \text{(eq. S2)}$$

where I_0 and I = the fluorescence emission intensities of EB-DNA in the absence and presence of the quencher, respectively, [Q] = the concentration of the quencher (i.e. compounds). K_{SV} is obtained from the Stern-Volmer plots from the slope of the diagram I_0/I *versus* [Q]. Taking τ_0 = 23 ns as the fluorescence lifetime of the EB-DNA adduct [3], the EB-DNA quenching constants (k_q, in $M^{-1}s^{-1}$) of the compounds can be determined according to equation S3:

$$K_{SV} = k_q \tau_o \qquad \text{(eq. S3)}$$

S1.4 References

1. Wolfe, A.; Shimer, G.H.; Meehan, T. Polycyclic Aromatic Hydrocarbons Physically Intercalate into Duplex Regions of Denatured DNA. *Biochemistry* **1987**, *26*, 6392–6396, doi:10.1021/bi00394a013.
2. Lakowicz, J.. R. *Principles of Fluorescence Spectroscopy*; Springer New York, NY, 2006; ISBN 978-0-387-31278-1.
3. Heller, D.P.; Greenstock, C.L. Fluorescence lifetime analysis of DNA intercalated ethidium bromide and quenching by free dye. *Biophys. Chem.* **1994**, *50*, 305–312, doi:10.1016/0301-4622(93)E0101-A.

Figure S2.1 UV–vis spectra of compound **1–30** in DMSO in the presence of increasing amounts of CT DNA. The arrows show the changes upon increasing amounts of CT DNA

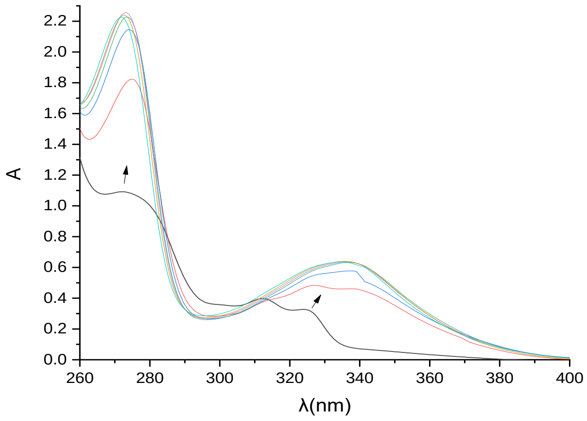

1

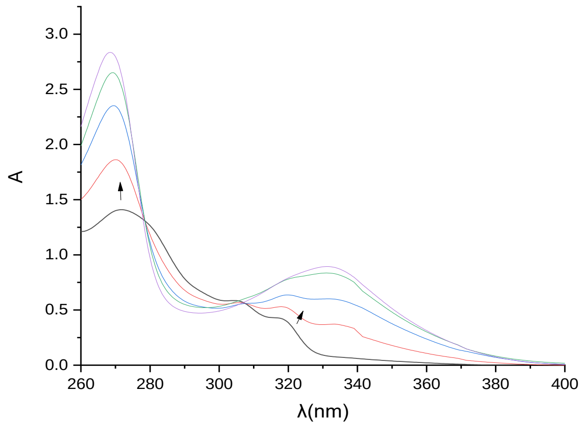

2

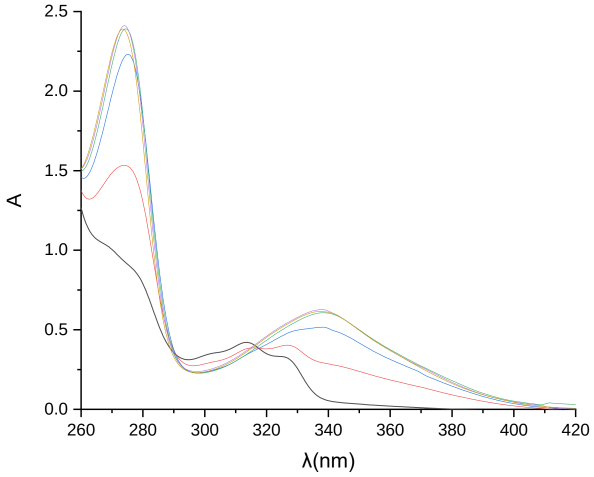

3

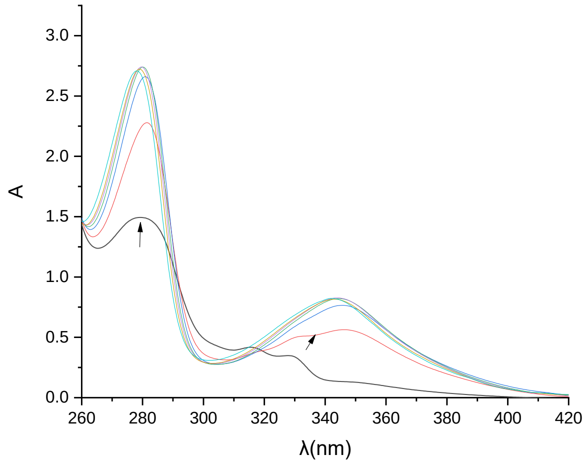

4

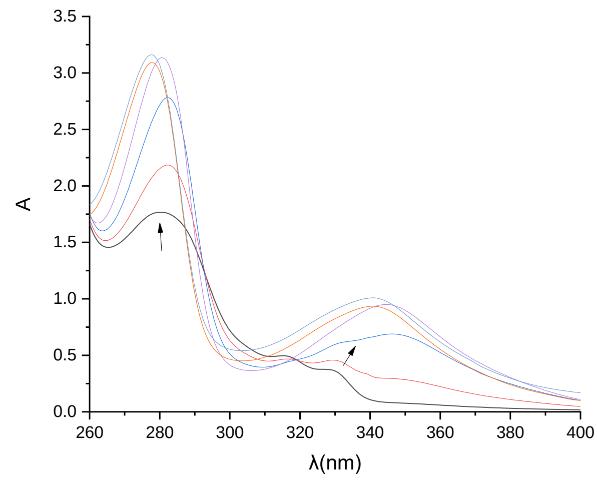
A
3.5
3.0
2.5
2.0
1.5
1.0
0.5
0.0
260 280 300 320 340 360 380 400
λ(nm)

5

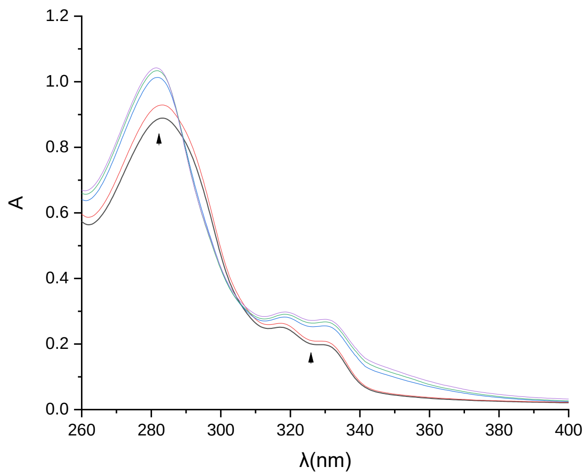
A
1.2
1.0
0.8
0.6
0.4
0.2
0.0
260 280 300 320 340 360 380 400
λ(nm)

6

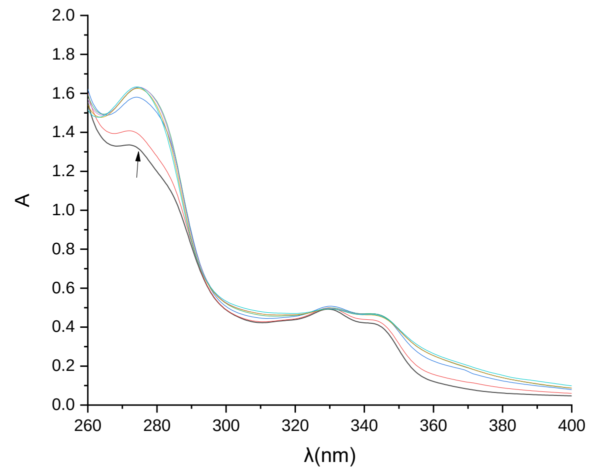
A
2.0
1.8
1.6
1.4
1.2
1.0
0.8
0.6
0.4
0.2
0.0
260 280 300 320 340 360 380 400
λ(nm)

7

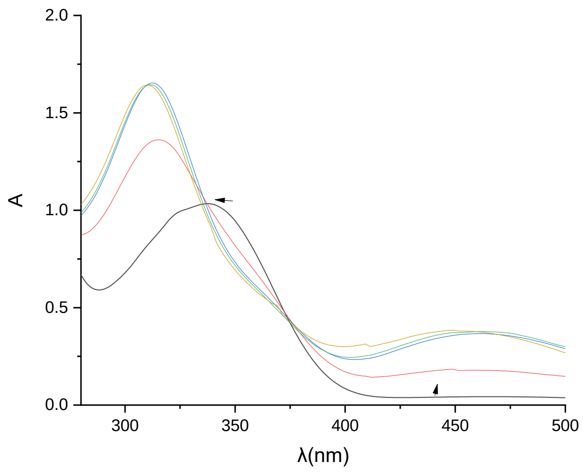
A
2.0
1.5
1.0
0.5
0.0
300 350 400 450 500
λ(nm)

8

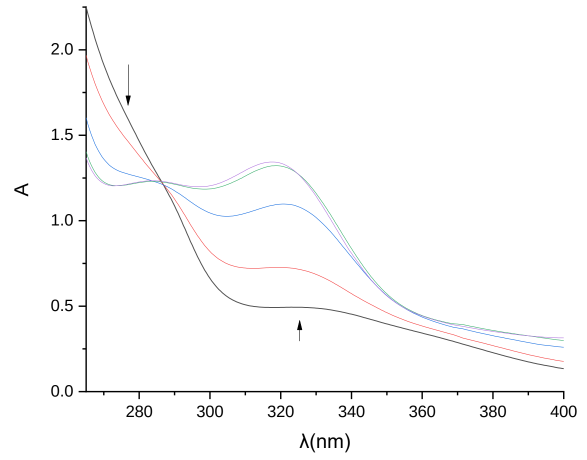
A
2.0
1.5
1.0
0.5
0.0
280 300 320 340 360 380 400
λ(nm)

9

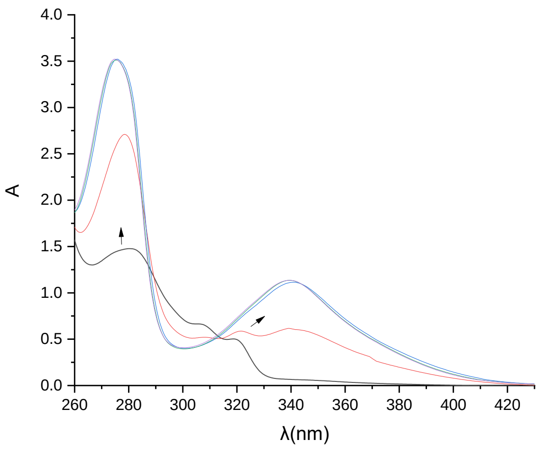
A
4.0
3.5
3.0
2.5
2.0
1.5
1.0
0.5
0.0
260 280 300 320 340 360 380 400 420
λ(nm)

10

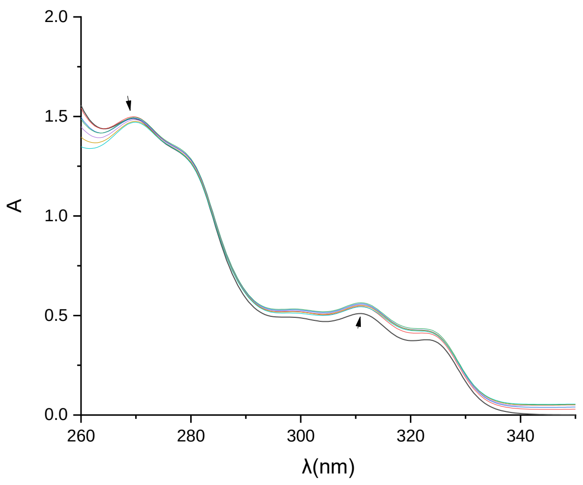

11

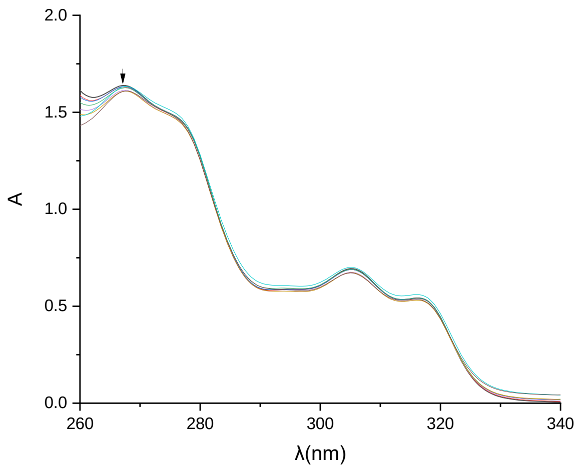

12

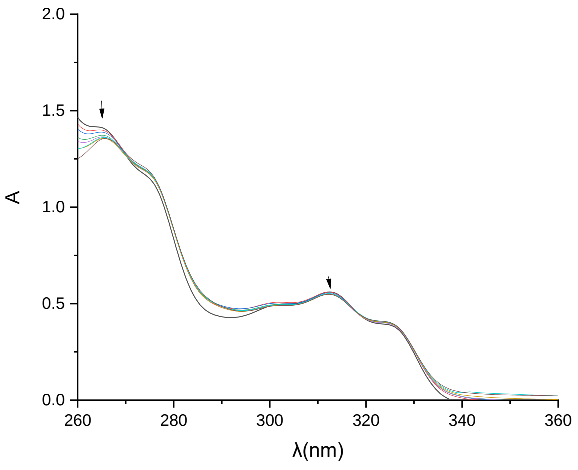

13

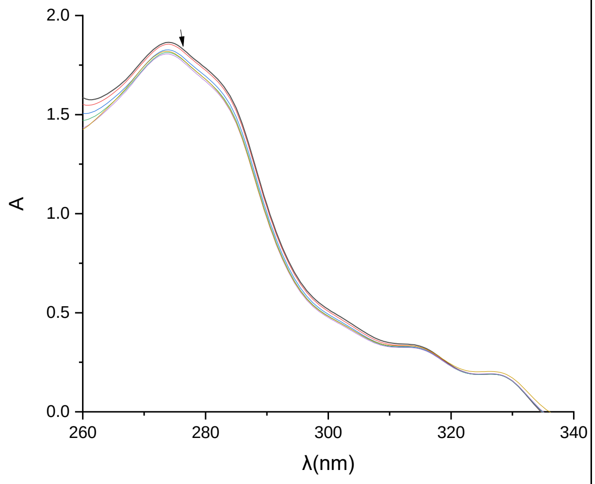

14

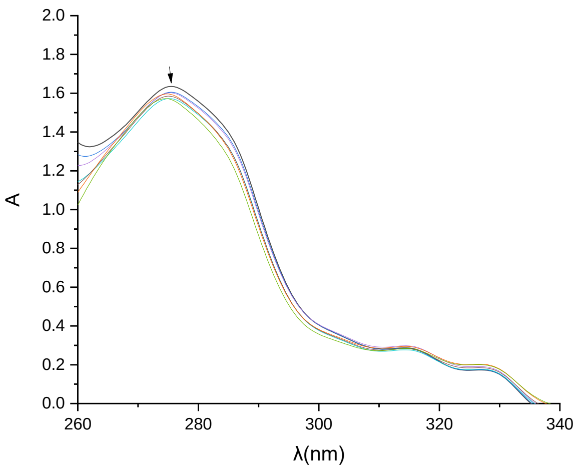

15

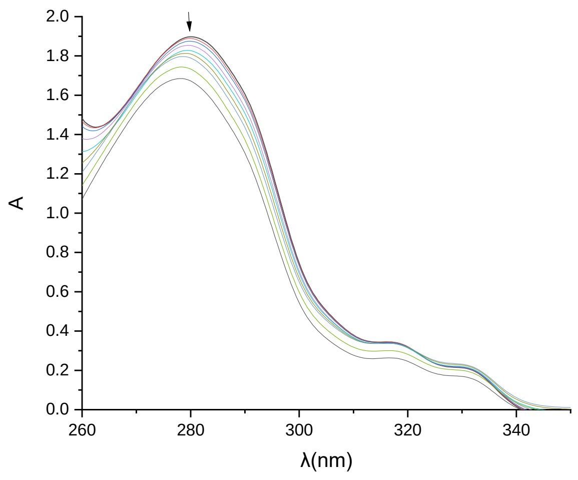

16

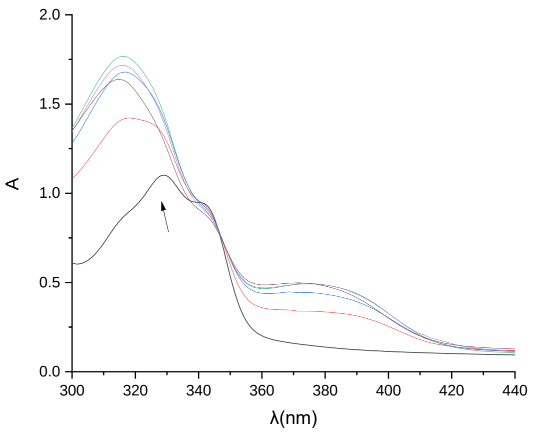

17

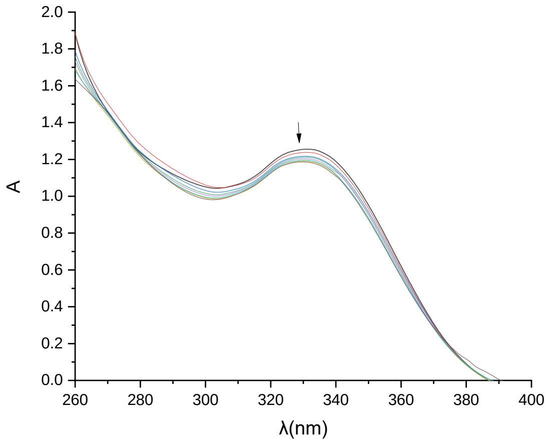

18

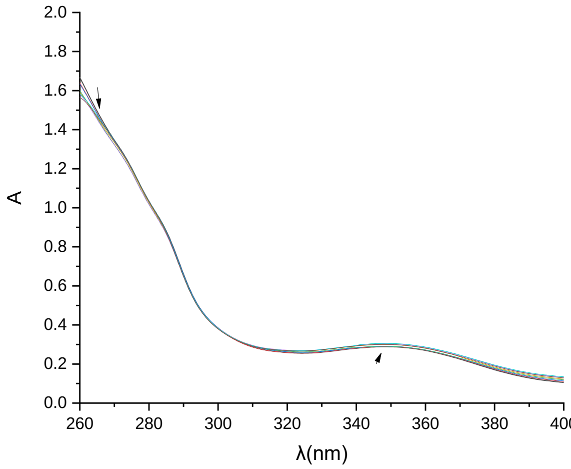

19

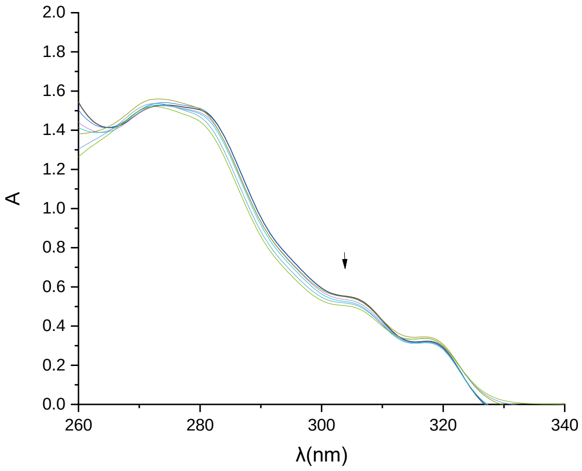

20

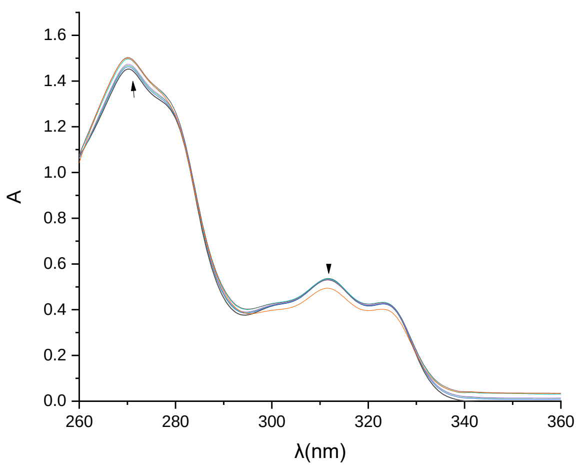

21

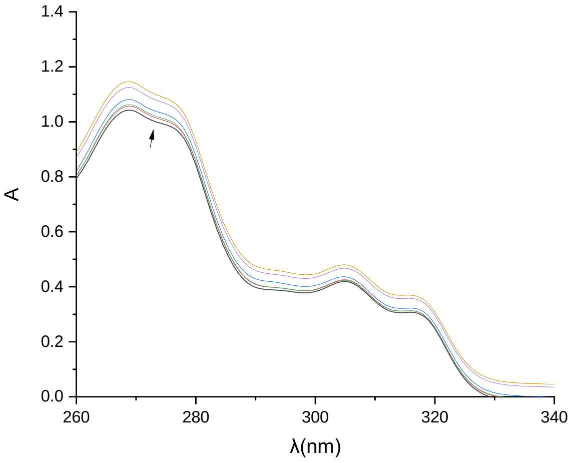

22

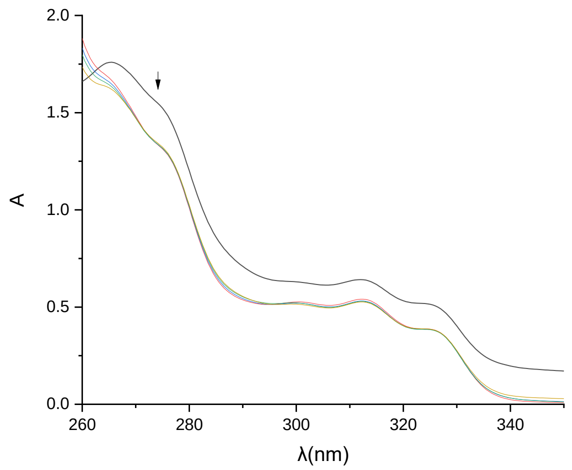

23

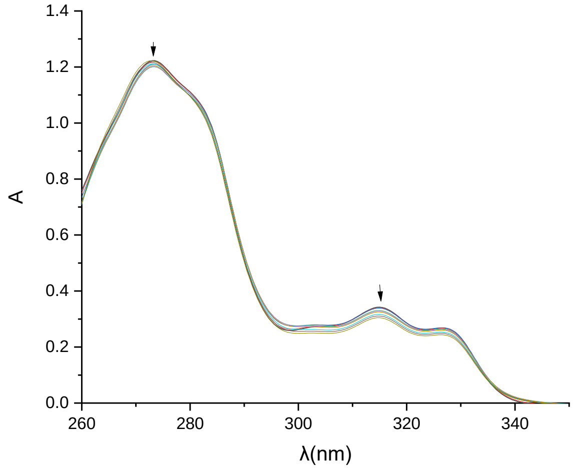

24

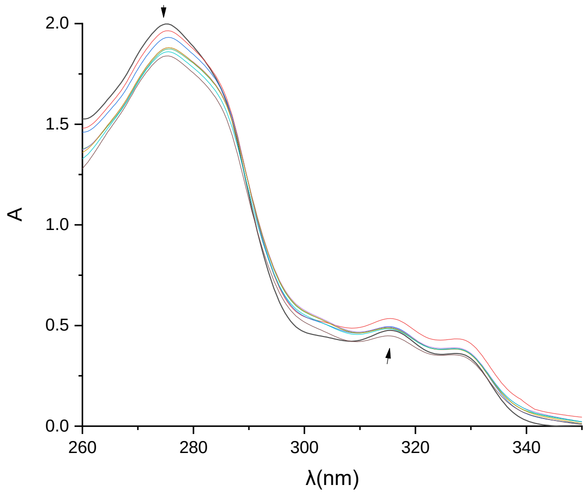

25

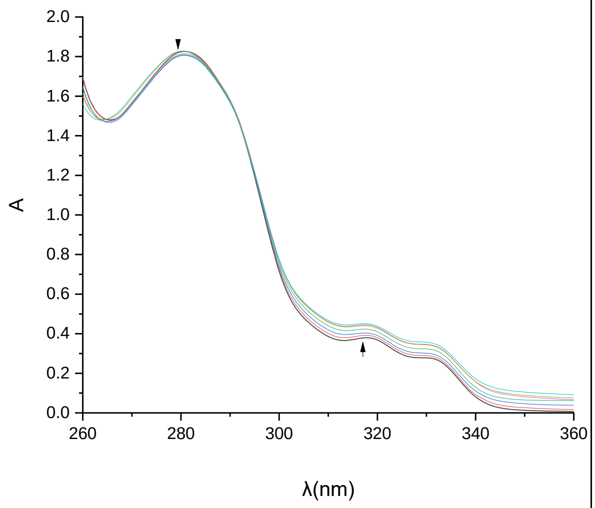

26

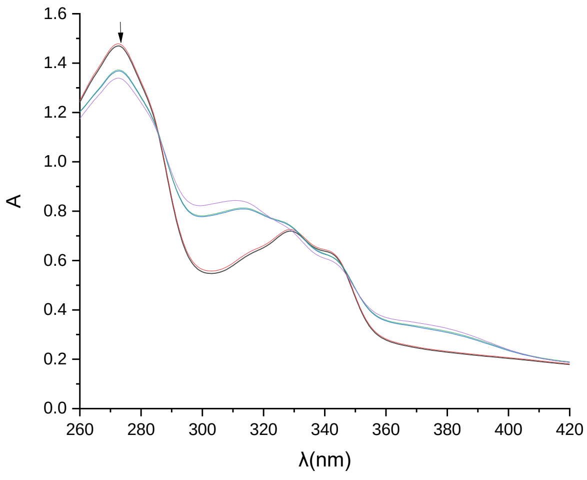

27

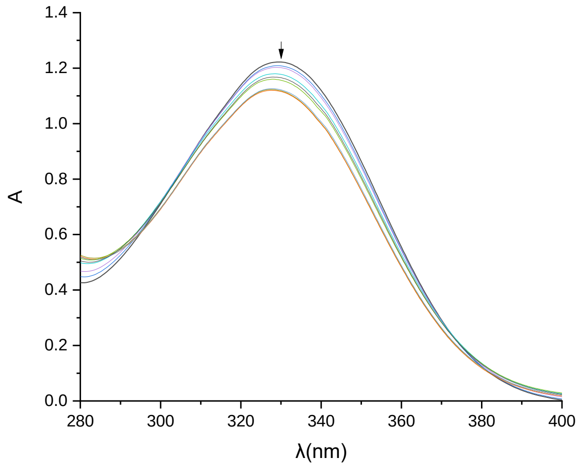

28

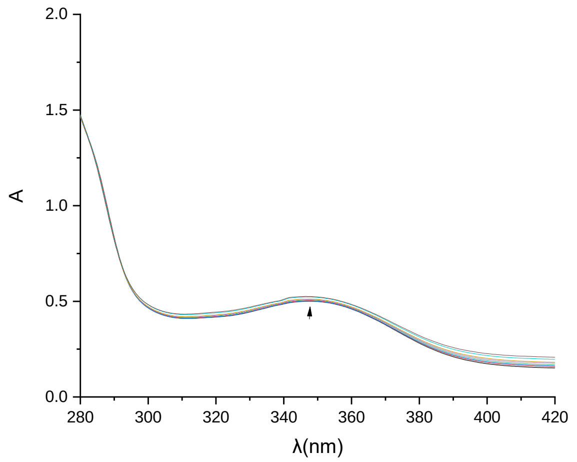

29

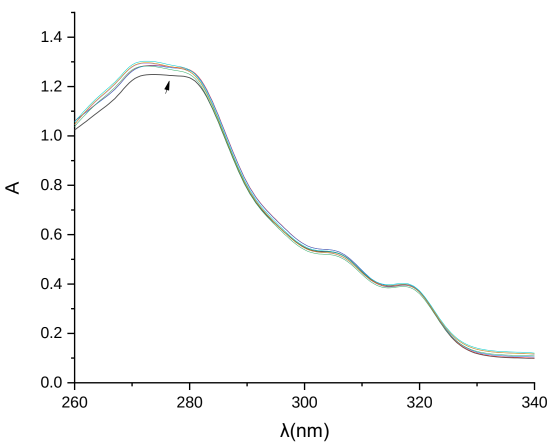

30

Figure S2.2. Plot of $\frac{[\mathrm{DNA}]}{(\varepsilon_A - \varepsilon_f)}$ *versus [DNA] of compounds 1–30*

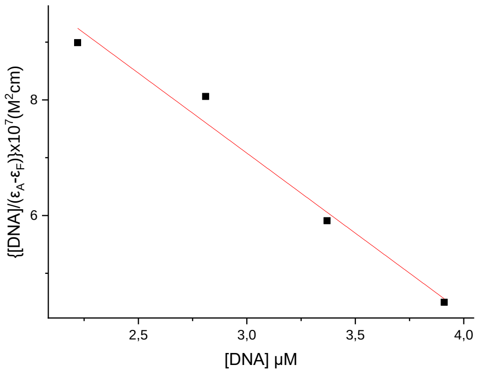

1

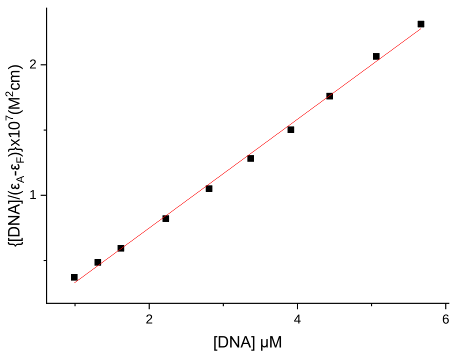

2

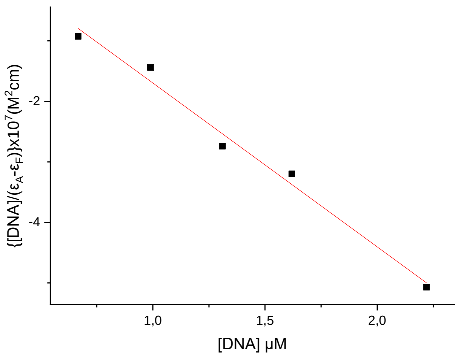

3

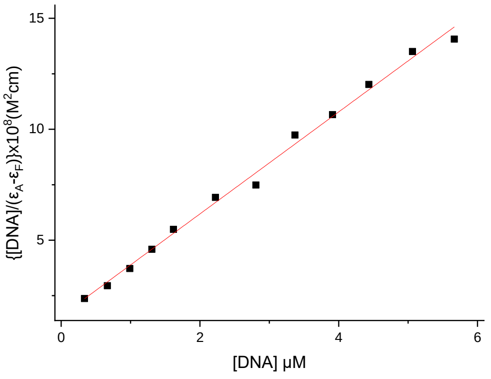

4

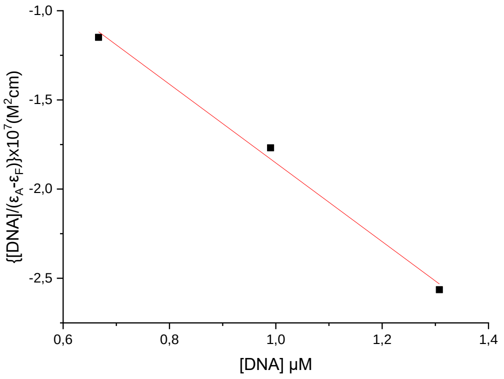

5

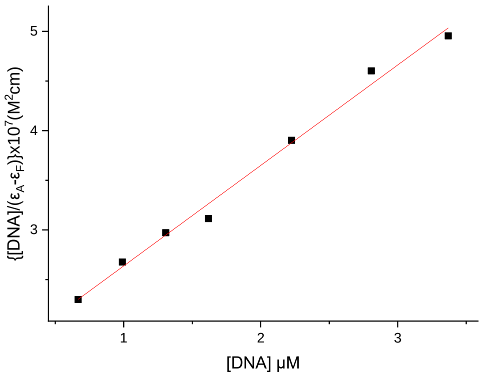

6

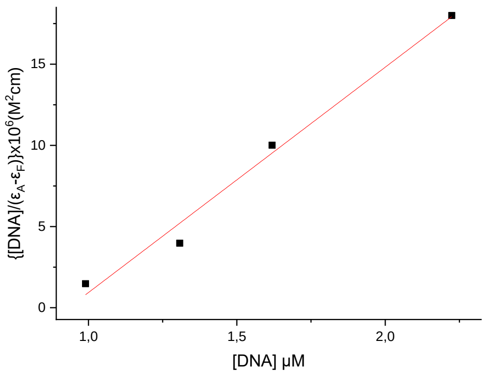

7

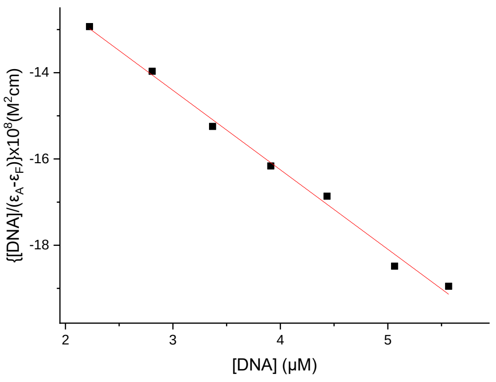

8

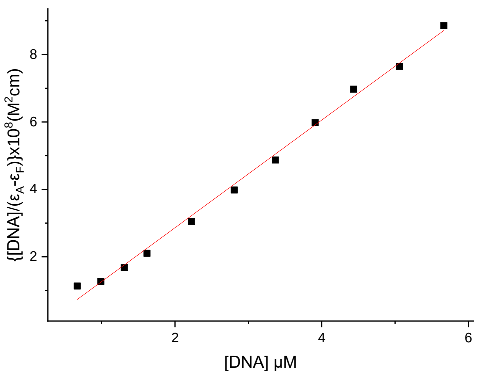

9

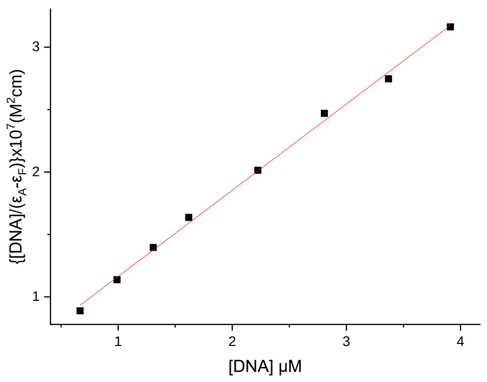

10

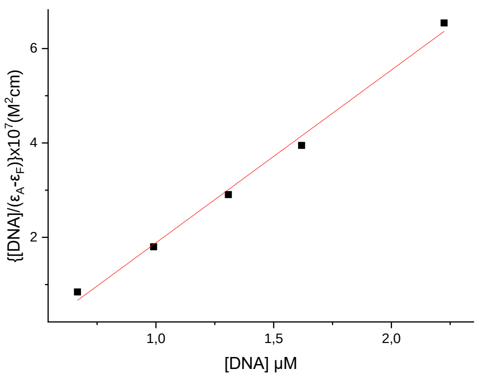

11

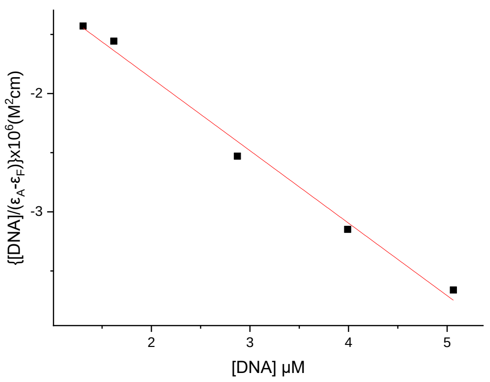

12

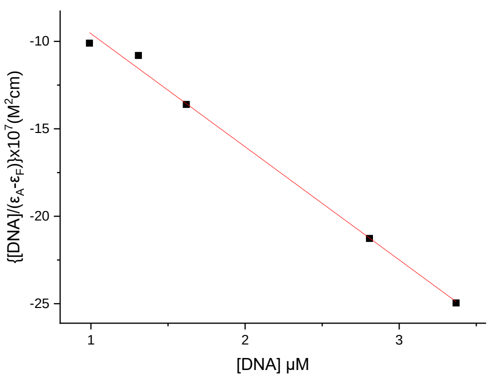

13

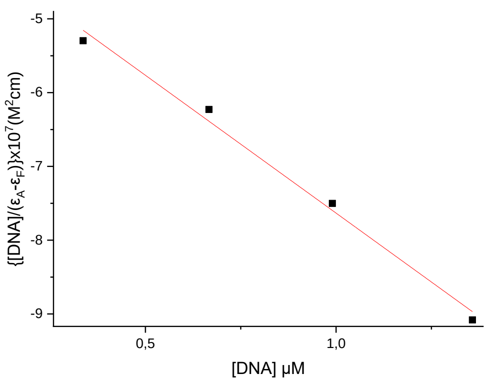

14

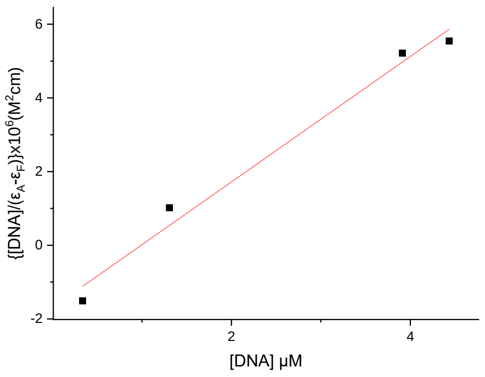

15

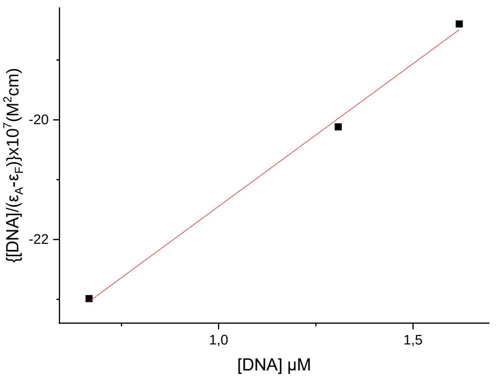

16

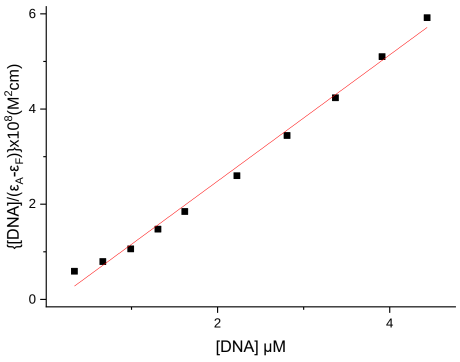

17

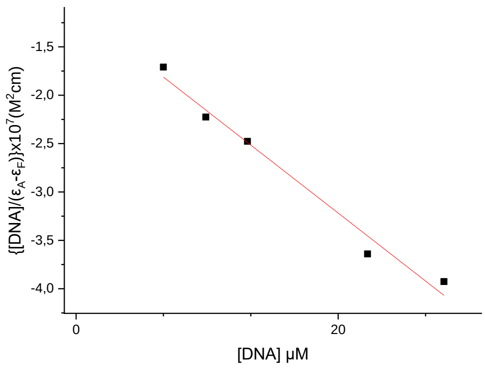

18

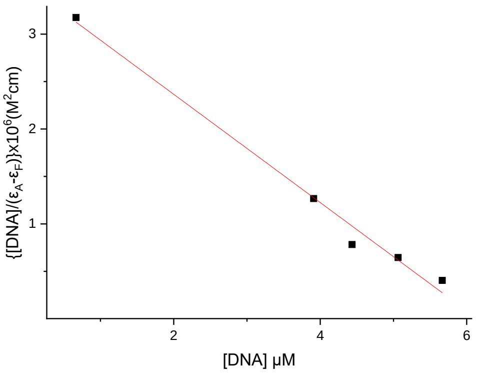

19

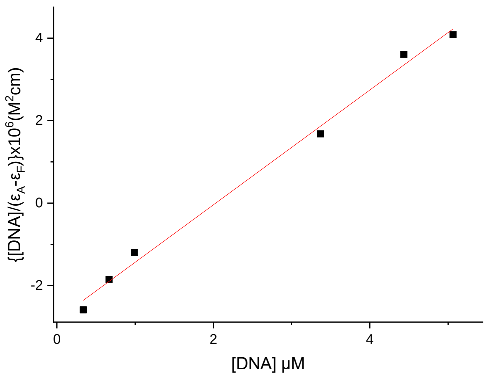

20

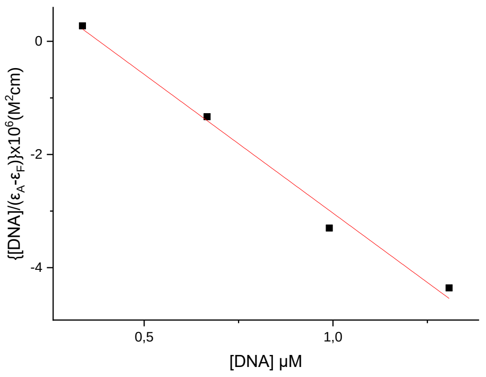

21

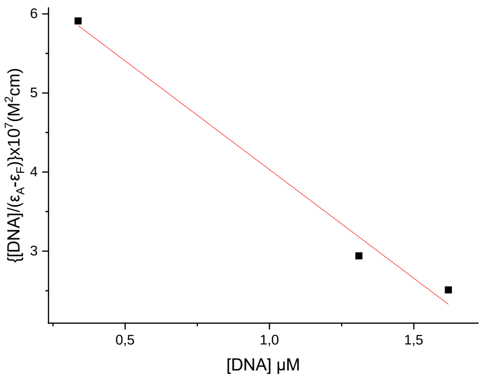

22

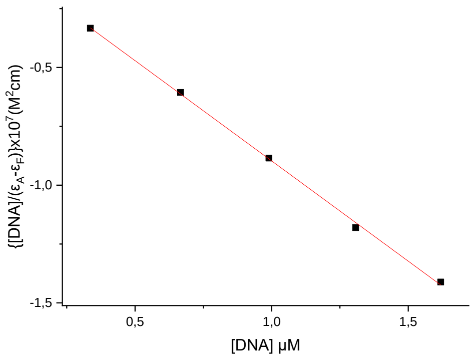

23

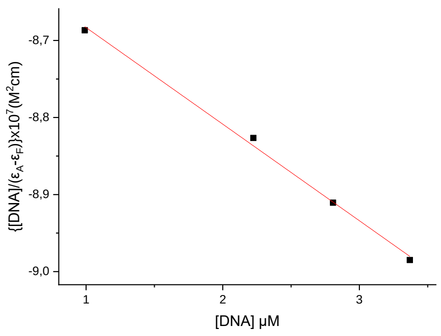

24

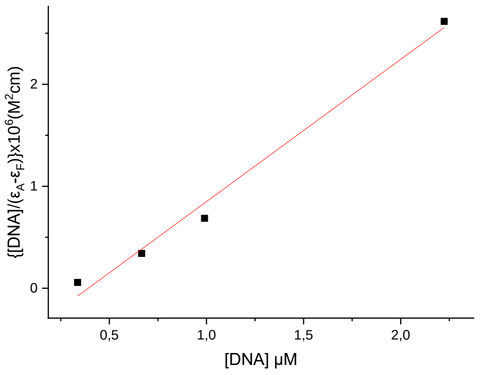

25

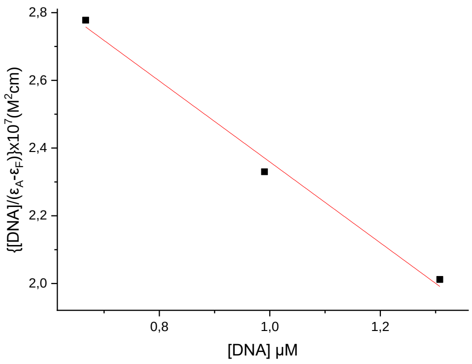

26

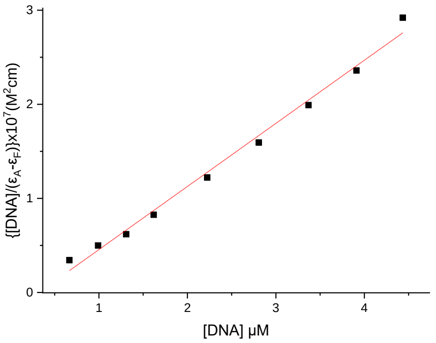

27

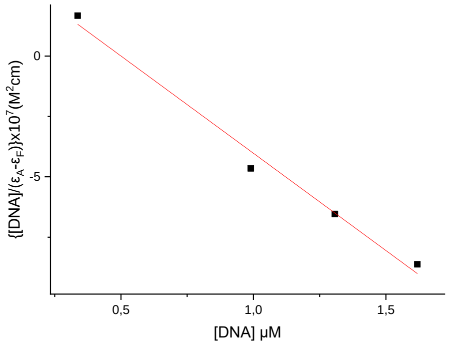

28

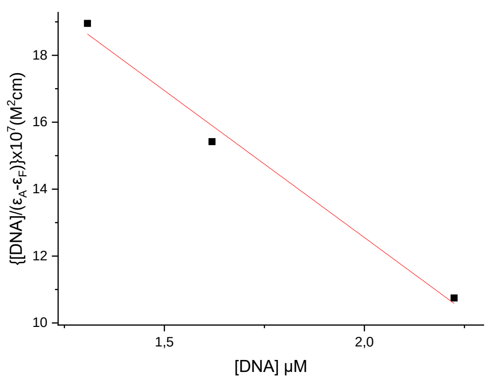

{[DNA]/(εA-εF)}x10^7(M^2cm)
10
12
14
16
18
1,5
2,0
[DNA] μM

29

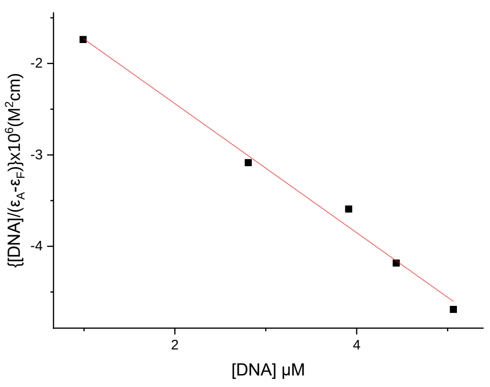

{[DNA]/(εA-εF)}x10^6(M^2cm)
-2
-3
-4
2
4
[DNA] μM

30

Figure S3.1. Fluorescence emission spectra (λ_{exc} = 540 nm) for EB-DNA conjugate ([EB] = 20 μM, [CT DNA] = 26 μM) in buffer solution (150 mM NaCl and 15 mM trisodium citrate at pH = 7.0) in the presence of increasing amounts of compounds **1–30**. The arrow shows the changes of intensity upon increasing amounts of the compounds

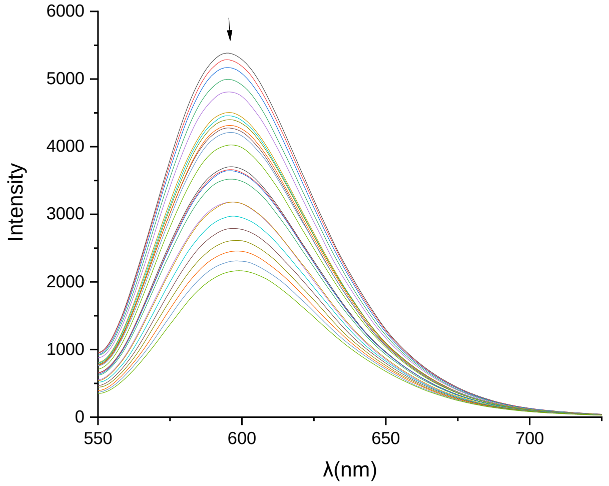

1

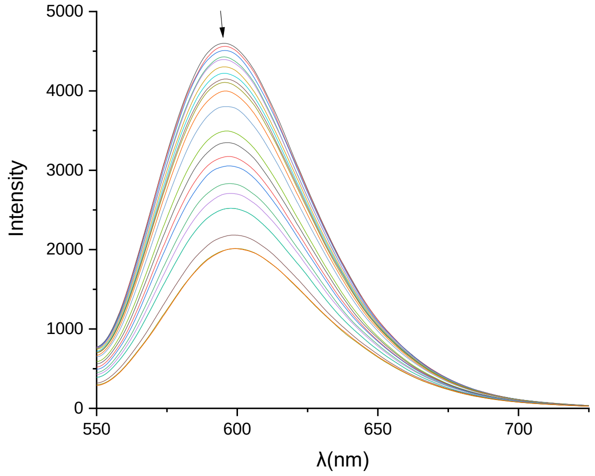

2

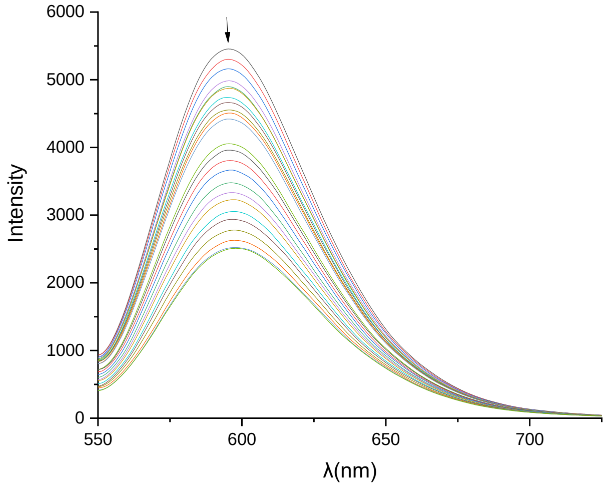

3

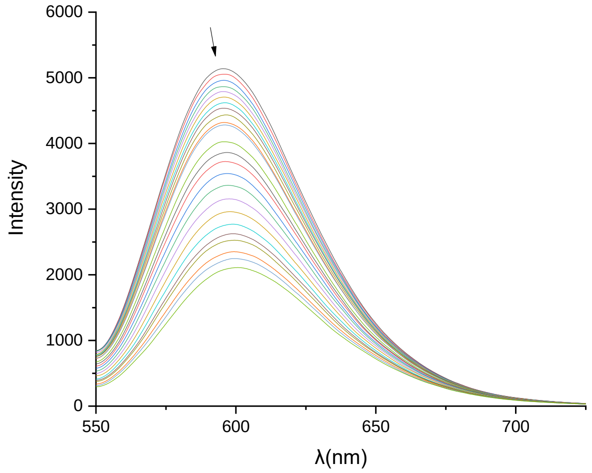

4

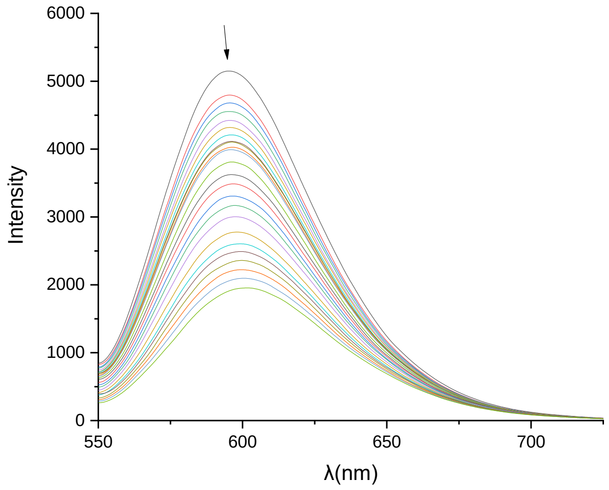
Intensity
λ(nm)
0
1000
2000
3000
4000
5000
6000
550
600
650
700

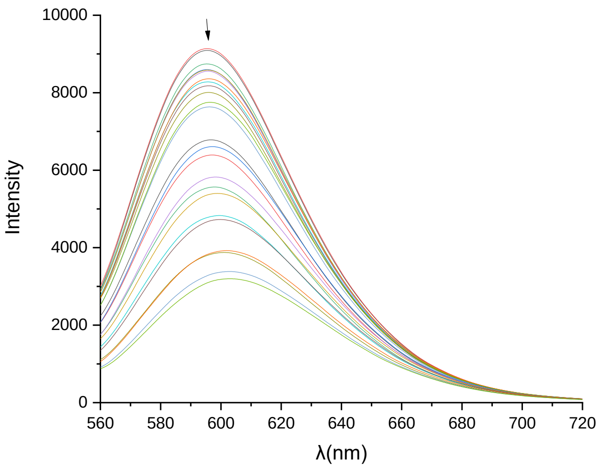
Intensity
λ(nm)
0
2000
4000
6000
8000
10000
560
580
600
620
640
660
680
700
720

5	6

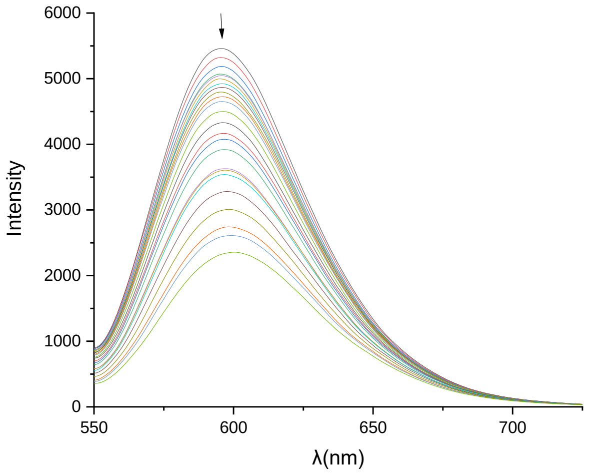
Intensity
λ(nm)
0
1000
2000
3000
4000
5000
6000
550
600
650
700

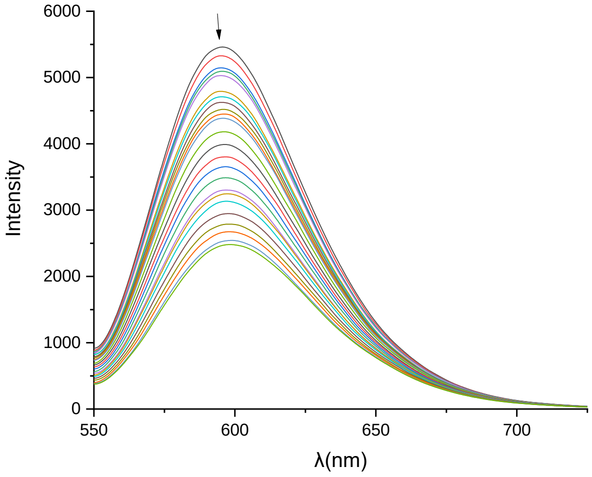
Intensity
λ(nm)
0
1000
2000
3000
4000
5000
6000
550
600
650
700

7	8

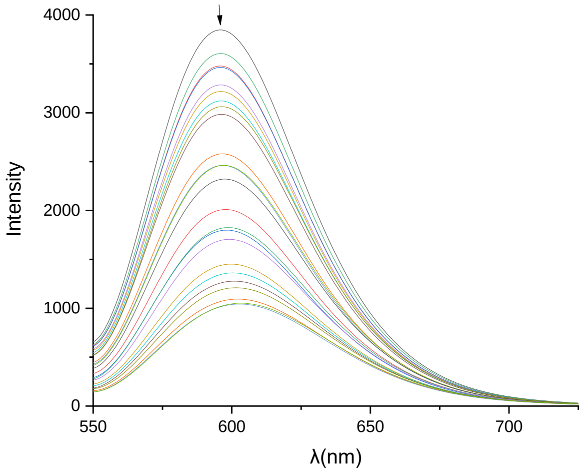
Intensity
λ(nm)
0
1000
2000
3000
4000
550
600
650
700

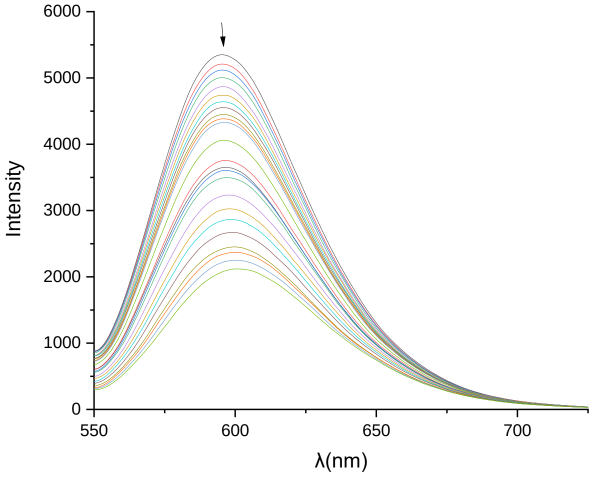
Intensity
λ(nm)
0
1000
2000
3000
4000
5000
6000
550
600
650
700

9	10
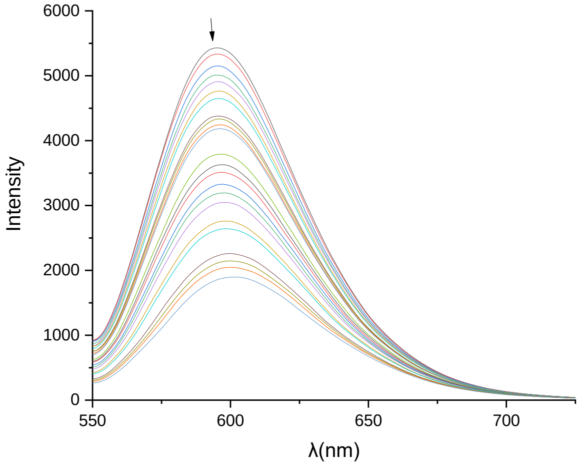	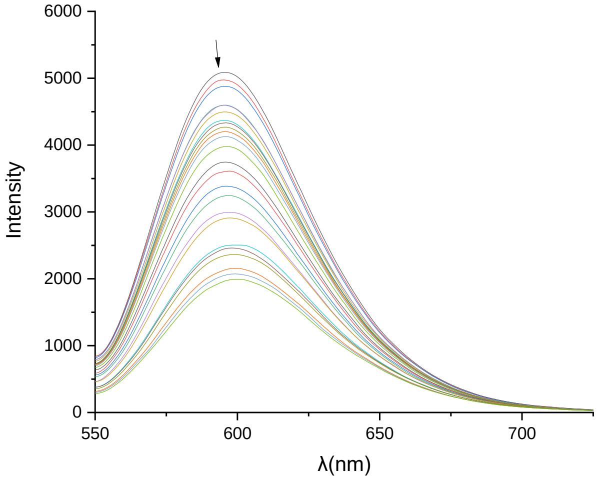
**11**	**12**
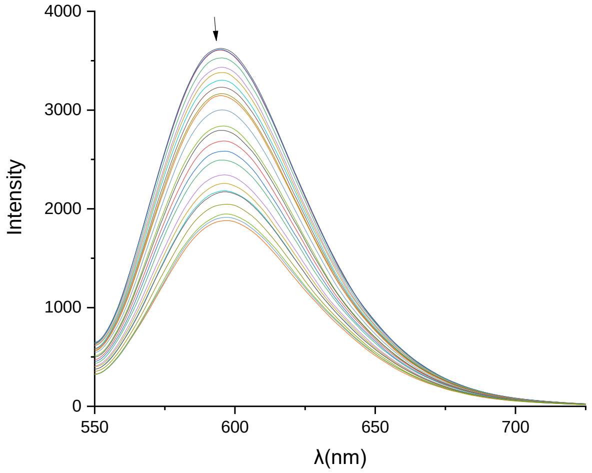	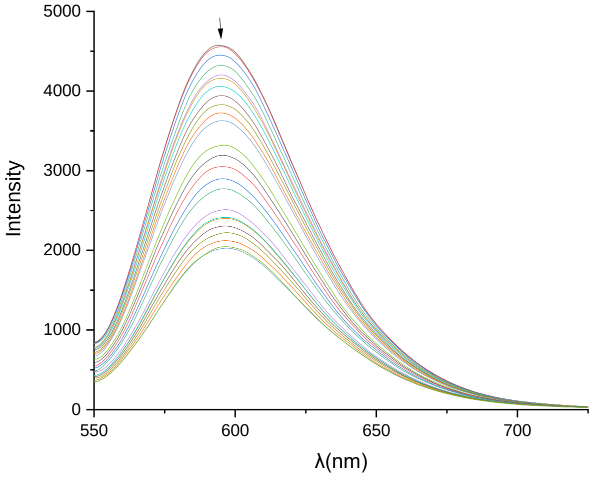
**13**	**14**
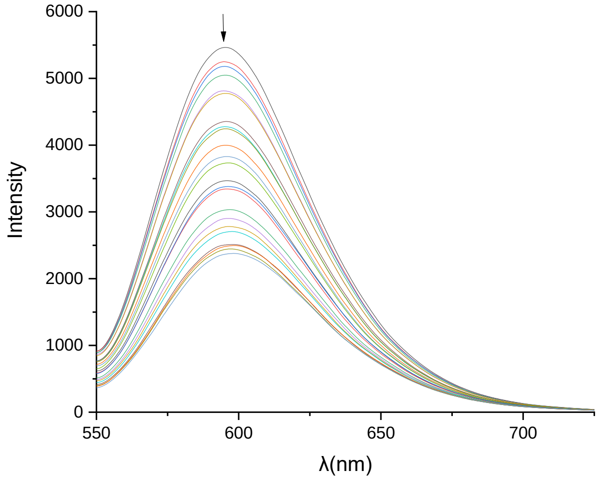	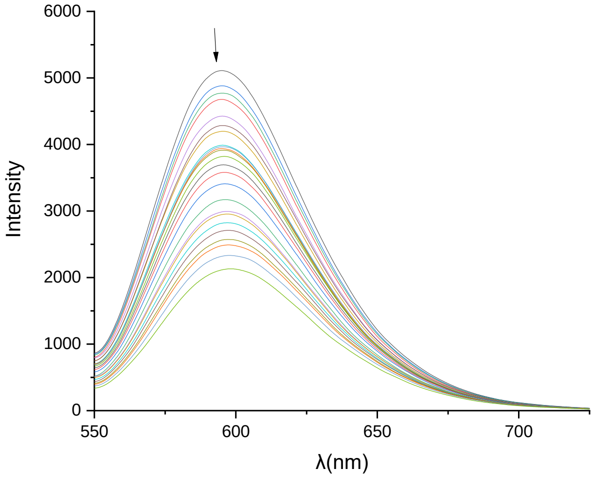

**15**	**16**
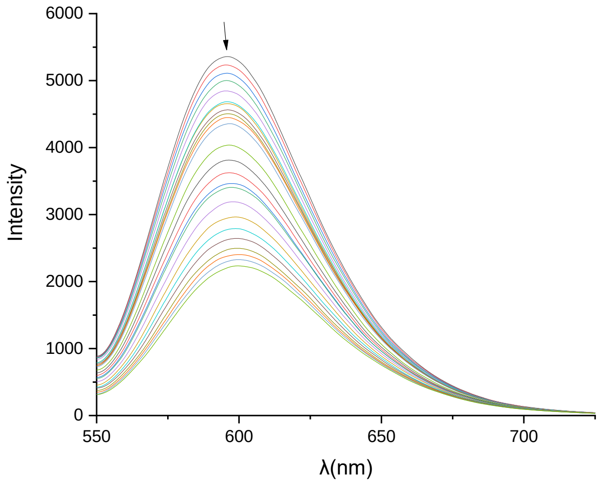	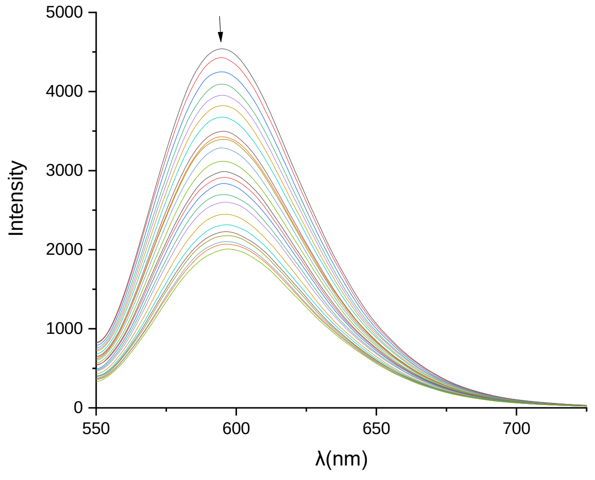
**17**	**18**
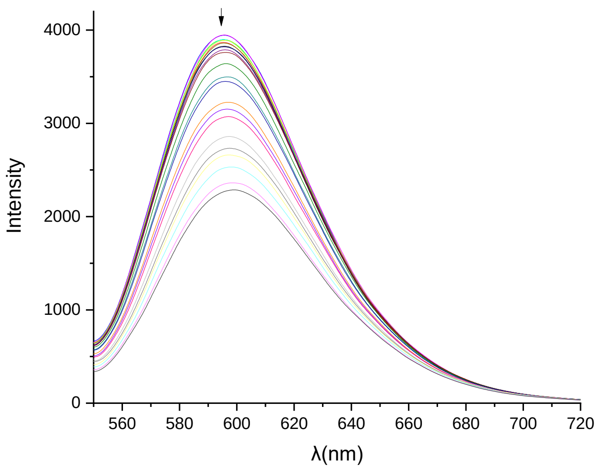	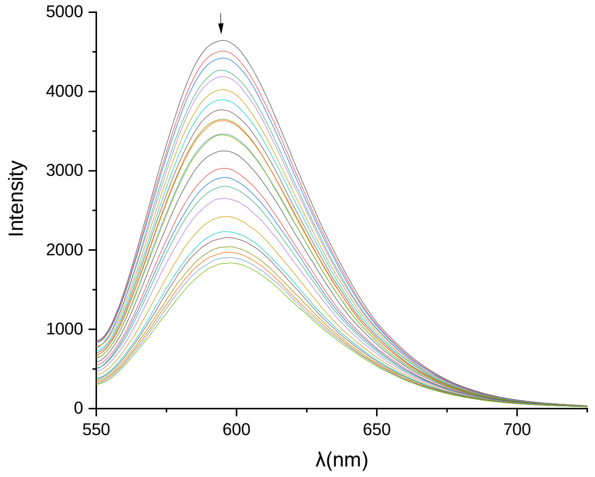
**19**	**20**

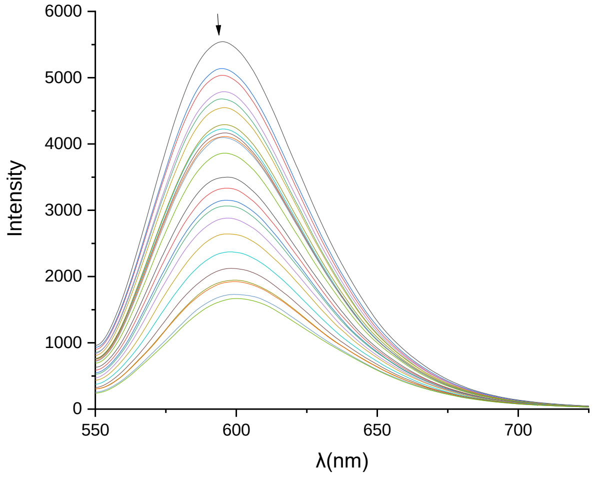

21

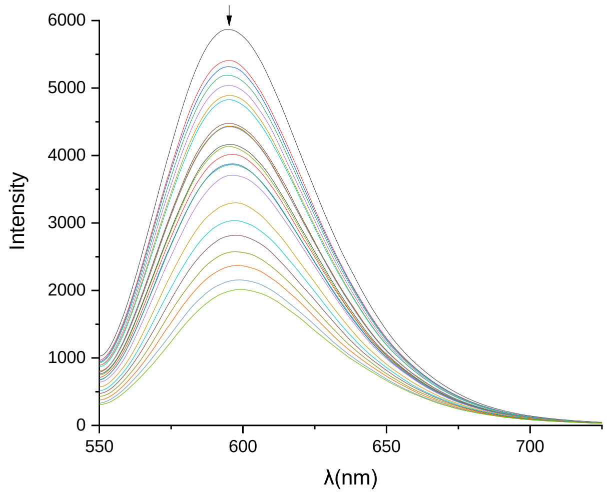

22

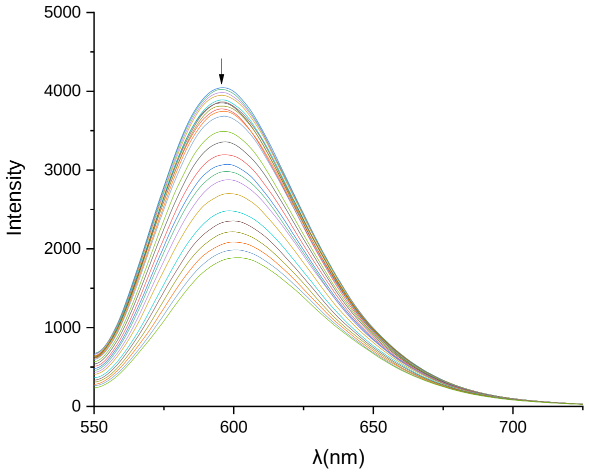

23

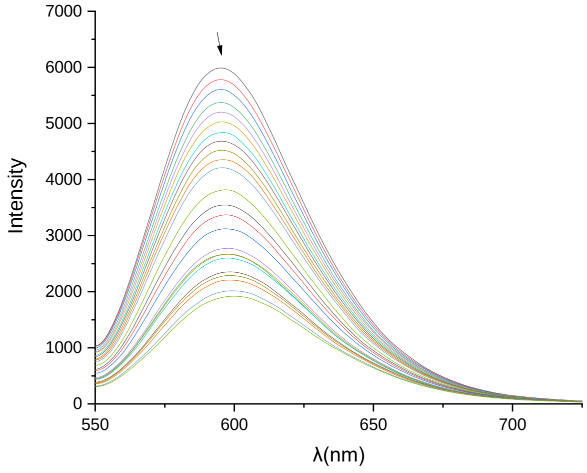

24

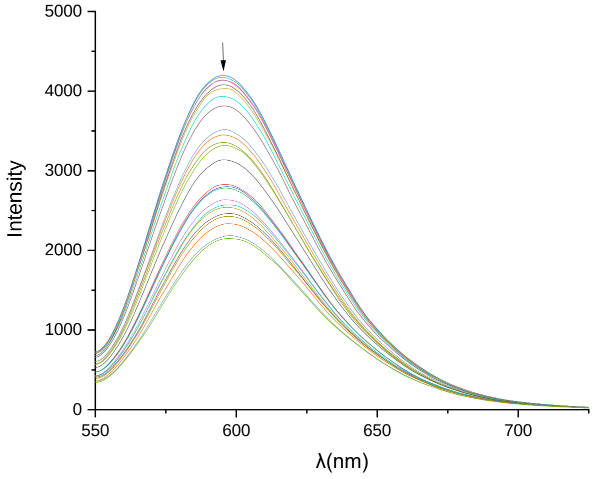

25

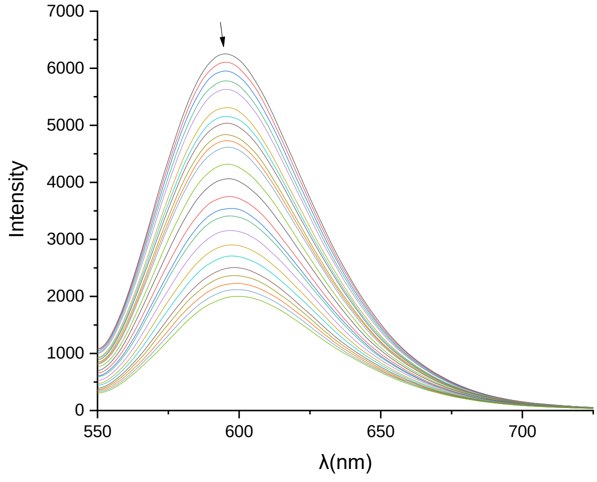

26

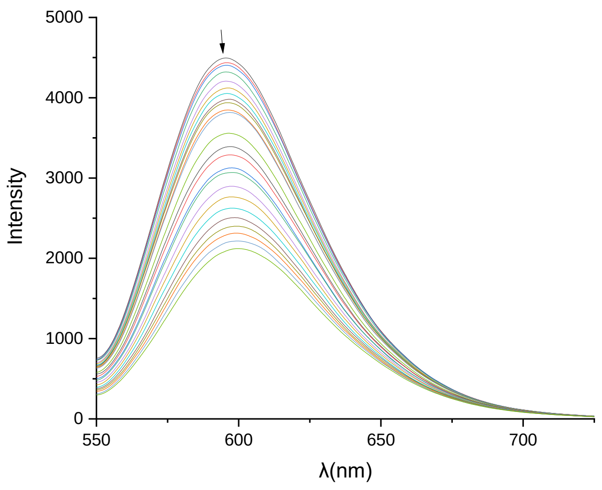
Intensity
λ(nm)
0
1000
2000
3000
4000
5000
550
600
650
700

27

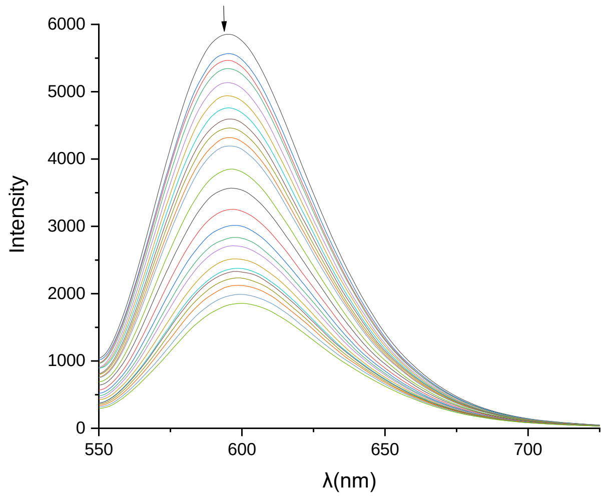
Intensity
λ(nm)
0
1000
2000
3000
4000
5000
6000
550
600
650
700

28

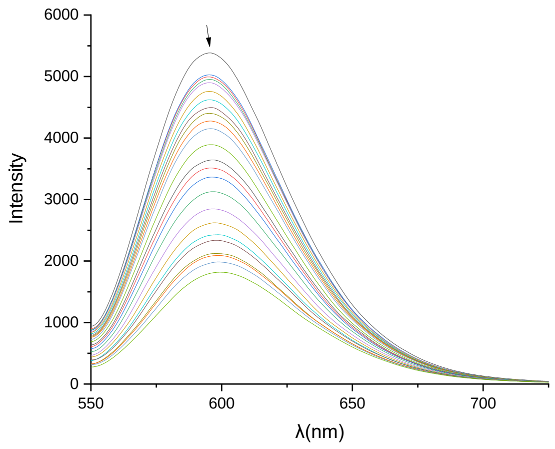
Intensity
λ(nm)
0
1000
2000
3000
4000
5000
6000
550
600
650
700

29

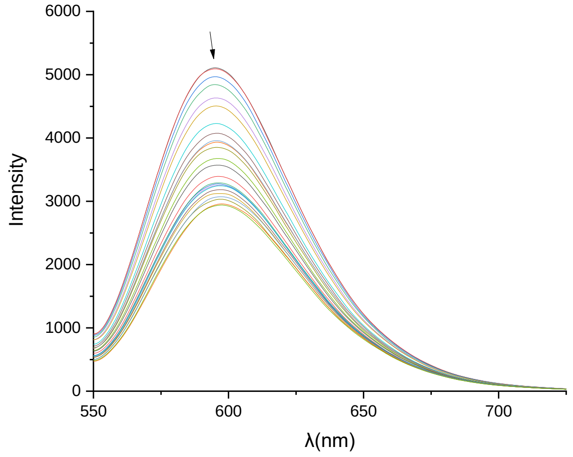
Intensity
λ(nm)
0
1000
2000
3000
4000
5000
6000
550
600
650
700

30

Figure S3.2. Stern–Volmer quenching plot of EB–DNA fluorescence of compounds 1–30

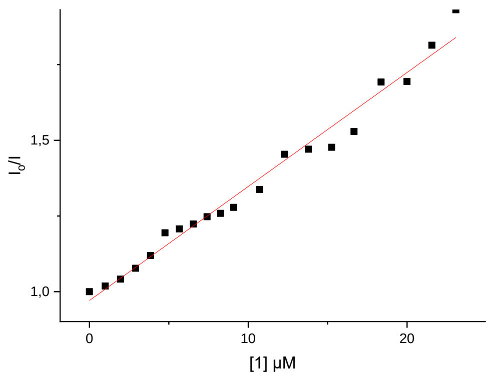

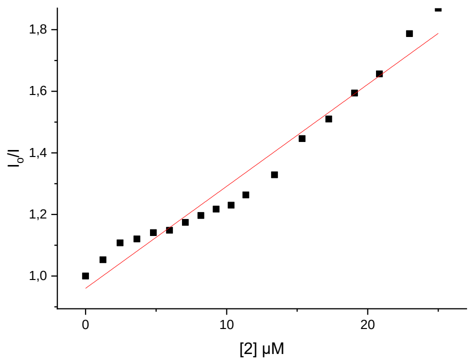

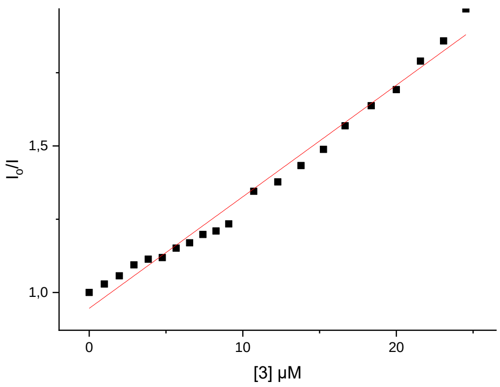

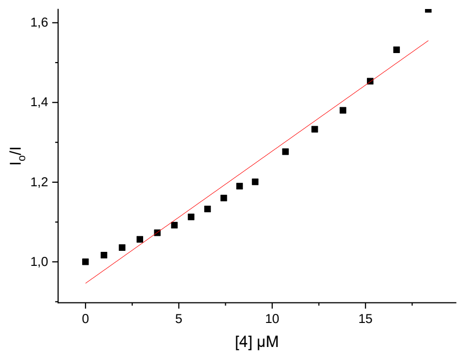

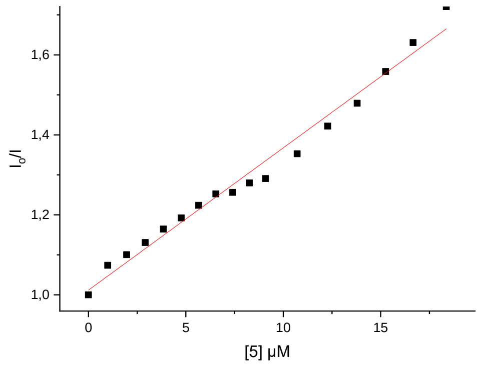

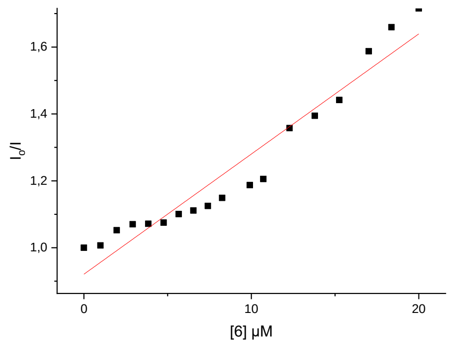

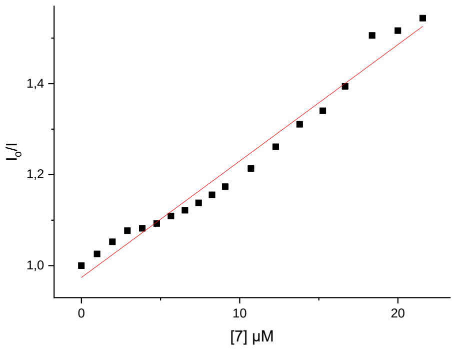

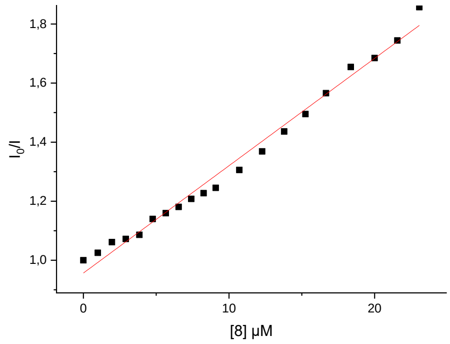

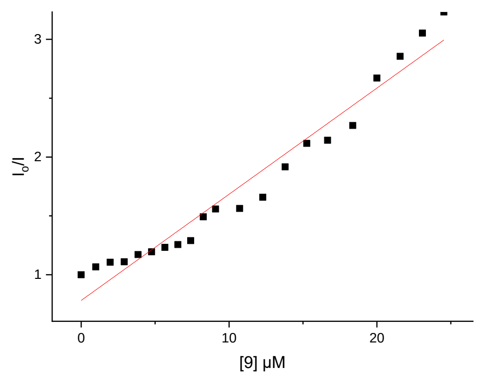
I_o/I
3
2
1
0
10
20
[9] µM

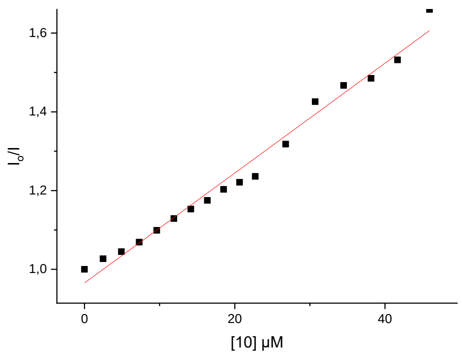
I_o/I
1,6
1,4
1,2
1,0
0
20
40
[10] µM

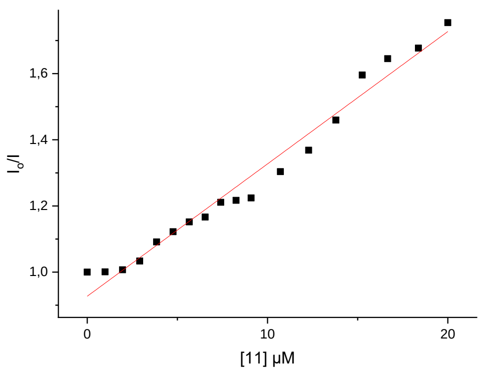
I_o/I
1,6
1,4
1,2
1,0
0
10
20
[11] µM

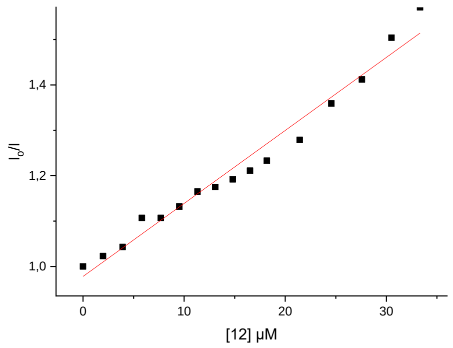
I_o/I
1,4
1,2
1,0
0
10
20
30
[12] µM

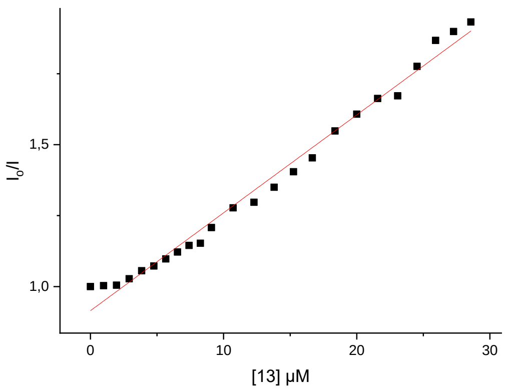
I_o/I
1,5
1,0
0
10
20
30
[13] µM

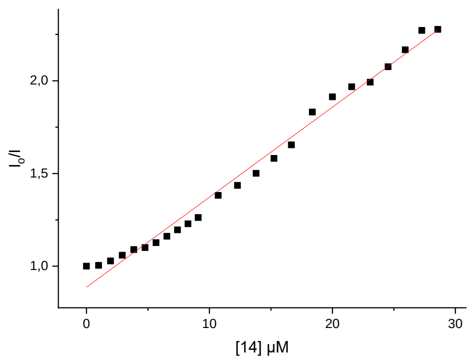
I_o/I
2,0
1,5
1,0
0
10
20
30
[14] µM

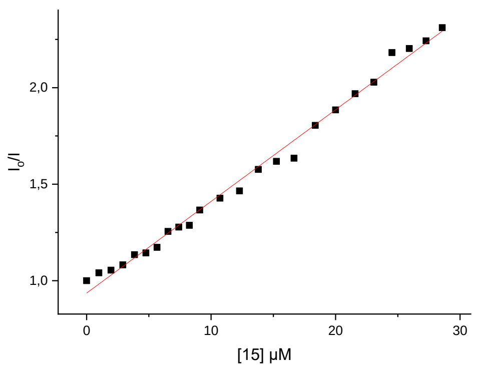
I_o/I
2,0
1,5
1,0
0
10
20
30
[15] µM

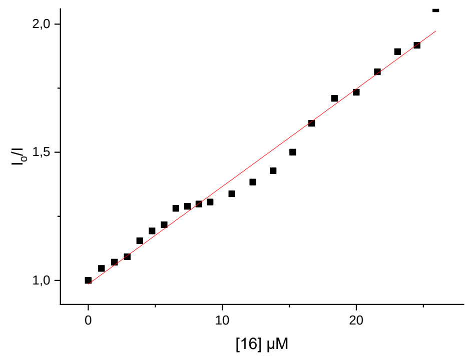
I_o/I
2,0
1,5
1,0
0
10
20
[16] µM

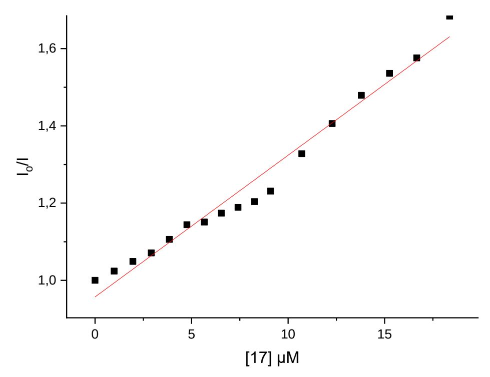

1,0
1,2
1,4
1,6
I_o/I
0
5
10
15
[17] µM

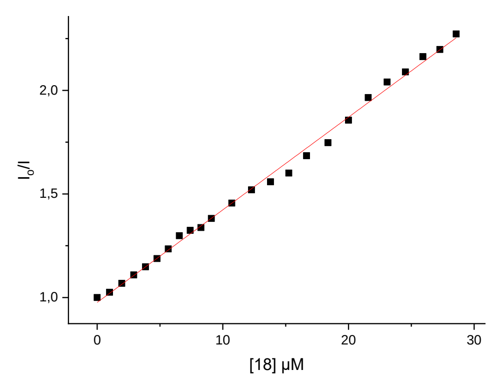

1,0
1,5
2,0
I_o/I
0
10
20
30
[18] µM

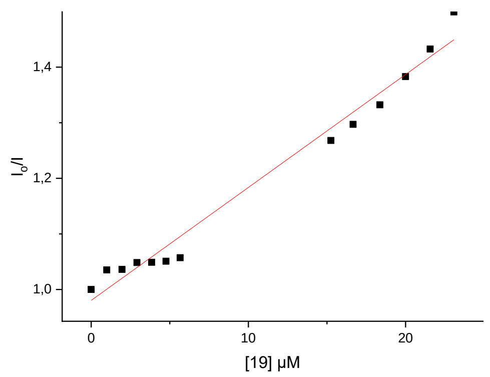

1,0
1,2
1,4
I_o/I
0
10
20
[19] µM

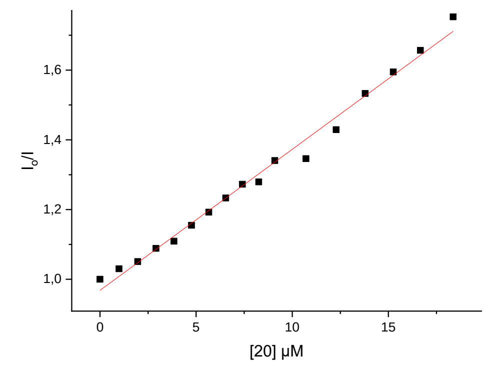

1,0
1,2
1,4
1,6
I_o/I
0
5
10
15
[20] µM

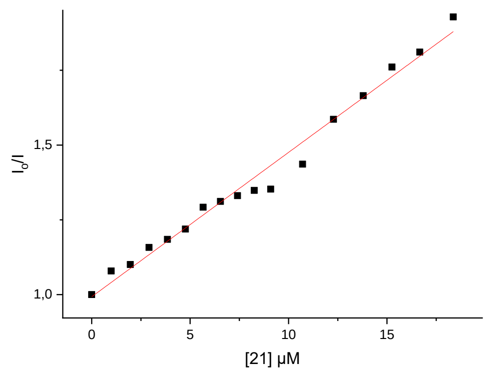

1,0
1,5
I_o/I
0
5
10
15
[21] µM

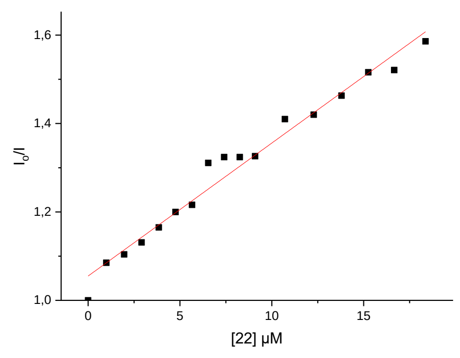

1,0
1,2
1,4
1,6
I_o/I
0
5
10
15
[22] µM

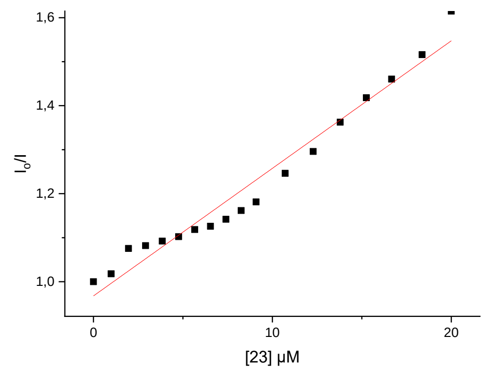

1,0
1,2
1,4
1,6
I_o/I
0
10
20
[23] µM

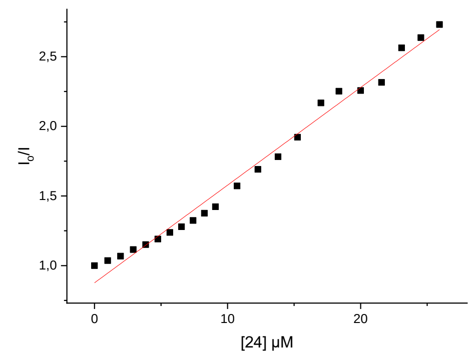

1,0
1,5
2,0
2,5
I_o/I
0
10
20
[24] µM

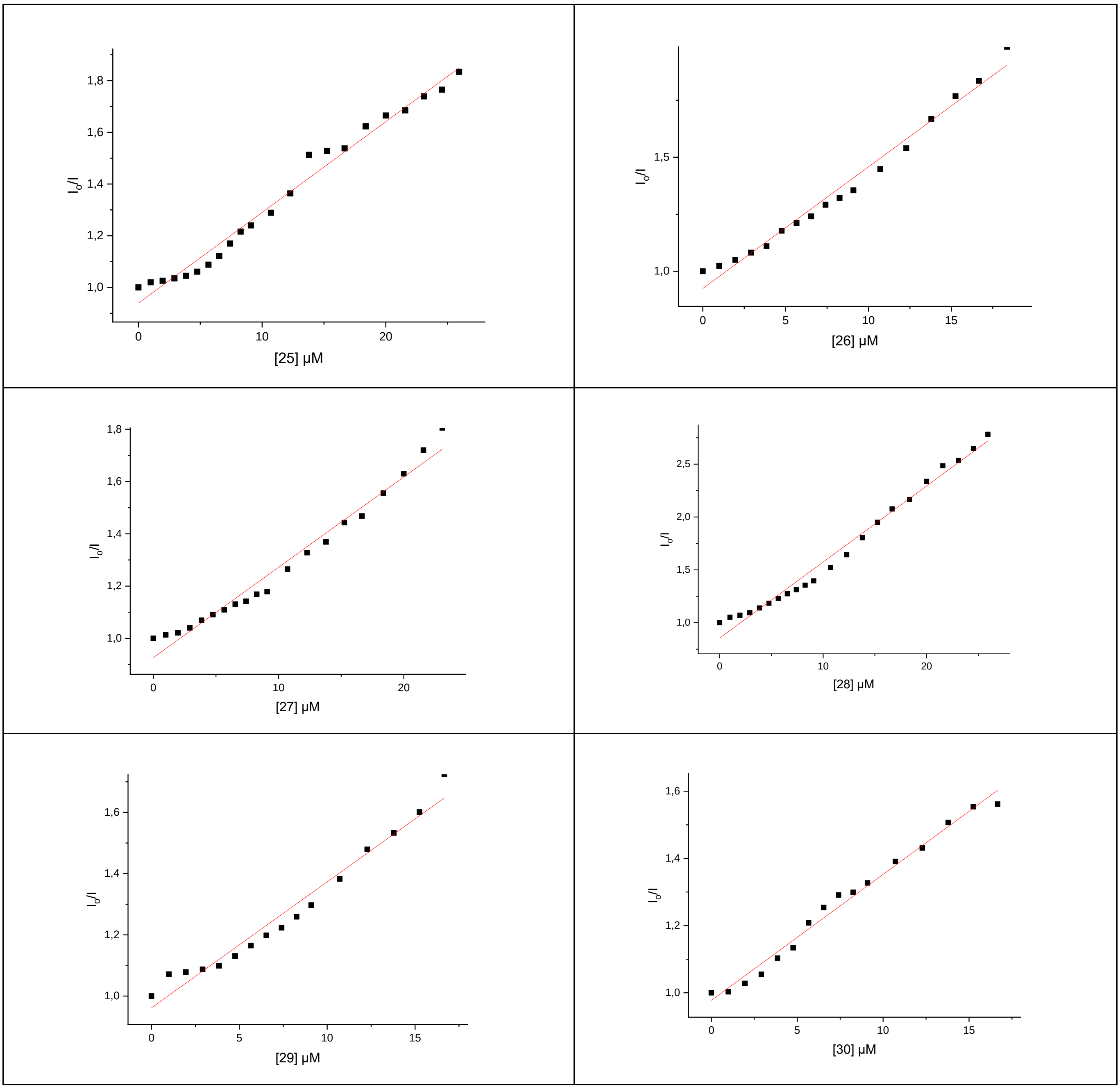

I_o/I
[25] μM
I_o/I
[26] μM
I_o/I
[27] μM
I_o/I
[28] μM
I_o/I
[29] μM
I_o/I
[30] μM

__Figure S4. UV-Vis spectra of compounds 1–30 in DMSO__

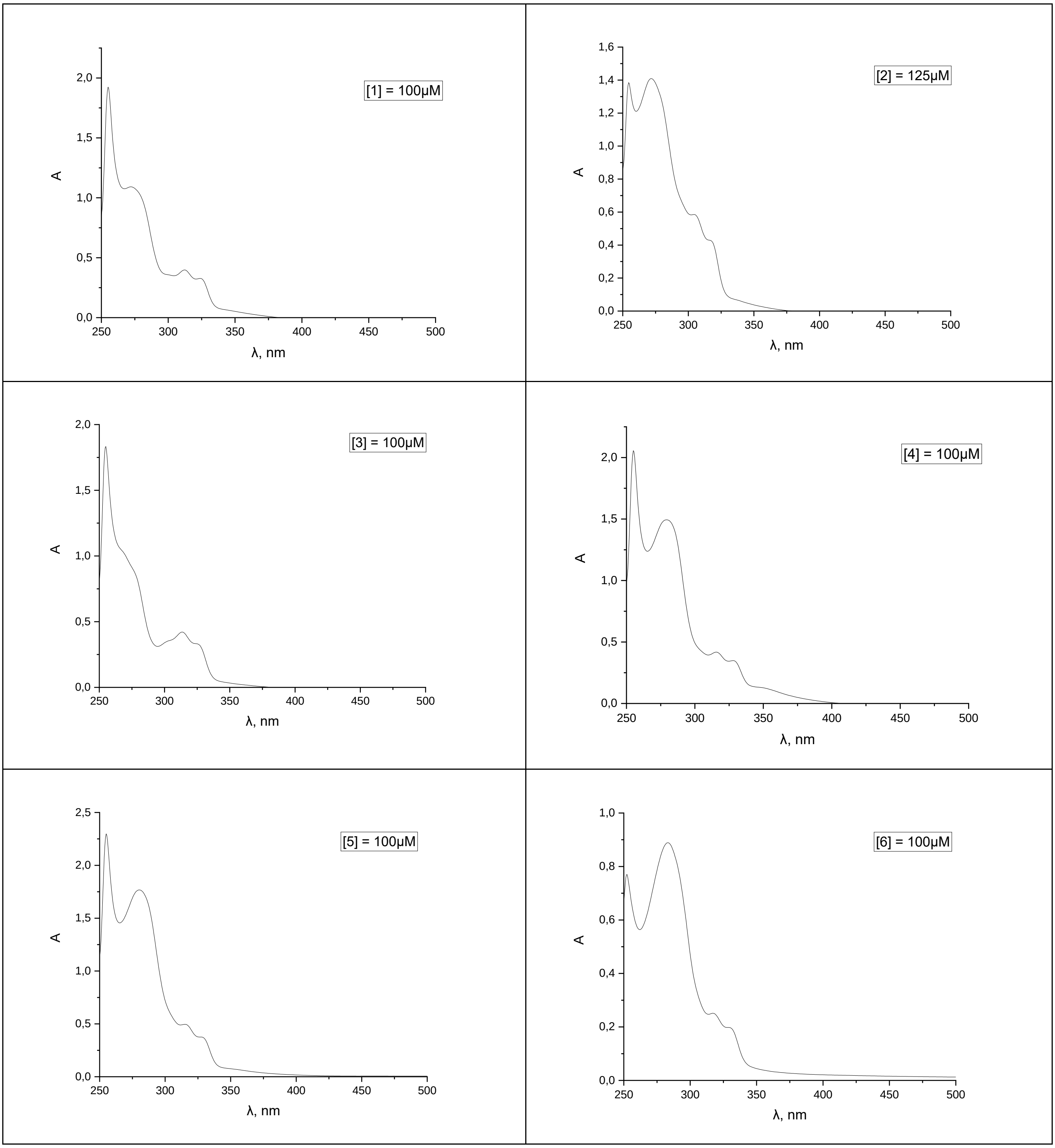

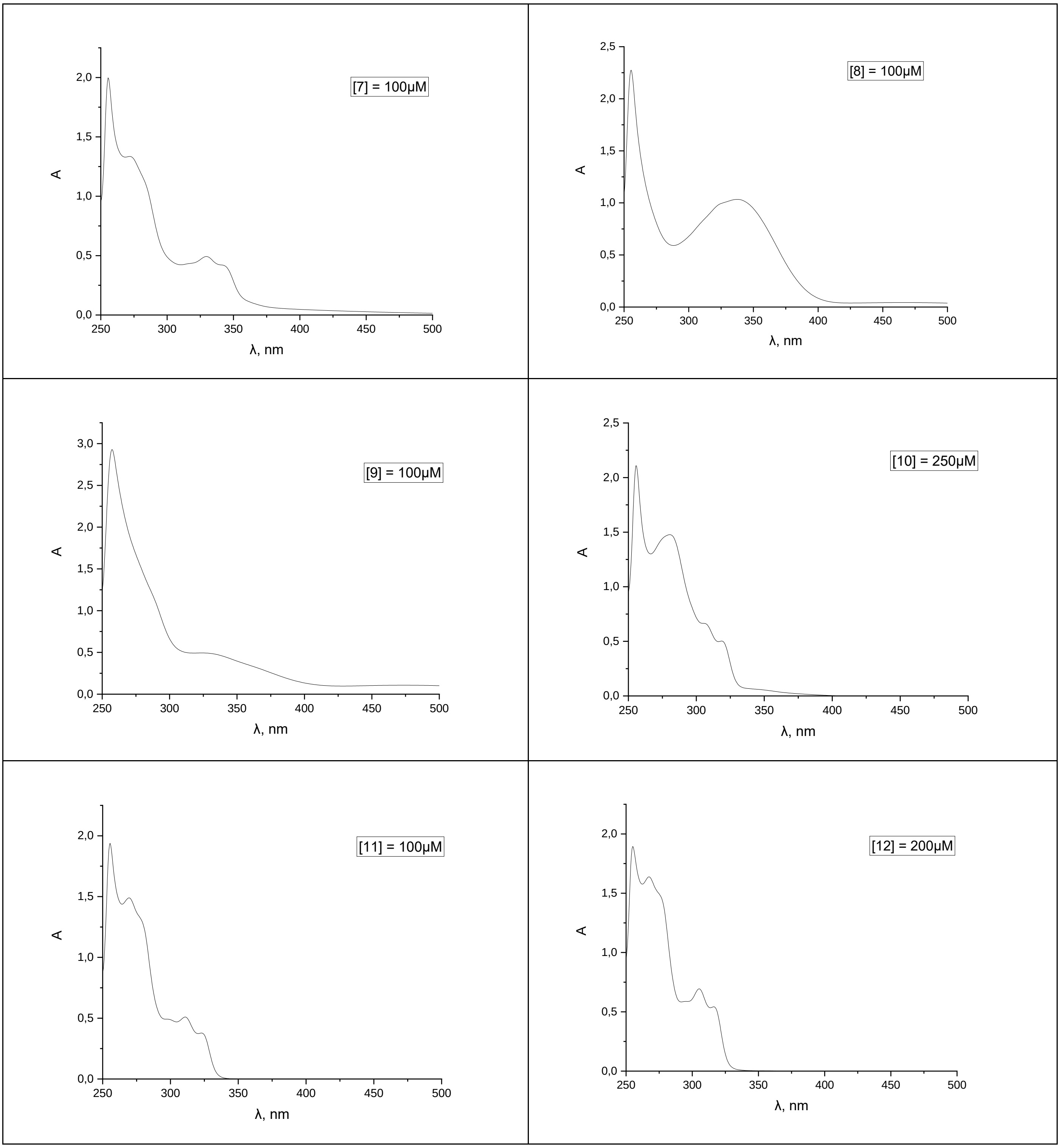
[7] = 100μM
[8] = 100μM
[9] = 100μM
[10] = 250μM
[11] = 100μM
[12] = 200μM
A
λ, nm

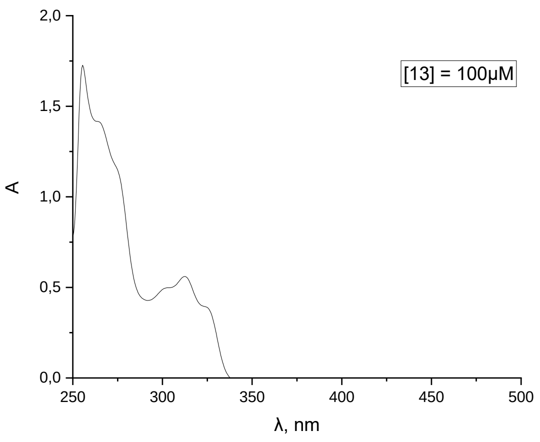
[13] = 100μM
A
λ, nm

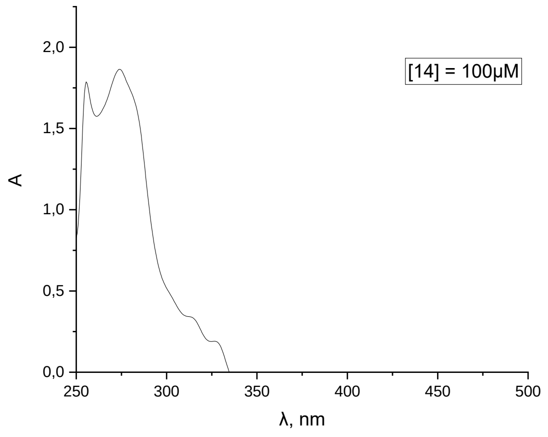
[14] = 100μM
A
λ, nm

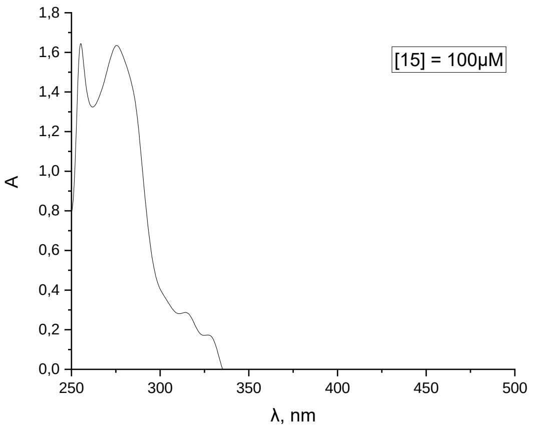
[15] = 100μM
A
λ, nm

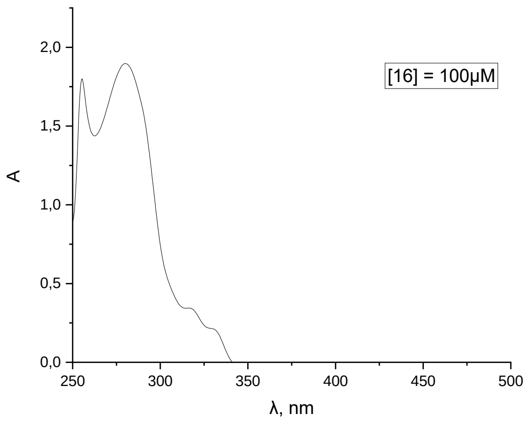
[16] = 100μM
A
λ, nm

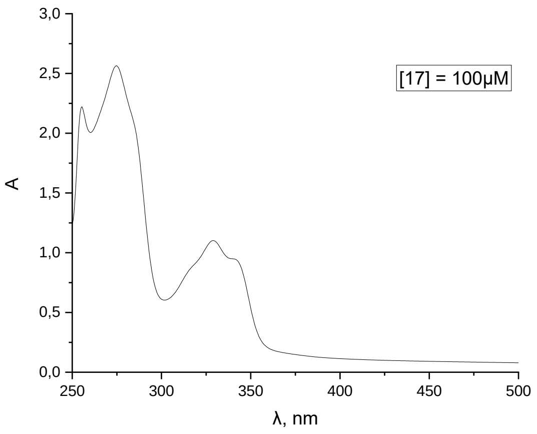
[17] = 100μM
A
λ, nm

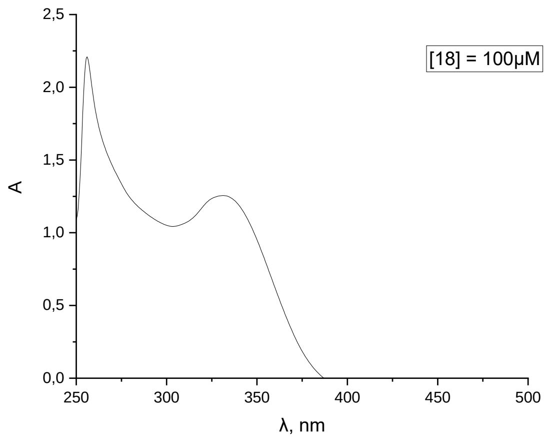
[18] = 100μM
A
λ, nm

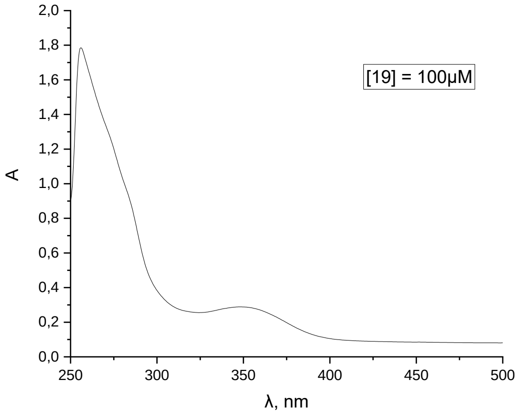
[19] = 100μM
A
λ, nm
2,0
1,8
1,6
1,4
1,2
1,0
0,8
0,6
0,4
0,2
0,0
250
300
350
400
450
500

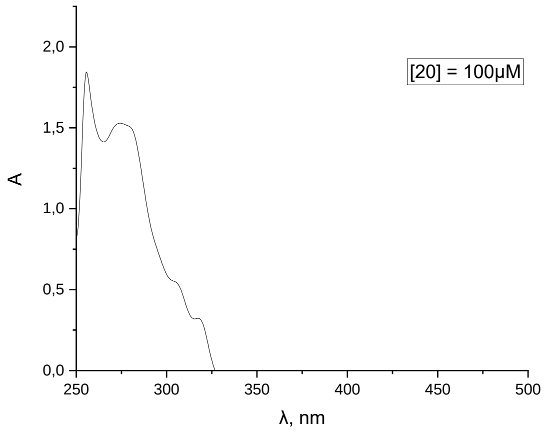
[20] = 100μM
A
λ, nm
2,0
1,5
1,0
0,5
0,0
250
300
350
400
450
500

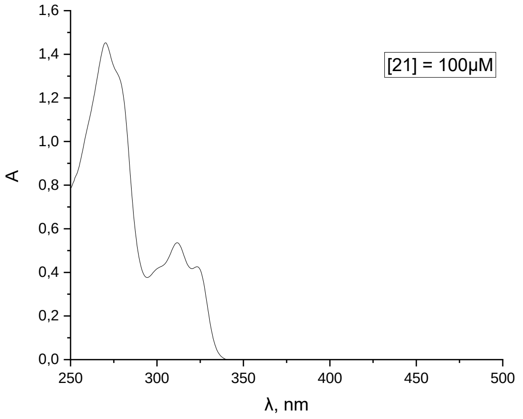
[21] = 100μM
A
λ, nm
1,6
1,4
1,2
1,0
0,8
0,6
0,4
0,2
0,0
250
300
350
400
450
500

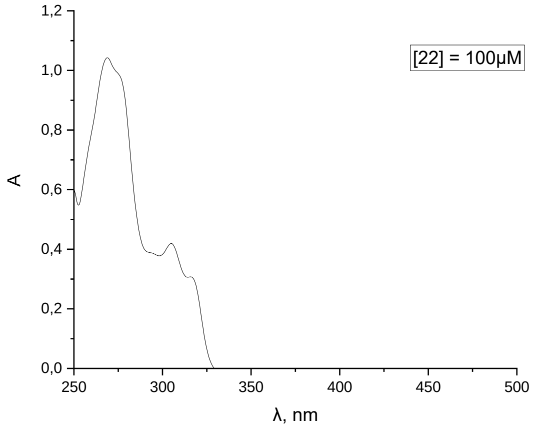
[22] = 100μM
A
λ, nm
1,2
1,0
0,8
0,6
0,4
0,2
0,0
250
300
350
400
450
500

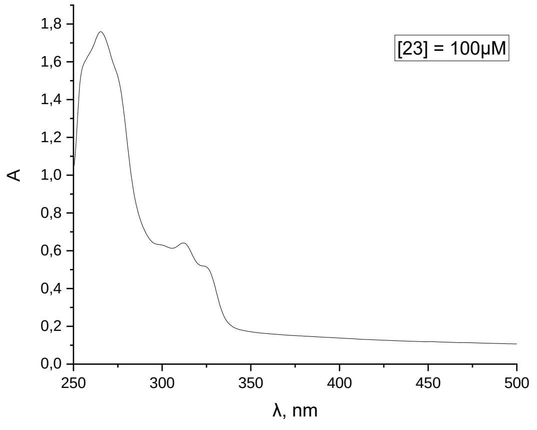
[23] = 100μM
A
λ, nm
1,8
1,6
1,4
1,2
1,0
0,8
0,6
0,4
0,2
0,0
250
300
350
400
450
500

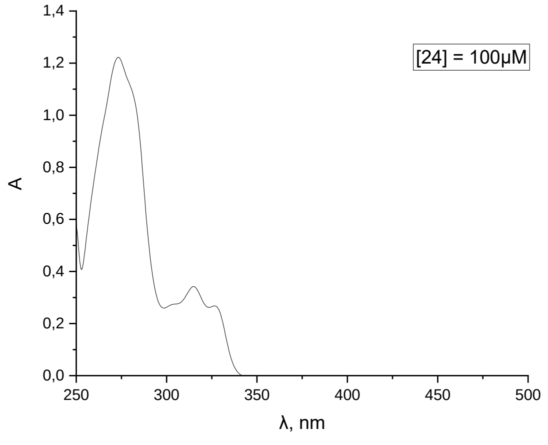
[24] = 100μM
A
λ, nm
1,4
1,2
1,0
0,8
0,6
0,4
0,2
0,0
250
300
350
400
450
500

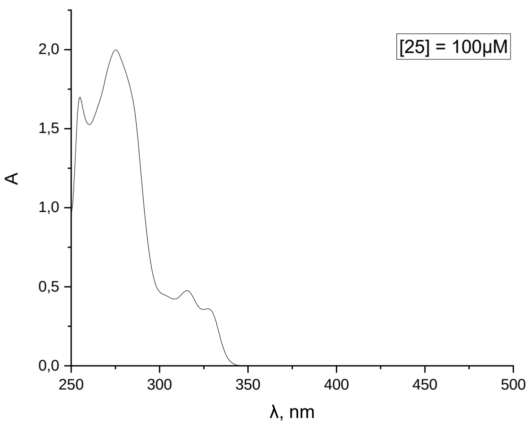
[25] = 100μM
A
λ, nm

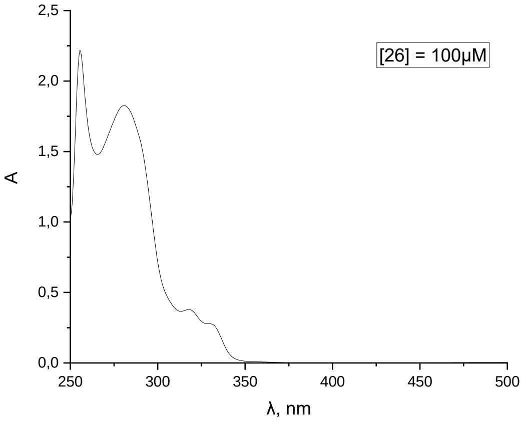
[26] = 100μM
A
λ, nm

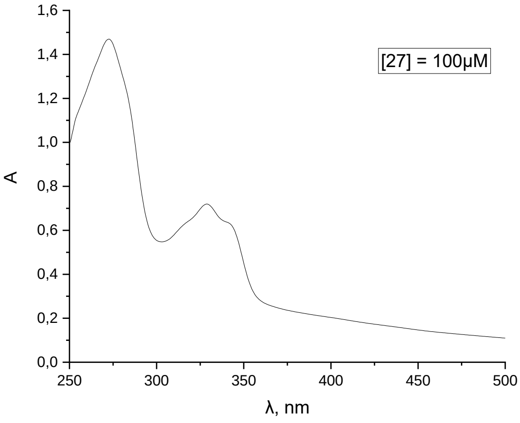
[27] = 100μM
A
λ, nm

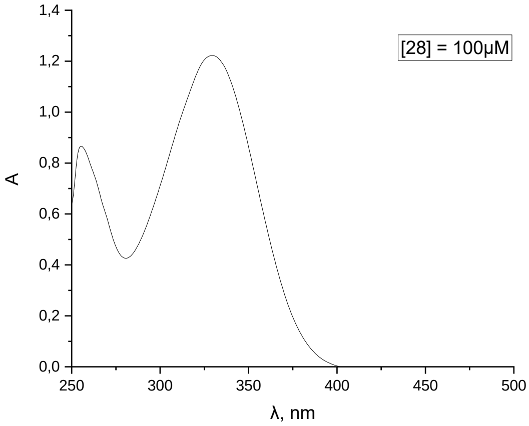
[28] = 100μM
A
λ, nm

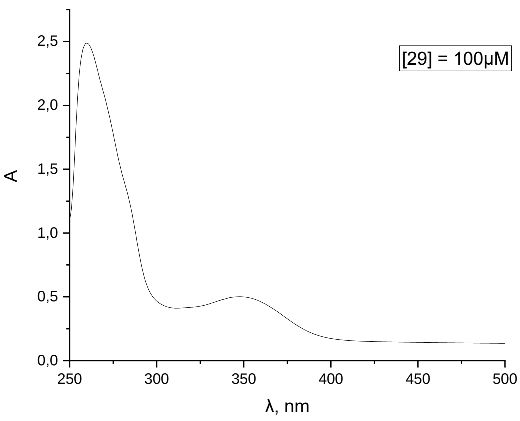
[29] = 100μM
A
λ, nm

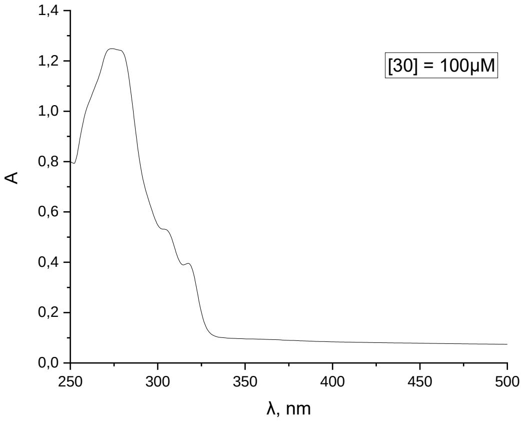
[30] = 100μM
A
λ, nm

S5. Gel electrophoresis pictures uncropped

Figure S5.1. *Agarose gel electrophoresis of nitro compounds **8, 9, 18, 19, 28, 29** in dark (triplicate, 100 μM)*

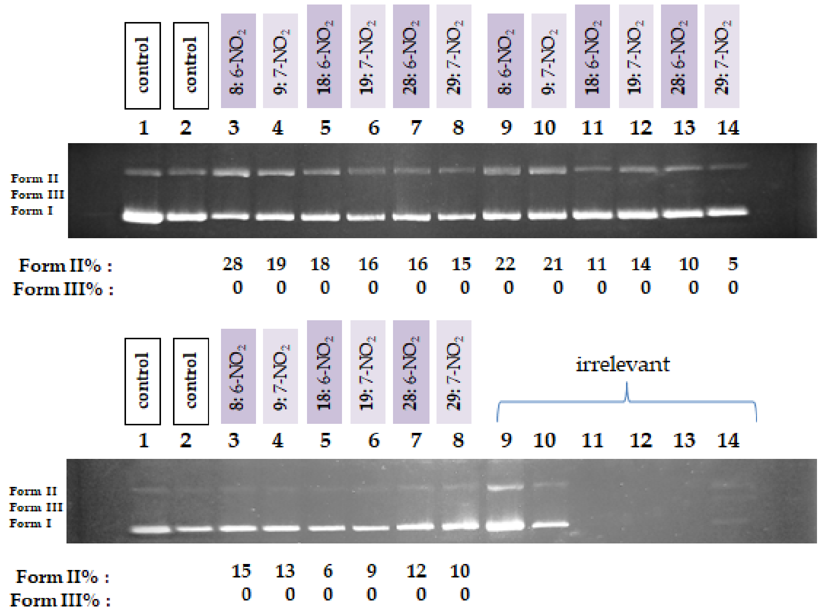

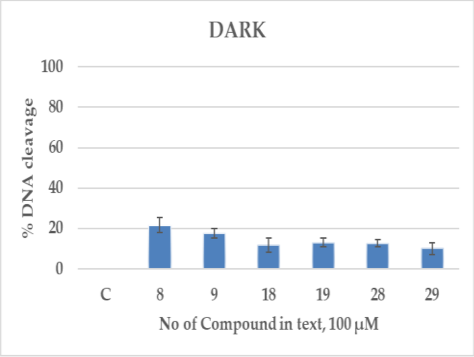

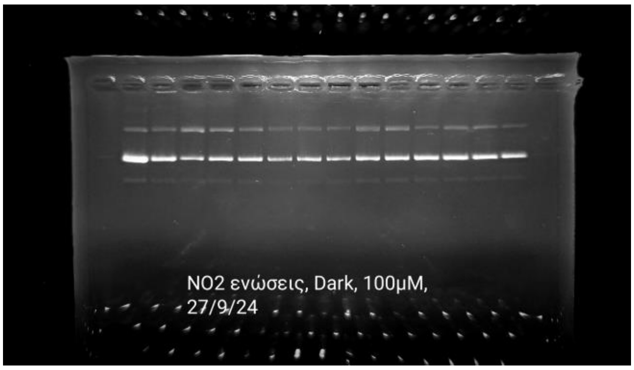

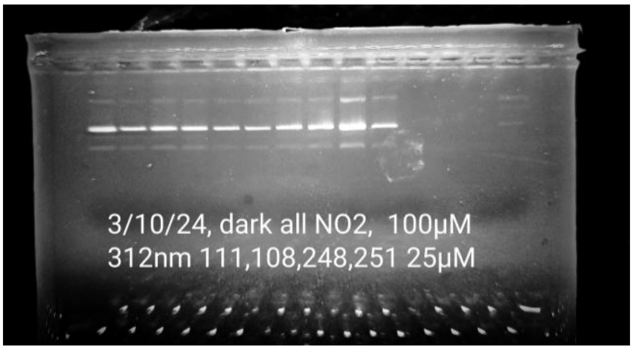

Figure S5.2. *Agarose gel electrophoresis of compounds **1–10** upon irradiation at 365 nm (triplicate, 100 µM)*

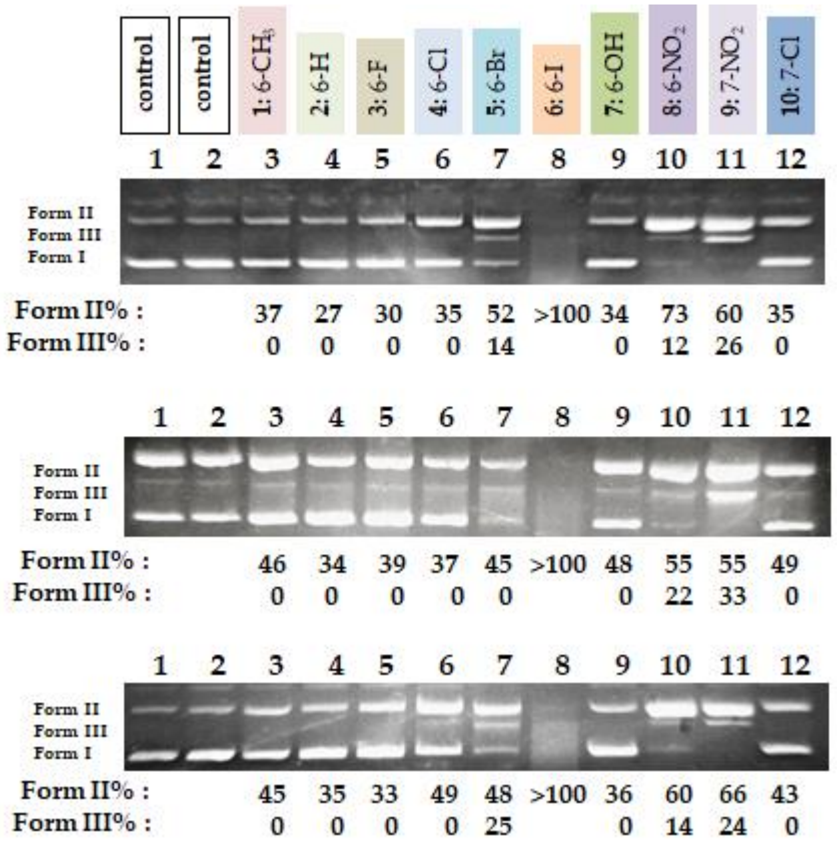

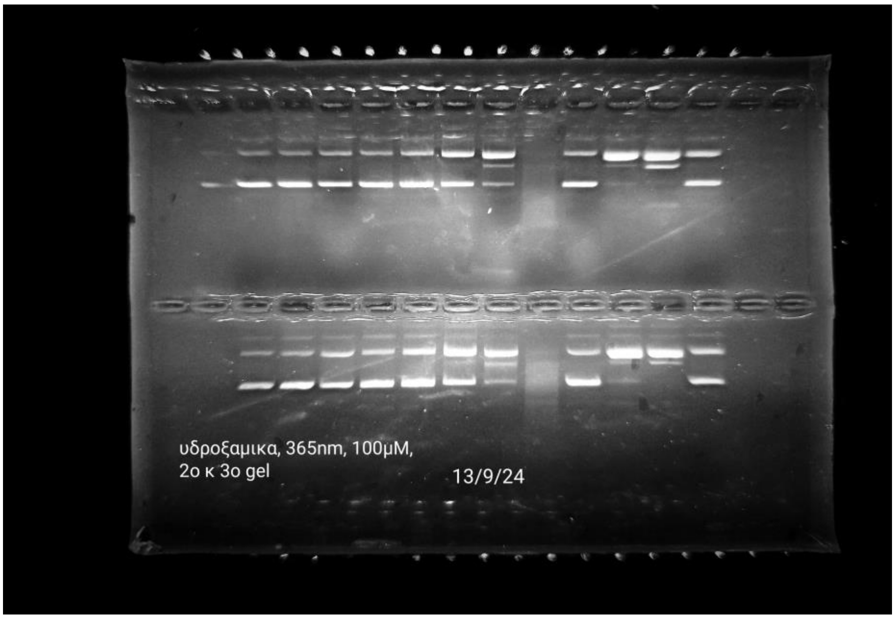

Figure S5.3. *Agarose gel electrophoresis of compounds* ***11–20*** *upon irradiation at 365 nm (triplicate, 100 μM)*

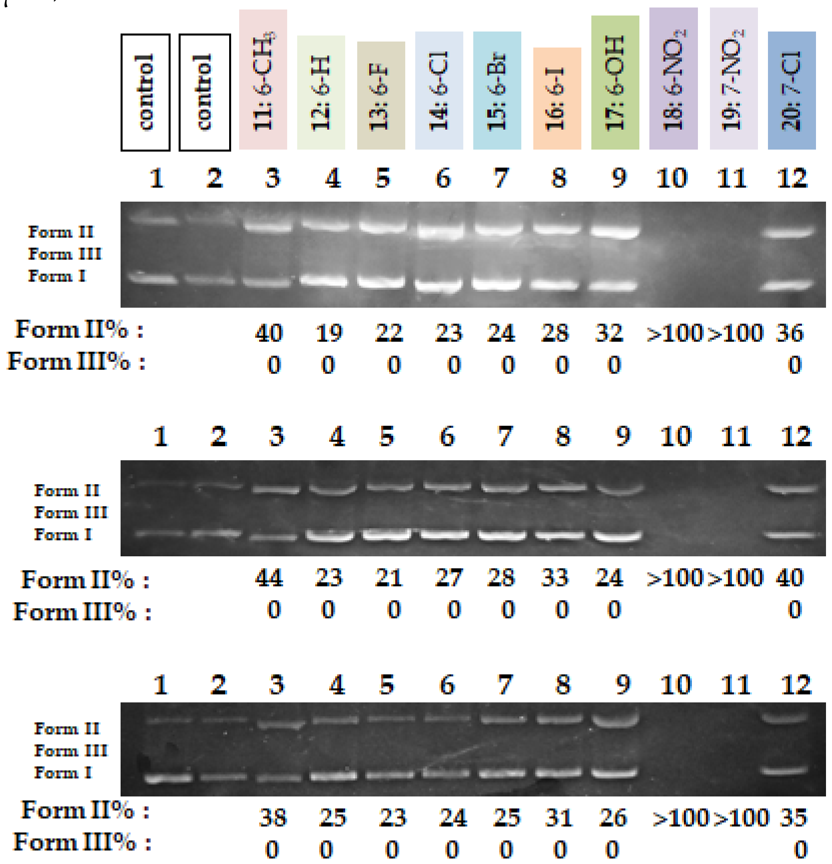

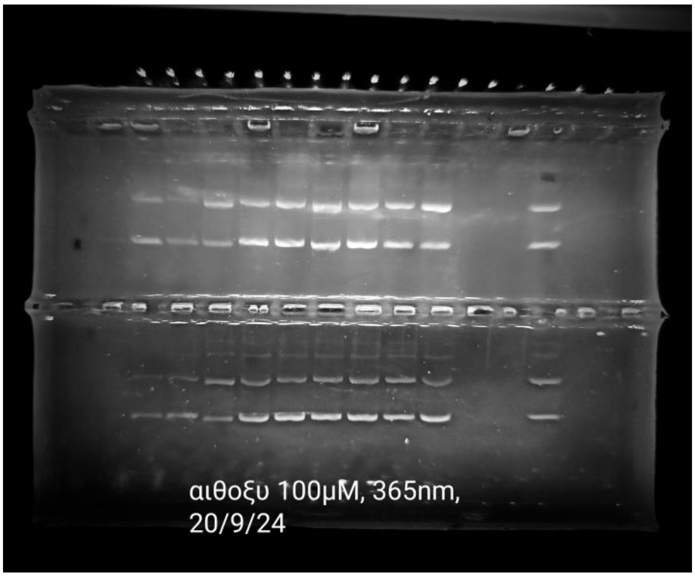

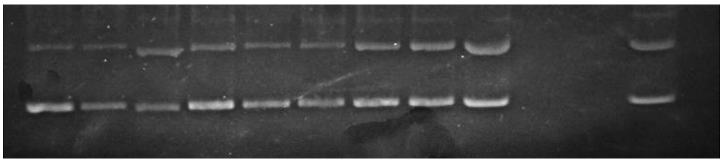

Figure S5.4. *Agarose gel electrophoresis of compounds **21–30** upon irradiation at 365 nm (triplicate, 100 μM)*

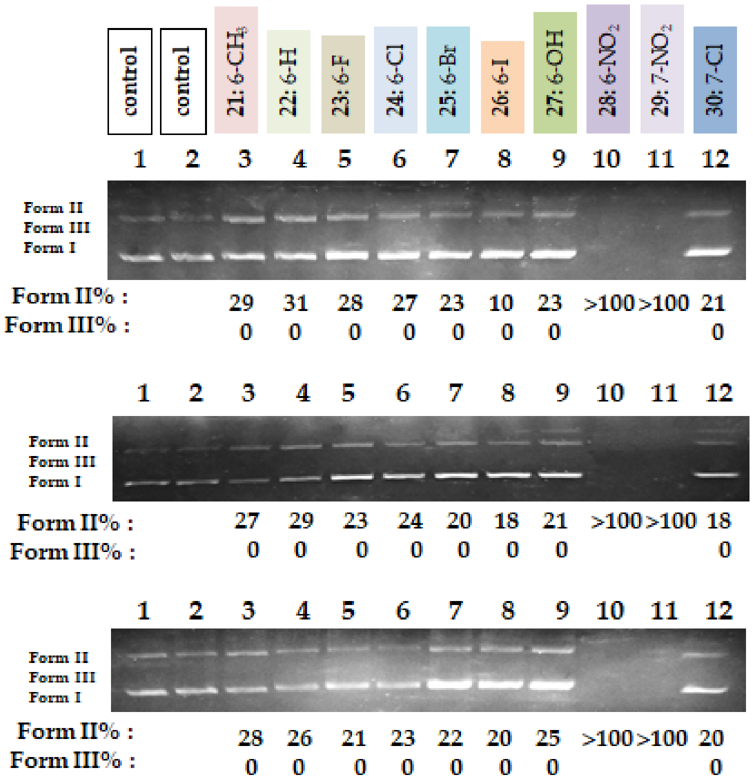

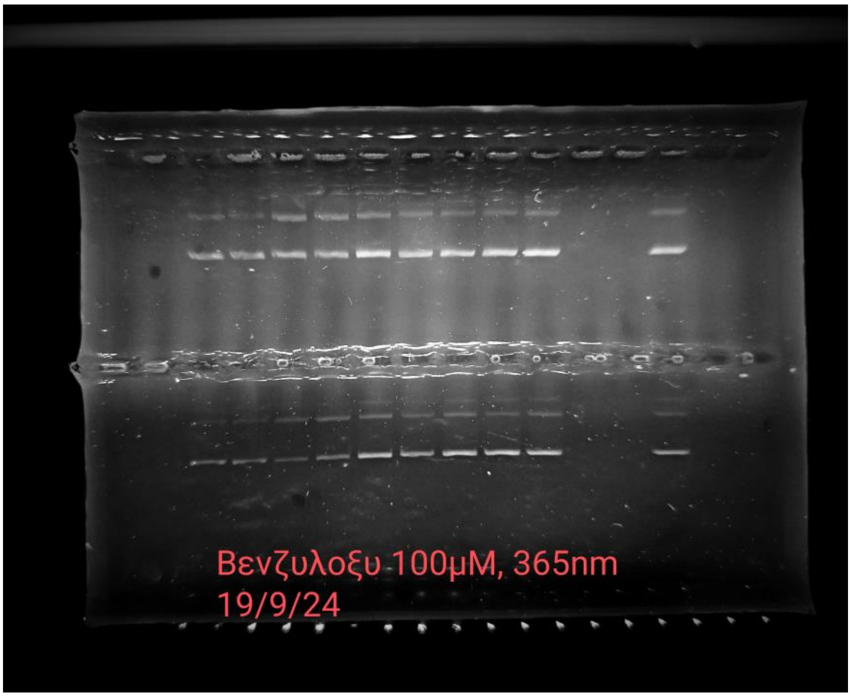

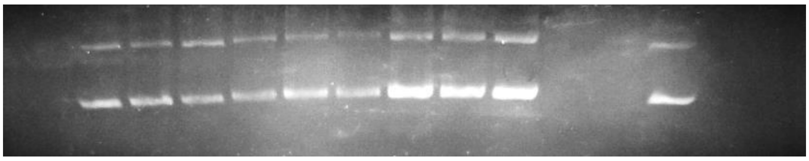

Figure 9A 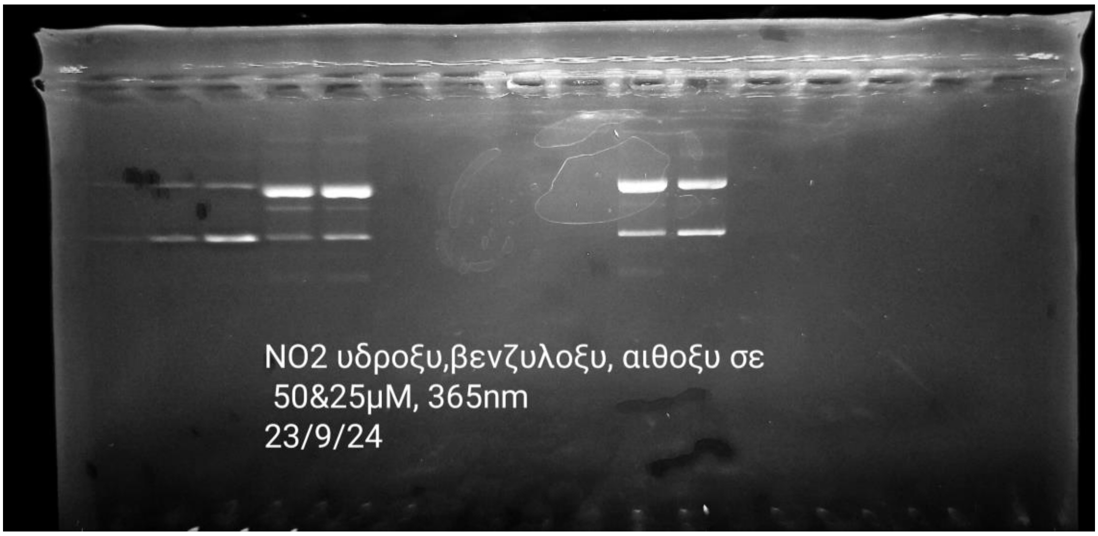

Figure 9B 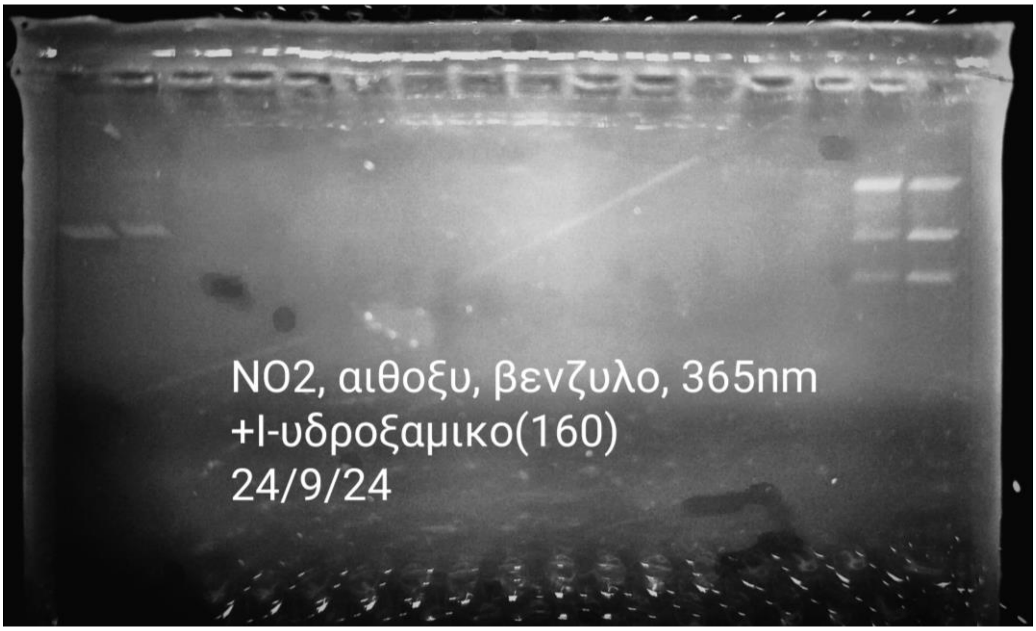

Figure 10A 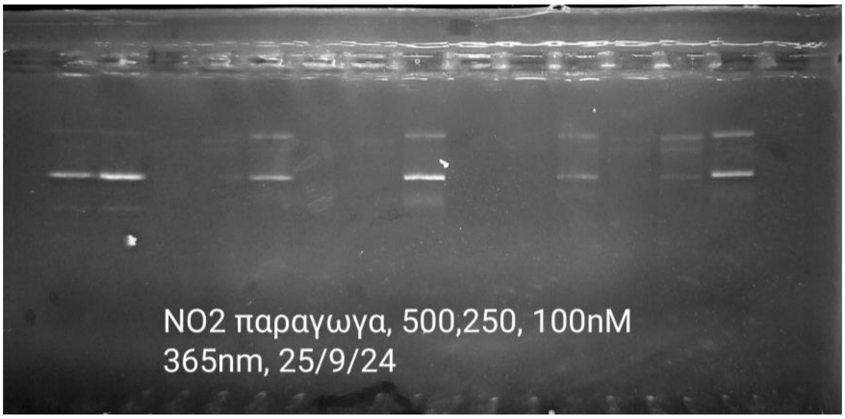

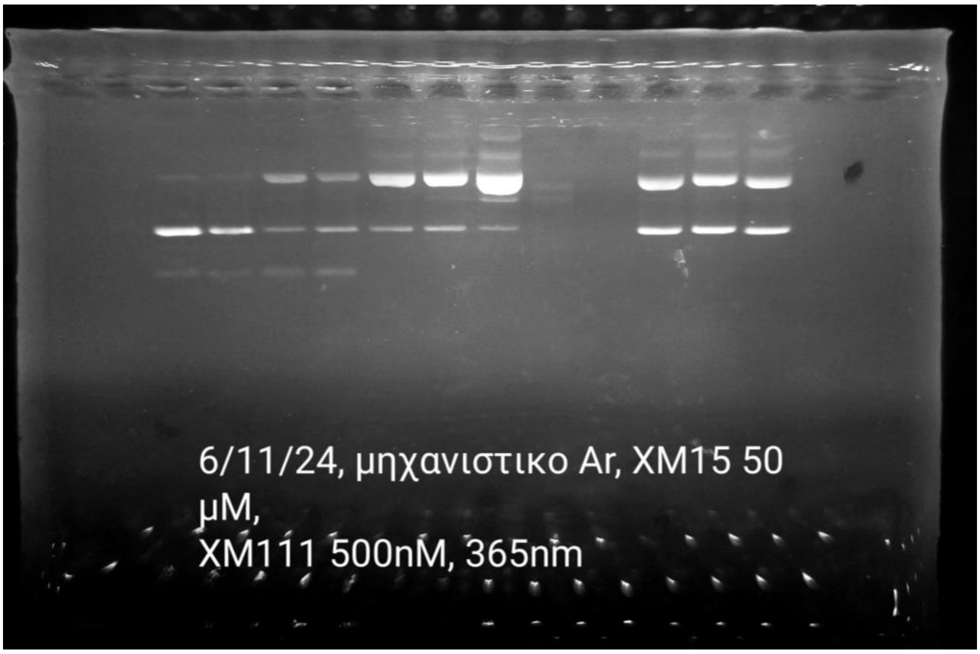

Figure 10B

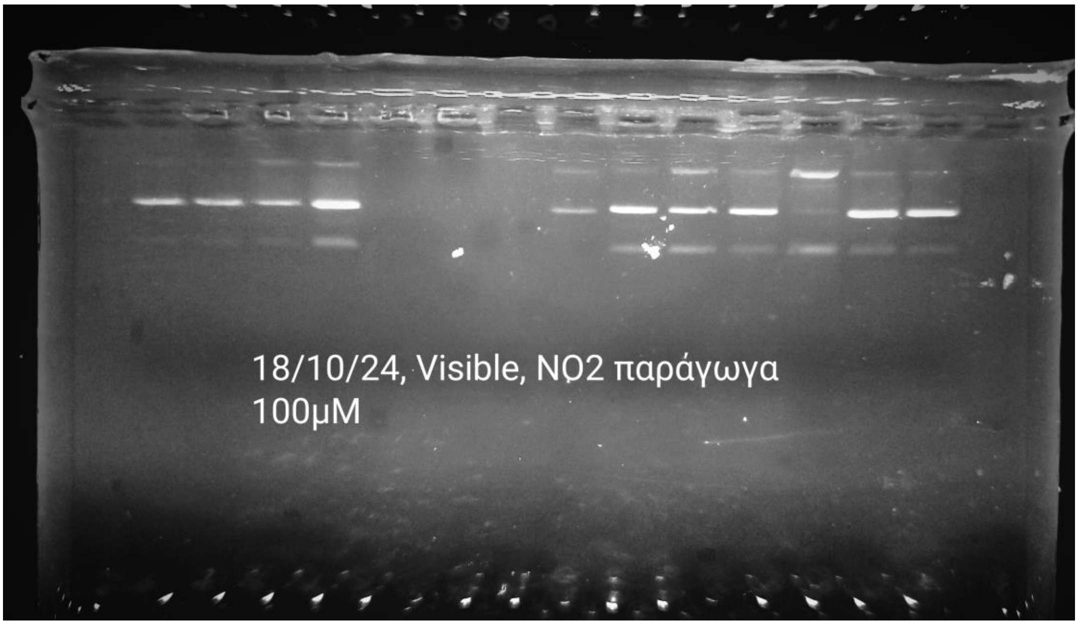

Figure 11A

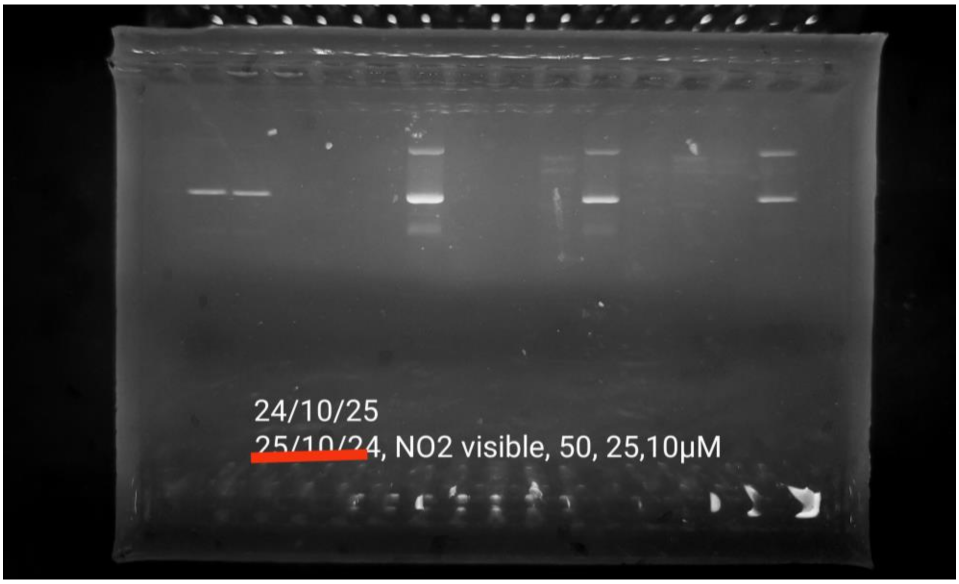

Figure 11B

Figure S6. *First-order plots for oxidation of the DPBF by singlet oxygen, for* ***8***, ***18*** *and* ***28*** *in DMF*

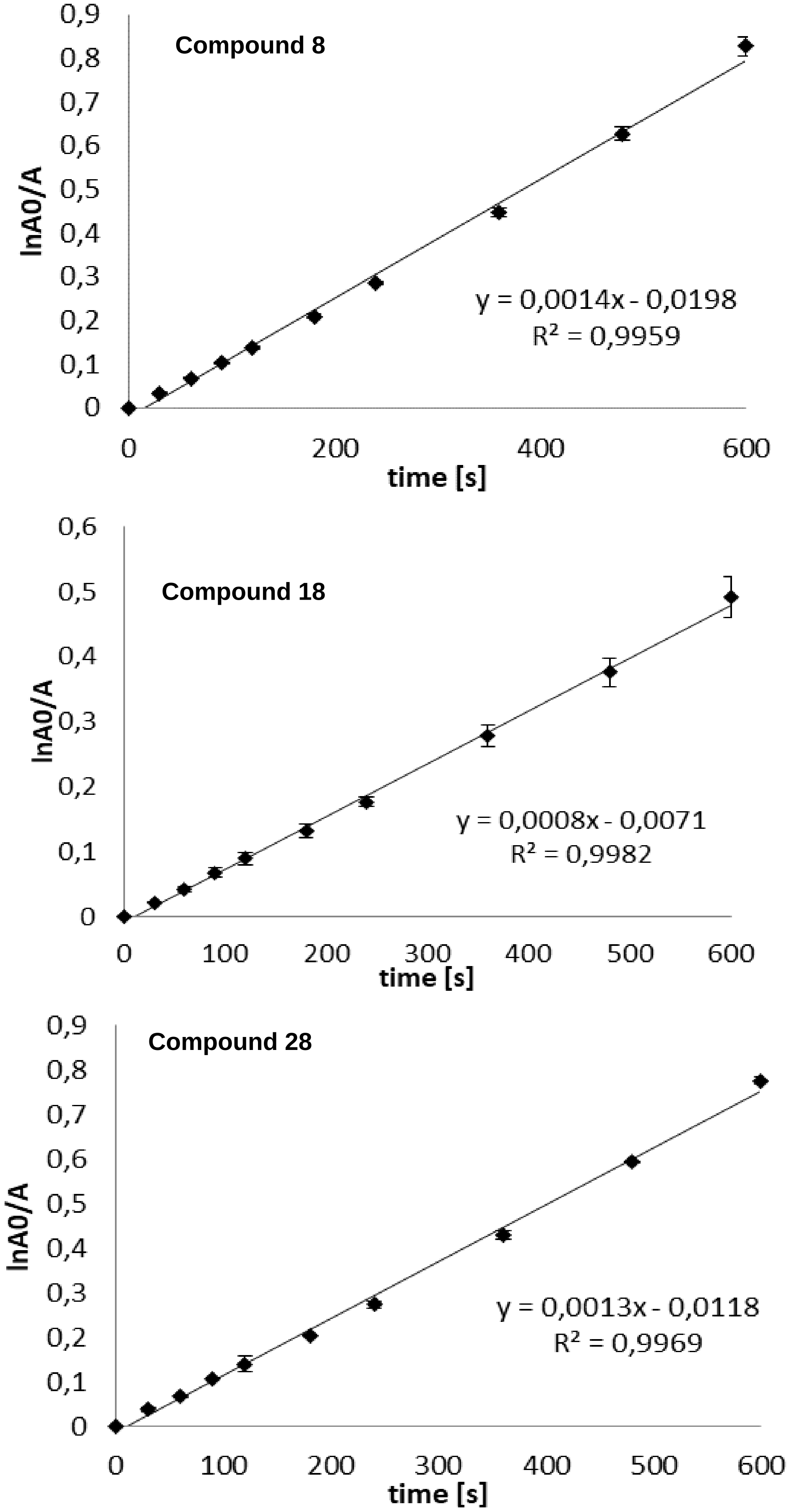

Figure *S7. In silico Molecular Dockings for compounds 1–30 with DNA*

Geometry Optimization of ligands: Gaussian 09, B3LYP/6-31g*.
DNA: 1bna (Protein Data Bank), water molecules removed, Gasteiger charges added. (ADT)
Grid Box: 60 × 80 × 114 with 0.375 Å , (whole DNA)
Docking protocol: rigid
Program: AutoDock Vina
Algorithm: Lamarkian Genetic Algorithm, runs= 100 (exhaustiveness)

Ref. full for Gaussian 09: M. J. Frisch, G. W. Trucks, H. B. Schlegel, G. E. Scuseria, M. A. Robb, J. R. Cheeseman, G. Scalmani, V. Barone, B. Mennucci, G. A. Petersson, H. Nakatsuji, M. Caricato, X. Li, H. P. Hratchian, A. F. Izmaylov, J. Bloino, G. Zheng, J. L. Sonnenberg, M. Hada, M. Ehara, K. Toyota, R. Fukuda, J. Hasegawa, M. Ishida, T. Nakajima, Y. Honda, O. Kitao, H. Nakai, T. Vreven, J. A. Montgomery, Jr., J. E. Peralta, F. Ogliaro, M. Bearpark, J. J. Heyd, E. Brothers, K. N. Kudin, V. N. Staroverov, R. Kobayashi, J. Normand, K. Raghavachari, A. Rendell, J. C. Burant, S. S. Iyengar, J. Tomasi, M. Cossi, N. Rega, J. M. Millam, M. Klene, J. E. Knox, J. B. Cross, V. Bakken, C. Adamo, J. Jaramillo, R. Gomperts, R. E. Stratmann, O. Yazyev, A. J. Austin, R. Cammi, C. Pomelli, J. W. Ochterski, R. L. Martin, K. Morokuma, V. G. Zakrzewski, G. A. Voth, P. Salvador, J. J. Dannenberg, S. Dapprich, A. D. Daniels, Ö. Farkas, J. B. Foresman, J. V. Ortiz, J. Cioslowski, and D. J. Fox, Gaussian 09 (Gaussian, Inc., Wallingford CT, 2009).

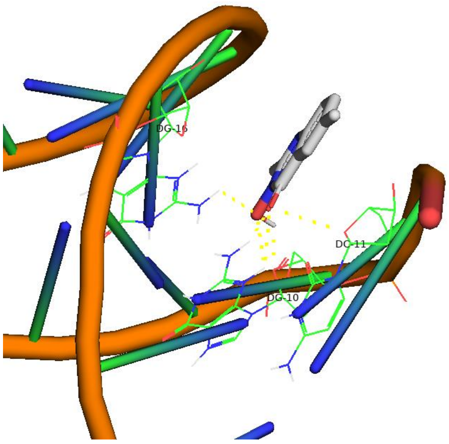

1

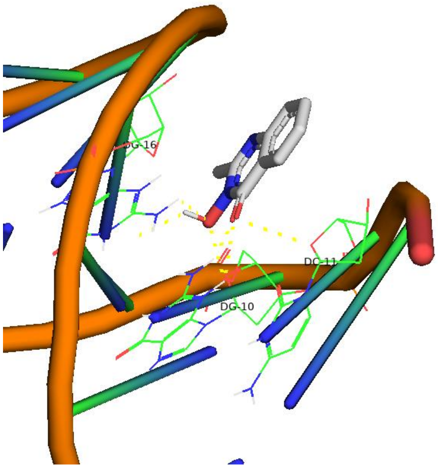

2

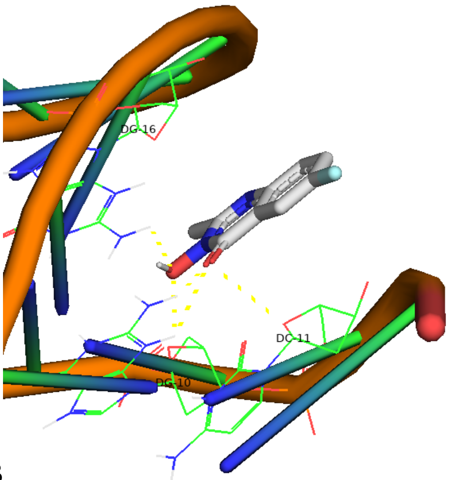
DG-16
DC-11
DG-10

3

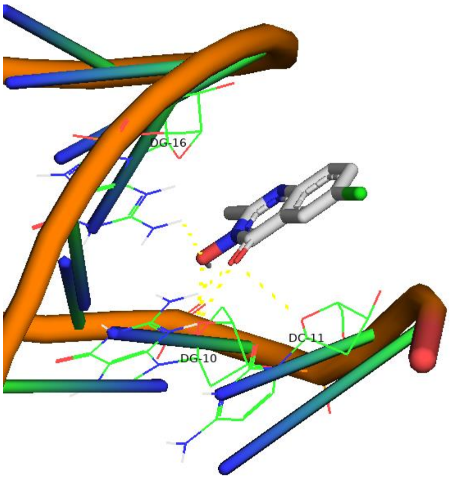
DG-16
DC-11
DG-10

4

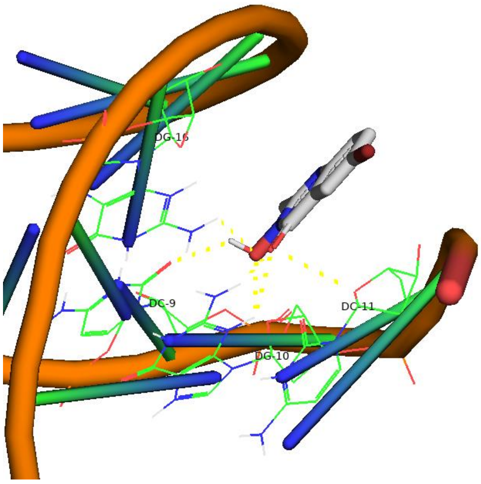
DG-16
DC-9
DC-11
DG-10

5

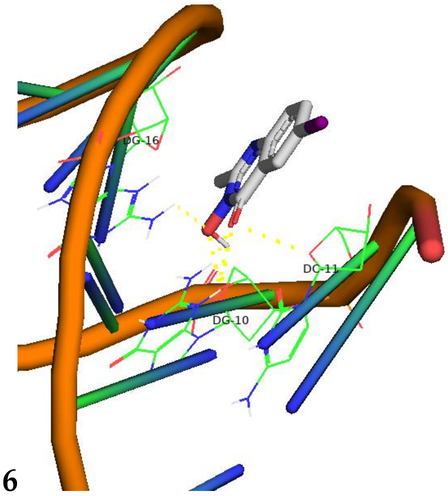
DG-16
DC-11
DG-10

6

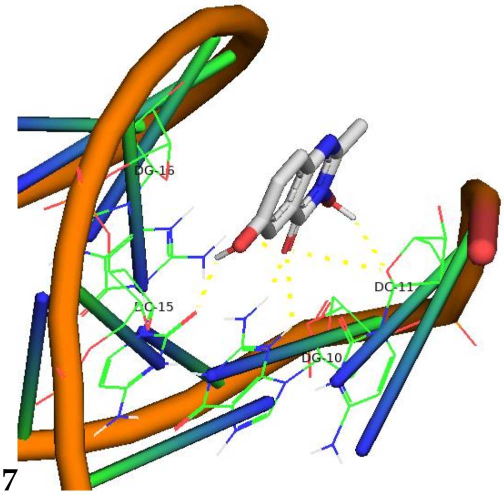
DG-16
DC-15
DC-11
DG-10

7

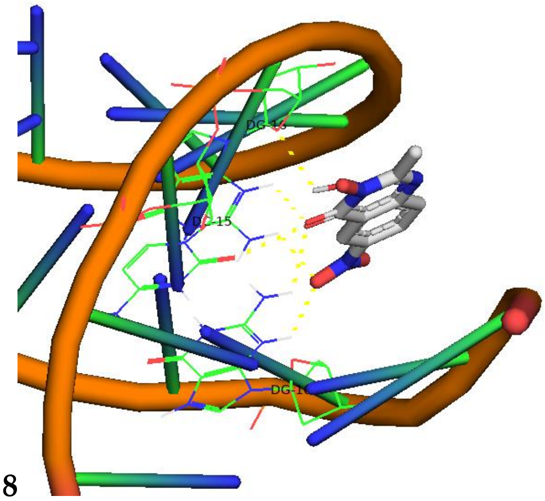

8

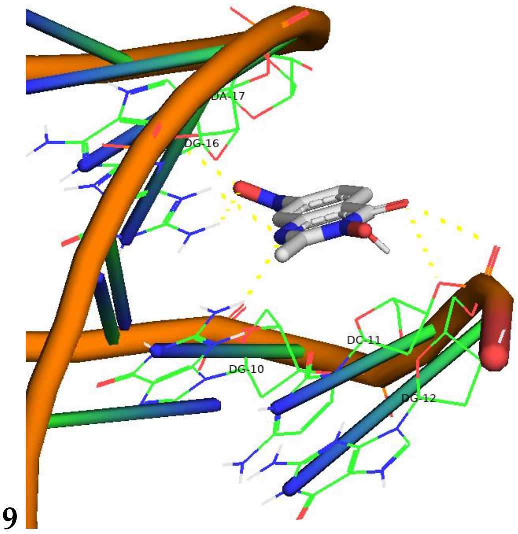

9

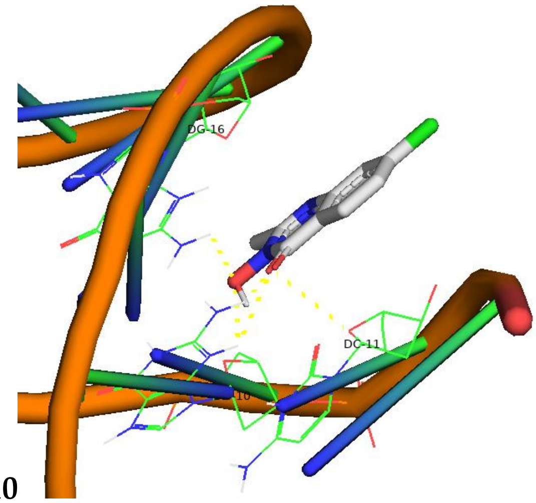

10

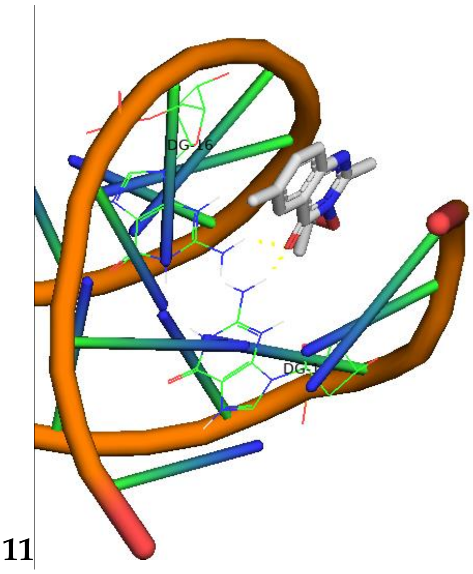

11

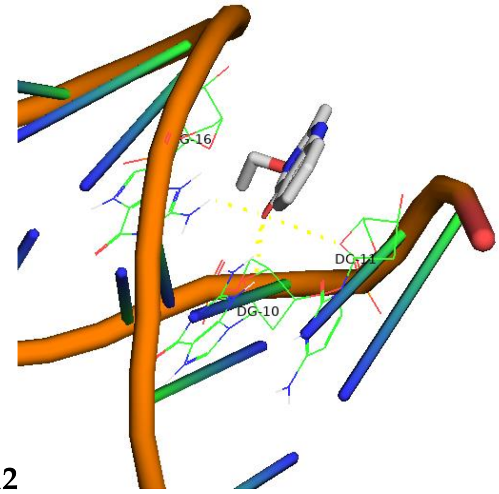

12

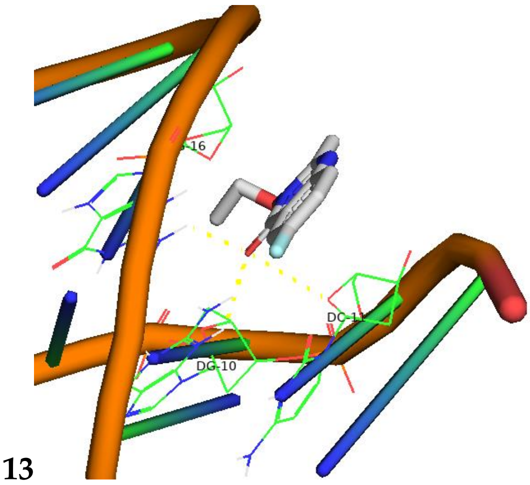

13

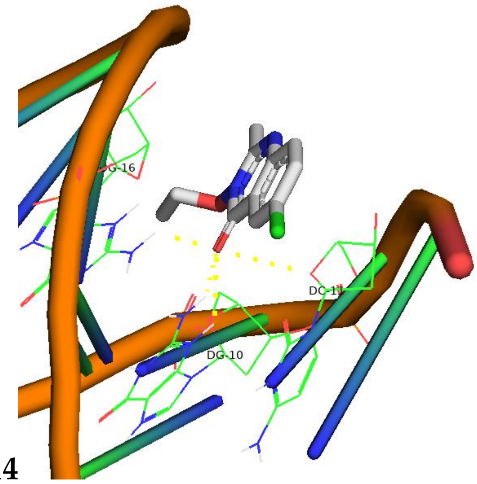

14

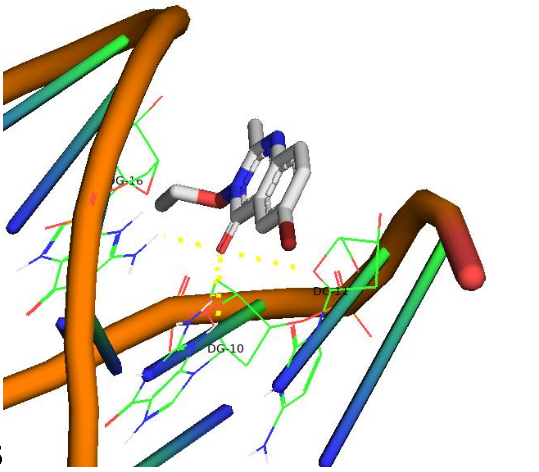

15

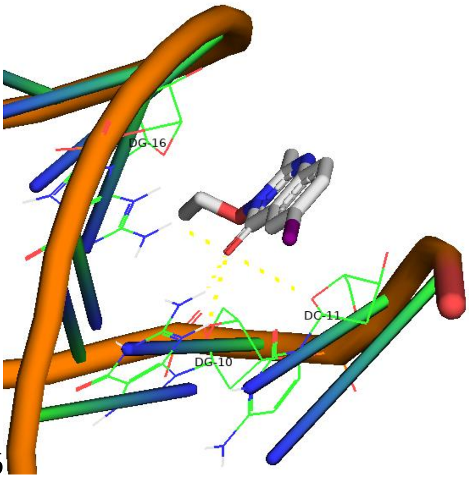

16

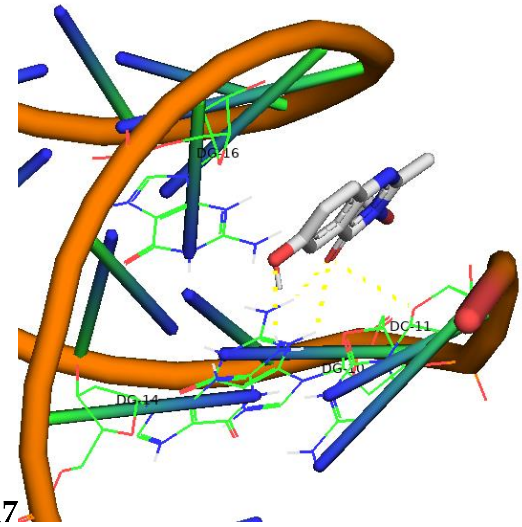

17

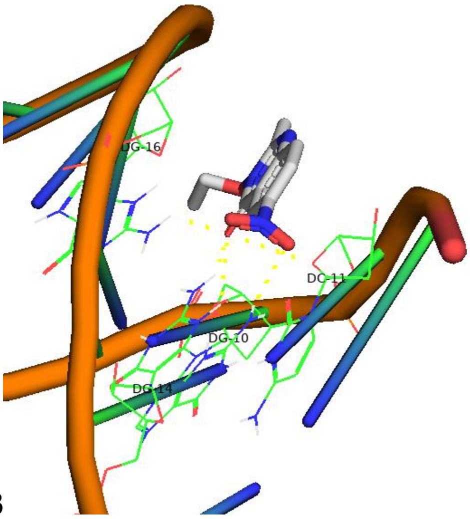

18

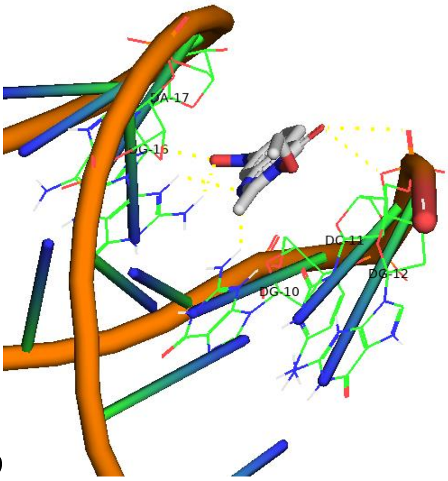

19

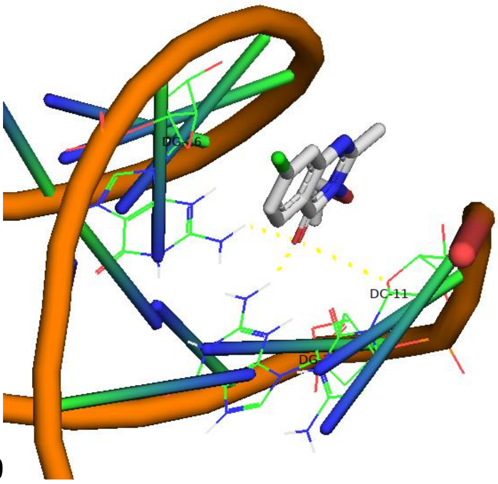

20

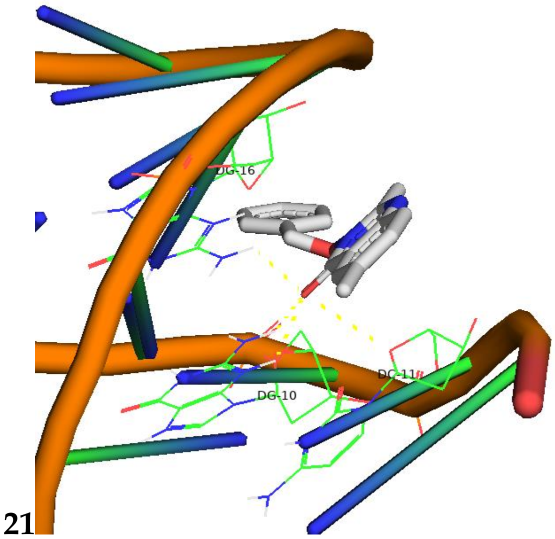

21

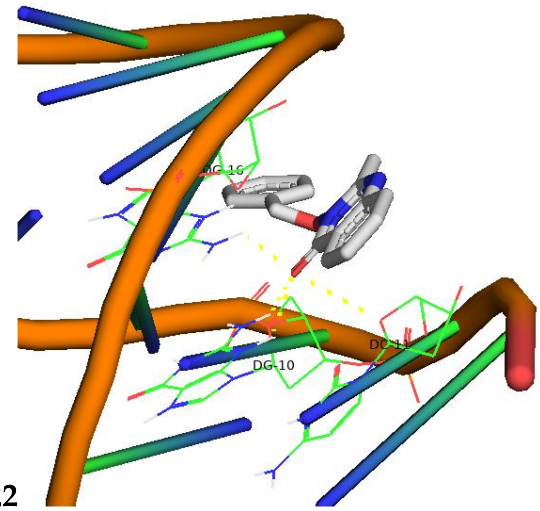

22

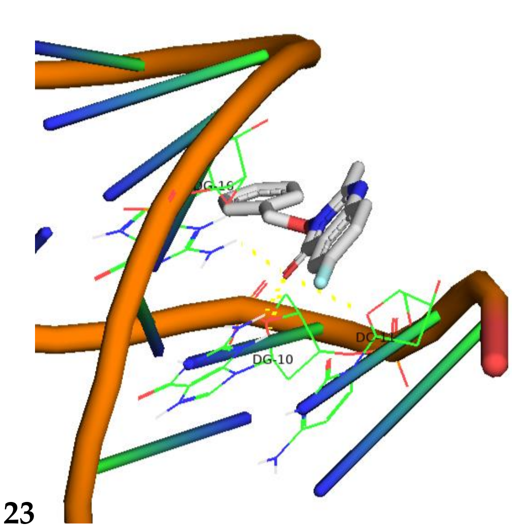

23

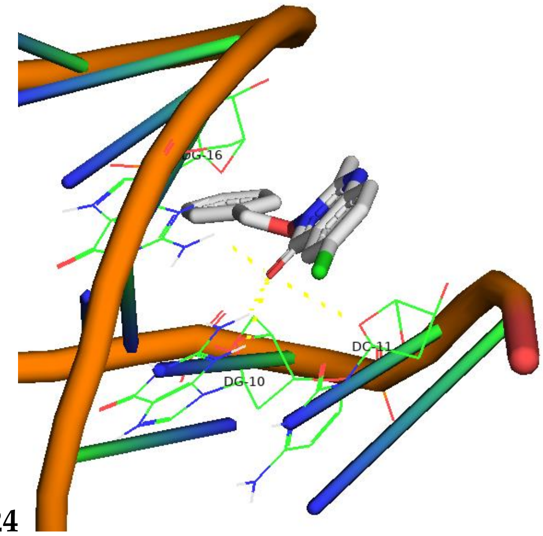

24

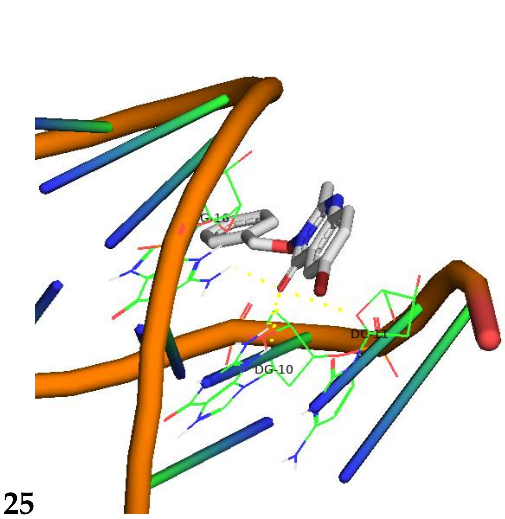

25

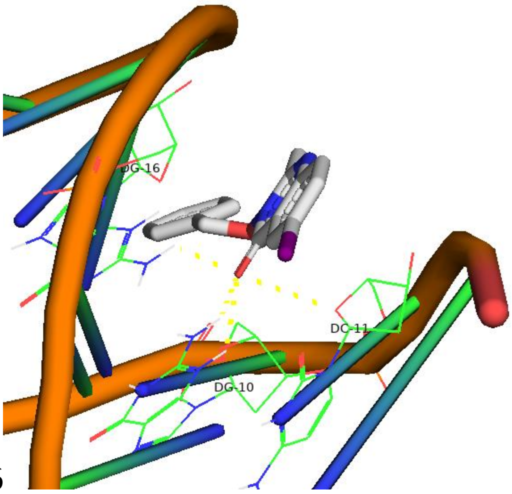

26

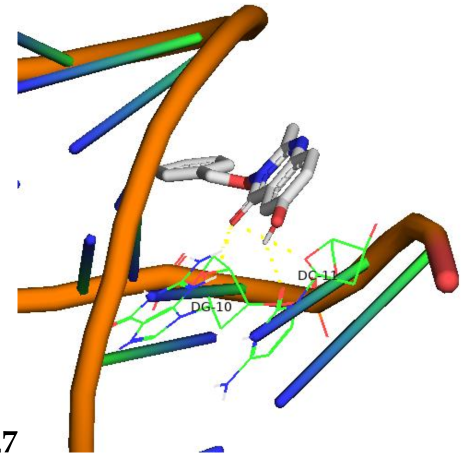

27

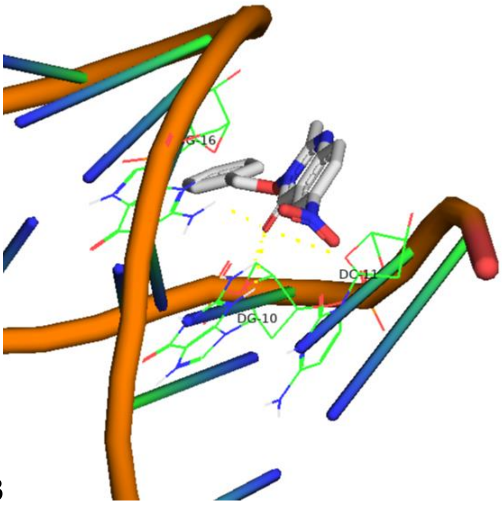

28

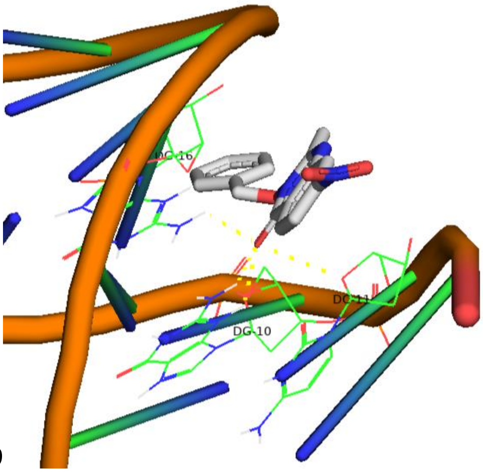

29

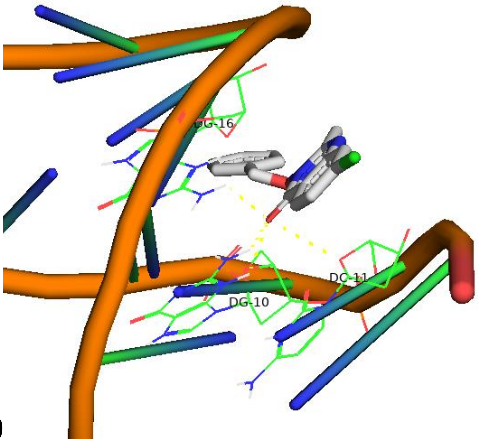

30

Figure S8. Control experiments for all cell lines

(A) Viability test on untreated cells of all 3 cell lines after 1h exposure to UV-A irradiation. (B) Viability test on untreated COLO-800 cells in different timepoints (15, 30, 60 min) of exposure to UV-A irradiation.

(A)

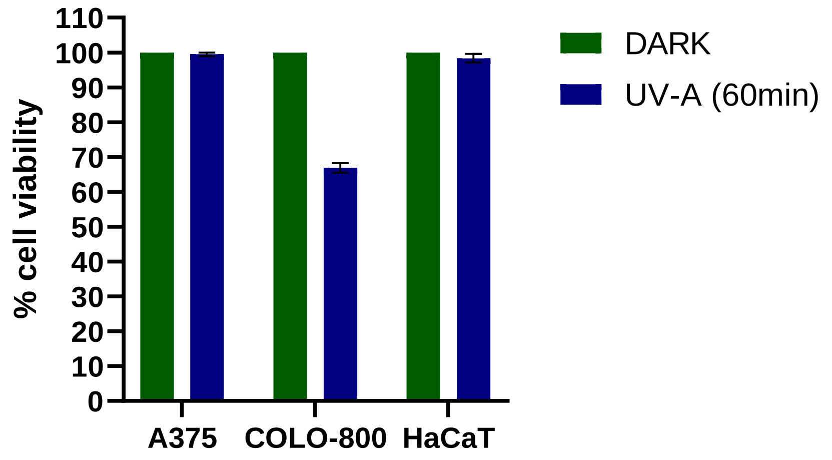

(B)

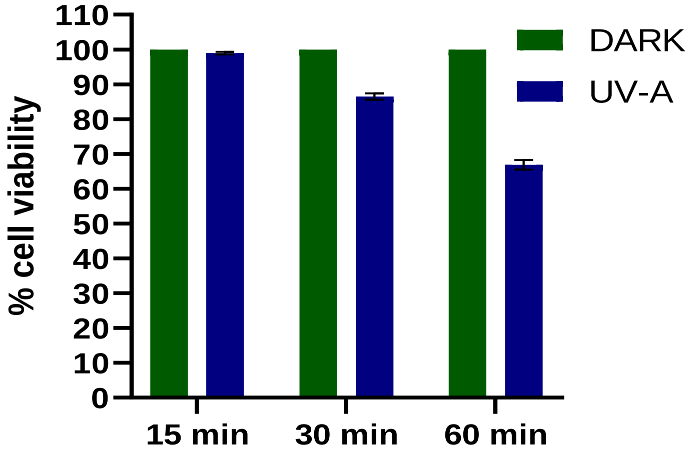

Supplement: Supplementary file 1 [file biomolecules-16-00551-s001.zip › biomolecules-4191685-supplementary.pdf]
